# Fold Designability, Distribution, and Disease 

**DOI:** 10.1371/journal.pcbi.0020040

**Published:** 2006-05-05

**Authors:** Philip Wong, Dmitrij Frishman

**Affiliations:** 1 Institute for Bioinformatics, GSF–National Research Center for Environment and Health, Neuherberg, Germany; 2 Department of Genome-Oriented Bioinformatics, Technische Universität Munchen, Wissenschaftzentrum Weihenstephan, Freising, Germany; European Bioinformatics Institute, United Kingdom

## Abstract

Fold designability has been estimated by the number of families contained in that fold. Here, we show that among orthologous proteins, sequence divergence is higher for folds with greater numbers of families. Folds with greater numbers of families also tend to have families that appear more often in the proteome and greater promiscuity (the number of unique “partner” folds that the fold is found with within the same protein). We also find that many disease-related proteins have folds with relatively few families. In particular, a number of these proteins are associated with diseases occurring at high frequency. These results suggest that family counts reflect how certain structures are distributed in nature and is an important characteristic associated with many human diseases.

## Synopsis

Most proteins are composed of structural domains that can be classified into “folds.” Domains with the same fold type share overall structural similarity. The number of amino acid sequences that encode a fold is termed the “designability” of the fold. Folds that have higher designability are thought to be more robust to stresses and mutations. Such features may also allow the fold to appear in a greater variety of contexts. Here, the authors show that proteins with folds estimated to be of higher designability are more widespread amongst proteins in human, mouse, and yeast, consistent with this hypothesis. The authors also find that many hereditary disease-associated proteins have folds estimated to be of low designability. A number of these diseases occur at a relatively high frequency. These results suggest that the estimate of designability employed reflects how certain structures are distributed in nature and is an important characteristic associated with many human diseases.

## Introduction

Different proteins exhibit a wide range of abilities to functionally withstand the affects of environmental stress or mutation. One property that has been proposed to contribute to protein functional robustness is “designability,” the number of sequences that encode a protein's structure. Using simple lattice models in which proteins are modeled as chains of hydrophobic and hydrophilic residues on lattices, Li et al. [1] has shown that different proteins could have vastly different designabilities. Proteins with more designable structures (i.e., proteins that have more sequences that encode their structures) were proposed to be structurally more robust to mutation and thermal stresses [1–3]. In line with this hypothesis is the finding that proteins of thermophiles exhibited a higher contact trace, a measure that correlates well with the designability, than a sample of mesophiles [4].

**Figure 1:**
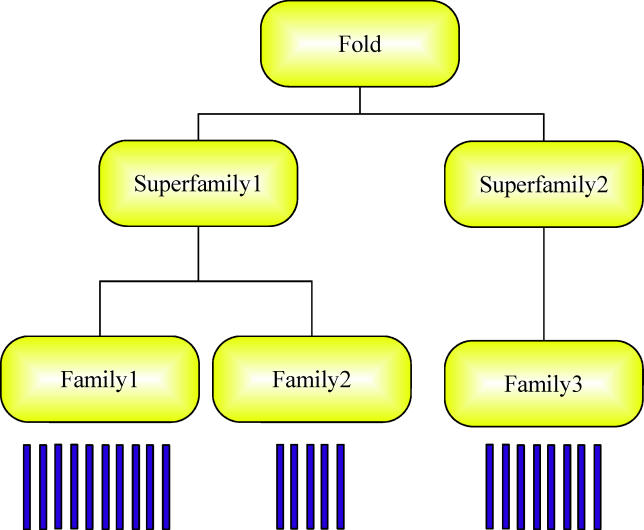
SCOP Hierarchy Four levels of SCOP are shown: fold, superfamily, family, and sequence (dark blue rectangles). The number of sequences is equal to or greater than the number of families, which is equal to or greater than the number superfamilies, which in turn is equal to or greater than the number of folds.

It has been hypothesized that protein structures of higher designability tend to be more fit because such structures would allow a greater amount of sequence changes associated with a greater diversity of function [5]. To assess designability, we took advantage of the hierarchical nature of the Structural Classification of Proteins (SCOP) database (http://scop.mrc-lmb.cam.ac.uk/scop) [6]. In this database, structures with highly similar sequences are grouped into families, families sharing a relatively close common ancestor based on high structural similarity are grouped into superfamilies, and superfamilies sharing an overall structural similarity are in turn grouped into folds. It is clear that in such a classification scheme, the number of sequences is always greater than or equal to the number of families, which are greater than or equal to the number of superfamilies, which is greater than or equal to the number of folds (Figure 1). Since a direct relationship exists between the number of sequences and the number of families in protein folds [2], a rough estimate of fold designability had been defined as the number of families within a fold [7]. This estimate of designability assesses the ability of a fold to withstand mutations based on the level of diversity of associated sequences derived under various functional constraints in its past history. In this work, we compared family counts within folds and the degree to which the structures appeared in eukaryotic proteomes. We subsequently show that families belonging to ancient folds with greater numbers of families tend to be more sequence divergent and more widespread throughout the human, mouse, and yeast proteomes, consistent with the hypothesis that more designable folds should be more fit.

Because mutation or environmental change can disrupt and/or create aberrant function in proteins, and given that a large proportion of mutations seem to affect protein structure [8–14], hereditary disease–related proteins were hypothesized to more often contain structures of relatively low designability as compared to non-disease proteins (proteins without disease annotation). Preliminary work also suggests that the majority of disease-causing mutations tend to be located in structural domains [15]. Protein designability was subsequently estimated by counting the number of families in each domain fold and taking the minimum count. In other words, we assessed the designability of proteins by the fold estimated to be least designable. Using this measure, it was shown that disease proteins tended to have folds with fewer families than non-disease proteins, suggesting that disease propensity of proteins was related to protein designability [7].

In this work, we continue to investigate the concept of fold designability and its connection to hereditary diseases. We estimate protein designability based on the average family counts of all folds in a protein and, subsequently, find that many disease proteins contain folds with relatively few families. In particular, disease proteins were again estimated to be less designable than non-disease proteins, using this measure. We also provide evidence using a database of disease properties that proteins predicted to be less designable are associated with diseases occurring with greater frequency. Taken together, this work provides further evidence that designability is a factor important to our understanding of many of our diseases.

## Results

### Older Folds Have More Families

A potential problem of estimating designability using family counts is that relatively young folds may not have had enough time to establish families, even though the fold may be encoded by large numbers of sequences. Subsequent investigation revealed that relatively ancient folds appearing in both prokaryotes and eukaryotes (see Materials and Methods) have significantly more families than all the SCOP folds in human proteins (Figure S1). Eukaryotic folds found only in human, mouse, and yeast currently contain only approximately 2.5 families, on average, compared to an average of 13.8 families per fold for all human proteins. Thus, time seems to be a significant factor in determining how many families are found in a fold. To minimize the influence of inadequate time for the procreation of fold families on our estimate of designability, we concentrated subsequent investigations on ancient folds. Results pertaining to all human folds are found in the supplementary materials.

### Folds with More Families Tend to Be More Sequence Divergent

**Figure 2:**
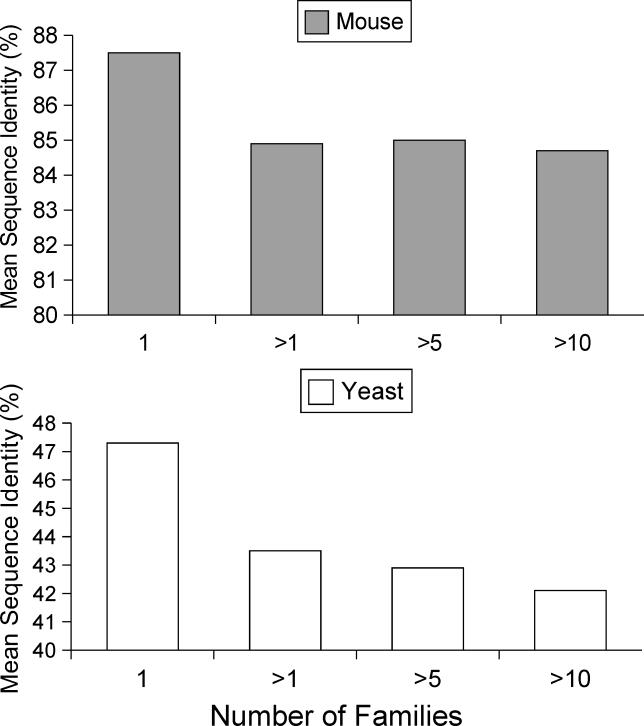
Sequence Divergence and Family Counts of Ancient Folds Ancient SCOP folds found in human proteins were compared to those in mouse and yeast orthologs (see Materials and Methods), and the average divergence was recorded for each fold. The SCOP folds were divided into a number of bins according to the number of families that they contain (*x*-axis). The mean of the sequence identities in each bin is shown (*y*-axis) for mouse and yeast. For both organisms, as the number of families in a SCOP fold increases, the sequences that encode the fold become more divergent. Against mouse orthologs, folds with one family were significantly more conserved than those with more than one family (MW-test, Kolmogorov-Smirnov test [KS-test]: *p <* 0.01). Against yeast orthologs, a significant difference in divergence was observed between folds of one and more than ten families (MW-test, KS-test: *p <* 0.05).

We first compared the sequence divergence among ancient SCOP folds in human proteins against the number of families they contain using protein orthologs in mouse and yeast. Because orthologs were compared, the domains being compared belong to the same family (see Materials and Methods). In general, it was found that families that belong to ancient SCOP folds with greater numbers of families tended to be more sequence divergent (Figure 2). This trend was also observed when we restricted our analysis to SCOP folds created relatively close to the origin of the human–mouse common ancestor (Figure S2). A similar trend was observed when all folds were analyzed, although the significance of the trend could not be established (Figure S3). These results suggest that family counts in folds are associated with the divergence of these folds.

### Ancient Folds with More Families Are More Fit

**Figure 3:**
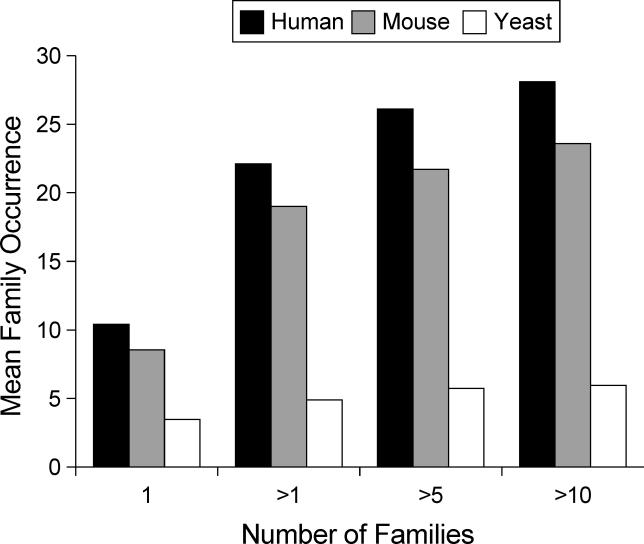
Family Occurrence and Family Counts of Ancient Folds Ancient SCOP folds were divided into a number of bins according to the number of families that they contain (*x*-axis). For each bin, the mean family occurrence (the mean number of proteins in which the SCOP families in these folds appear) for human, mouse, and yeast proteins is shown. As the number of families in a SCOP fold increases, the occurrence of families belonging to these folds in the proteome tends to increase. Significant (MW-test, KS-test: *p <* 0.05) occurrence differences were found between folds of one and more than ten families in human and mouse. No significant differences were detected in yeast. The differences in mean family occurrence between mammals and yeast tend to be larger for folds with larger numbers of families. The differences in mean family occurrence between mammals and yeast tend to be larger for folds with larger numbers of families. These interspecies differences between folds of one family and those of more than one family are significant (MW-test: *p <* 0.1; KS-test: *p <* 0.001).

A possible consequence of higher designability is that of greater fitness. We reason that more designable folds would be more robust to sequence changes associated with a greater diversity of functionality, and thus would be found more often in a proteome in a greater variety of functional contexts. To test if our measure of designability correlates with fold fitness in the eukaryotic proteome, ancient folds from human, mouse, and yeast were binned according to the number of families they contain. For folds in each bin, the number of proteins each fold appeared in was counted and averaged. It was found that ancient folds with greater numbers of families appeared in greater numbers of different proteins (Figure S4). This result was not unexpected because, in general, larger numbers of families tend to be encoded by larger numbers of sequences. Do individual families that belong to folds with more families appear more often in the proteome? For all families that belong to folds in each bin, the number of proteins each family appeared in was also counted and averaged. It was found that families that belonged to ancient folds with greater number of families, appeared in greater numbers of different proteins (Figure 3).

**Figure 4:**
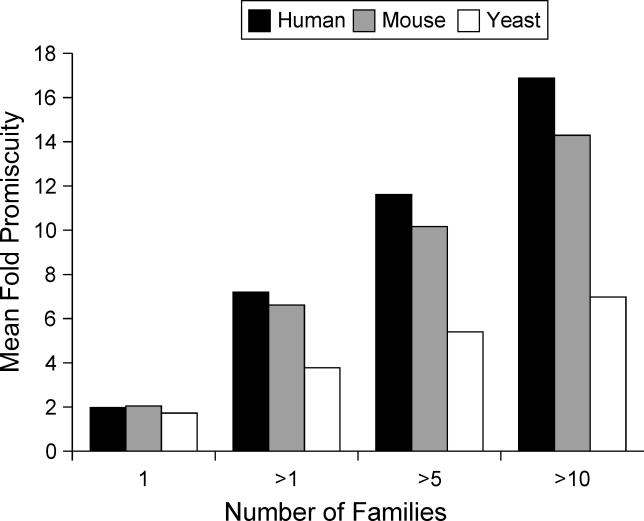
Fold Promiscuity and Family Counts of Ancient Folds Ancient SCOP folds were divided into a number of bins according to the number of families that they contain (x-axis). SCOP folds are connected to other “partner” folds in the same protein. The mean promiscuities (the number of unique partner folds a SCOP fold has) of folds in human, mouse and yeast are plotted. As the number of families in a SCOP fold increases, its promiscuity tends to increase. The differences in fold promiscuity between human, mouse, and yeast are larger for folds with larger numbers of families. All promiscuity differences described here between folds with one family and folds with more than one family are significant (MW-test, KS-test: *p <* 0.02).

A related measure of fold fitness is that of fold promiscuity. We define “fold promiscuity” as the number of unique “partner” folds in the entire proteome, that a particular fold had appeared with in the context of the same protein. We found that ancient folds with more families also tended to be more promiscuous (Figure 4). Graphically, if folds were represented as nodes, and edges connected folds if they were found in the same protein, folds with more families would be more hub-like. These results are consistent with the hypothesis that more designable folds are more fit, because they appear in larger numbers of proteins in a greater variety of sequence contexts. The same trends were observed in M. musculus and S. cerevisiae proteomes.

**Figure 5:**
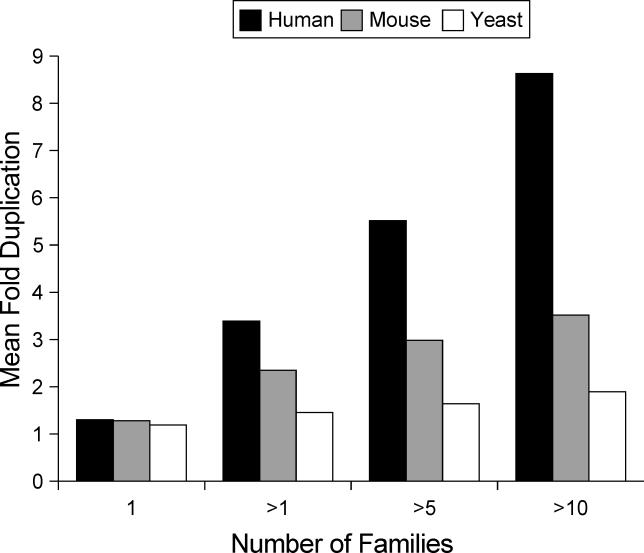
Fold Duplication and Family Counts of Ancient Folds Ancient SCOP folds were divided into a number of bins according to the number of families that they contain (*x*-axis). The maximum number of times each fold is reused in the same protein was counted. The mean count for each bin is shown for human, mouse, and yeast proteins. As the number of families in a SCOP fold increases, the maximum number of times the fold is duplicated in proteins also tends to increase. The differences in magnitude of duplication between folds with one family and folds with more than one family are significant (MW-test, KS-test: *p <* 0.01). The differences in fold duplication between human, mouse, and yeast are larger for folds with larger numbers of families. The differences between folds with one family and folds with more than one family with respect to mammals and yeast are significant (MW-test: *p <* 0.05; KS-test: *p <* 0.001). The significance of the differences between human and mouse could not be established.

A third measure of fold fitness is the number of times that a fold is reused in a given protein. A fold is considered here to be duplicated within the same protein if more than one instance of it exists in that protein. Duplication of folds within a protein allows either for amplification of existing functions associated with such folds or creation of new functions. Folds with different functionality are likely encoded by different sequences. Sequence dissimilarity may also be selected for in folds cooperating to amplify a single function because individual folds must function in different spatial contexts. Indeed, of the 3,468 human proteins that have duplicate folds, less than 7% (230/3,468) have such folds detected with the same BLASTP E-value (see Materials and Methods). Sufficient dissimilarity may also help sequences avoid aggregation [16]. More designable folds would allow for a greater variety of sequence change necessary for viable duplications. Examination of our human proteins revealed that ancient folds with more families are reused more often in the same protein (Figure 5). Although statistical significance could not be established, SCOP families (structures that are likely to be closely related) that belong to more folds with more families were observed to be duplicated more often in the same protein (Figure S5). The same trends were observed in M. musculus and S. cerevisiae proteomes.

Observations across multiple genomes allow one to compare the occurrence of associated phenomena within the timeframe delineated by the divergence of these genomes. Three different proteomes belonging to modern human, mouse, and yeast have emerged from the time of the common ancestor of these organisms. Within this fixed timeframe, the proteomes leading up to modern mammals have expanded considerably compared to those leading up to the yeast proteome. In particular, we found that the magnitude of differences between yeast and mammals in terms of fold occurrence, promiscuity, and duplication is much higher for ancient folds with larger numbers of families (Figure S4; Figures 4 and 5). These differences were also noticeable between human and mouse (Figures 3–5; Figure S4). The top ten folds that have expanded the most are listed in Protocol S1. It seems that given the opportunity or need, structures of higher designability can proliferate more throughout proteomes, through events such as horizontal transfer, duplication, and recombination, within a fixed evolutionary timeframe. Taken together, these results provide further evidence that ancient folds estimated to be more robust to sequence or environmental change can be found more often in different contexts within eukaryotic proteomes.

Although our investigation has focused thus far on the divergence and proliferation of ancient folds, we also detect similar trends when we examined all folds and families within these folds (Figures S3–S12). Statistical significance of the trends in some cases could not be established. Interestingly, similar trends were also observed amongst gamma-proteobacteria (Figures S13–S17), suggesting that the relationships between family counts, fold divergence, and proliferation are not specific only to eukaryotes.

### Increased Age Does Not Imply Increased Fold Divergence and Proliferation

Thus far, older folds were found to have more families, and folds with more families were found to be more divergent and widespread. Thus, a possible explanation as to why folds with more families are more divergent and widespread within genomes is that these folds tend to be older. To test this hypothesis we compared ancient folds and young folds found only in human and mouse (see Materials and Methods) in terms of these attributes. In contrast to what we expected, we found that ancient folds tend to be more conserved than young folds (Figure S18). No statistically significant differences in terms of occurrence (Figure S19) and duplication within proteins (Figure S20) could be found between young and old folds. Ancient folds were found to be marginally more promiscuous than young folds (Figure S21). At the family level, ancient families were found to be significantly less abundant, promiscuous, and duplicated than young families found only in mouse and human (Figures S18–S21). Families belonging to ancient folds were also found to be less abundant, promiscuous, and duplicated than those belonging to young folds (unpublished data). These results are opposite to what we have hypothesized, suggesting that increased age does not necessarily elevate divergence and proliferation levels of protein structures.

### Little Correlation between Family Counts and Fold Length

The number of sequences that encode a fold has been hypothesized to be related to the length of the fold [5]. In contrast to this hypothesis, we found little correlation between the fold length and family counts (Figure S22).

### Designability and Disease

Using the SCOP hierarchy, the designability of proteins was estimated in two ways: (1) as the number of families in the fold predicted to be least designable [7] and (2) as the mean family count across all detected folds in proteins in which SCOP had high coverage (see Materials and Methods). Although the second method reduces the number of proteins available for analysis, it ensures that most residues in the protein chain contribute to the designability measure. Using both methods, disease proteins were predicted to be less designable on average than non-disease proteins (Table 1), with a disproportionate number of disease proteins having folds containing only one family (Protocol S2).

**Table 1:**
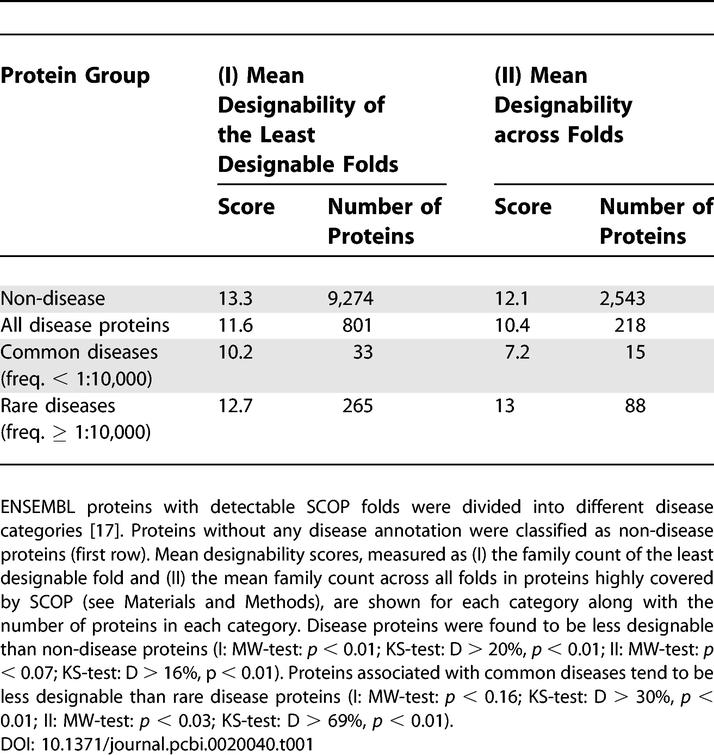
Designability and Disease Frequency

Although biased toward the populations assessed and limited in quantity, data pertaining to disease frequency [17] were also examined. Proteins associated with common diseases were predicted to be less designable than proteins associated with rare diseases (Table 1; Protocols S3–S4). Although a relatively high *p*-value was obtained with the Mann-Whitney (MW-test) when comparing designability values generated with the first method, a much lower *p-*value was obtained using the second method in which proteins analyzed had high SCOP coverage.

## Discussion

In this and a previous work [7], we estimated SCOP fold designability by the number of families found within that fold. In other words, we estimate the number of sequences that encode a fold by its level of divergence achieved in its past history. One confounding factor for this measure is that the number of families found within a fold could depend on the time the fold existed [18]. We found that ancient folds had significantly more families than relatively young folds found only in mouse and human. Thus, certain relatively young folds may exist, which may be highly designable, may not have had enough time for different families to evolve, and thus would be predicted to be less designable by our measure. Furthermore, since SCOP is largely based on manual annotation of known protein structures, a bias exists in the classification in terms of what sequences have been mapped to structures [19] and how these sequences are classified into folds. It is not known what effect this bias has on the use of family counts to estimate designability. The question arises as to whether family counts reflect what we expect to be properties of more designable structures and whether these properties are observable in nature.

To ensure adequate time was available for family procreation among folds to be examined, we concentrated our analysis on relatively ancient folds found both in eukaryotes and prokaryotes. Protein families belonging to ancient folds containing more families were found in larger numbers of proteins. Ancient folds with greater numbers of families were also found in partnership with a more diverse set of other folds, and were duplicated more often within the same protein. In particular, the expansion of families belonging to ancient folds with more families was found to be greater since the time of the yeast–mouse–human common ancestor. These results are also consistent with the hypothesis that folds with more families are more designable. More designable folds would allow for a larger number of sequence changes in a fold, allowing for greater diversity of function. This line of thought concerning protein folds is analogous to recent findings that designability correlates with contact density, which correlates with the mean functional flexibility score of gene families [18]. A fold that can exhibit greater functionality would be at an evolutionary advantage because it can appear in a greater variety of contexts. A strong correlation between fold promiscuity and occurrence suggests that fold recombination occurs nearly randomly [20]. Other factors such as evolutionary history or the need for functions associated with a fold or fold combinations seem to have also influenced the frequency in which certain folds appear in proteomes [6,18,20,21] (see Protocol S5). Our finding that folds with more families are more abundant and promiscuous in proteomes does reflect the expected increased fitness of more designable folds. It lends to speculation that designability is an important factor affecting how folds are distributed within proteomes and their potential for evolution of new functions via sequence mutations. Although exceptions exist, the assumption that the number of families that belong to a given fold is a good estimate of the fold's evolutionary success [5] appears to be largely valid.

Fold designability defines a limit to the divergence associated with folds. Interestingly, we find that sequences belonging to folds with greater numbers of families were more divergent in orthologous proteins. Clearly, selection would affect the divergence of folds. However, if family counts capture the designability of a fold, these results also suggest that designability may have contributed significantly in limiting the divergence of folds. It would be highly interesting to tease apart structural and selective influences on divergence in the future.

Because older folds have more families, and folds with more families are more widespread and divergent, one might presume that folds are more widespread and divergent simply because they are older. We found that ancient folds were not necessarily more widespread than young folds in terms of occurrence and duplication, but were found to be more promiscuous, perhaps due to greater opportunity for recombination. Ancient folds were also found not to be more divergent than young folds. Abeln and Deane [22] have also previously noted that old folds do not necessarily have many copies in genomes. The relationship between time and fold divergence is similar to previous findings that older mammalian proteins tend to be more conserved [23]. Thus, our analysis revealed that the greater divergence, occurrence, and duplication observed for folds with more families is not simply an artifact of their tendency to be older.

Our inability to find a relationship between length of folds and the number of families contained within folds may be the result of the limited number of structures known. It also raises speculation that increasing the length of folds does not necessarily increase their designability, perhaps due an increase in the potential for aberrant misfolding and aggregation [7].

It is worth noting that the associations between family counts, divergence, and fold proliferation in genomes were statistically weakened when we considered all folds instead of just ancient folds. This phenomenon is consistent with the idea that relatively young folds may not have had enough time to procreate families, thus obscuring trends between these attributes.

Early structural characterization of the human proteome indicated significant differences in SCOP superfamily composition between disease and non-disease proteins [24]. In this and a previous work [7], it was found that disease proteins tend to have folds with fewer families than non-disease proteins (see Protocol S2). One cannot rule out that such a trend is a product of bias in our disease data. Nevertheless, about one third of folds in disease proteins with only one family are relatively ancient folds (see Materials and Methods), and it is a mystery why many families have not evolved. Our results do suggest that many disease proteins have structures that have been relatively sequence constrained throughout their evolution. The form of this constraint may have involved the lowering of the fitness of ancestral organisms upon mutation of such structures. If similar constraints are maintained in humans for a sufficient number of proteins, then this may help explain why disease proteins tend to have folds with fewer families. Two-thirds of folds in disease proteins, however, are relatively young (most of which are found only in mouse and human) and thus would be predicted to be relatively less designable by contact-residue–based measure of designability [18] compared with ancient folds.

Interestingly, we found that within a database of disease properties, more frequently occurring diseases were associated with proteins containing folds with fewer families. Theoretically, proteins with less-designable folds would be less robust to mutation. Such a characteristic suggests two reasons why proteins predicted to be less designable have been associated with more common diseases. First, proteins with lower structural robustness would be more likely to receive disease-associated mutations. Our results lend to speculation that an increased chance for deleterious mutations in proteins predicted to be less designable have contributed to their association with more frequently occurring diseases. Second, one would also expect the diversity in terms of structure and stability of less-robust proteins to be greater in a population. Such diversity in structure or stability is likely correlated with functional diversity because proteins of different structures usually perform different functions, and proteins of different stabilities would likely have different cellular lifetimes. Such diversity would facilitate the survival of a population in rapidly changing environments because certain members are more likely to contain a mutation adapted to the new environment. These mutations, however, may cause disease directly or increase susceptibility to diseases. For example, over 100 mutations in *G6PD* that increase the risk of hemolytic anemia may provide resistance to malaria outbreaks [25,26]. In such a scenario, less-designable proteins would become associated with common diseases because members with mutations in these proteins would be more likely to survive. Subsequent population expansion would increase the number of individuals with disease-prone proteins, and bottlenecks would increase the frequency with which these diseases occur in populations [27].

Analogous to a decrease in designability, an increase in length has been proposed to increase the likelihood of a protein receiving disease-causing mutations [28]. Notably, diseases that occur more frequently have also been associated with longer proteins [29]. Longer proteins may also possess a greater diversity of mutations in a population, thus conferring functional diversity, especially if the mutations are distributed among different domains that carry out different functions. Like with less-designable proteins, it is possible that certain large proteins have been associated with common diseases because they had a greater chance of possessing a disease-causing mutation that happened to be beneficial in the past.

What has not been considered so far is the propensity for precursor molecules encoding proteins to undergo disease causing alterations. For example, certain genomic contexts or hotspots have been identified that predispose DNA sequences for mutation [30,31]. If these sequences happen to encode proteins of low designability, then such proteins would be associated with diseases of greater occurrence. For example, the gene associated with Gaucher's disease has pseudogenes that predispose the gene for disease-causing gene conversion events [32]. The associated protein contains the gycosyl hydrolase fold, a fold with only three known families. Thus, a protein with a fold seemingly constrained against sequence divergence has been associated with the increased potential for mutation associated with pseudogenes. Another example involves the genes *OPN1LW* and *OPNL1MW* encoding red and green photopigment proteins containing the Family AG protein–coupled receptor-like fold (f.13) that has only two known families. Their high sequence similarity and tandem arrangement have been thought to predispose these genes for disease-causing recombination events [33]. Such associations between folds of low designability and genomic contexts that increase mutation propensity suggest that maintenance of polymorphism in corresponding genes has been selected for, perhaps to ensure differences amongst individuals that confer advantages to the population. This reasoning may help explain why certain duplicate genes are retained [34,35]. In the case of vision-related genes, variation in perception may help groups exploit a wider range of niches [36,37]. What selective advantage Gaucher's disease gene polymorphism would confer remains an open question. Interestingly, recent comparisons between disease and non-disease genes revealed a significant excess of highly polymorphic genes constrained for divergence associated with disease [38]. The association between folds with fewer families with increased mutability in encoding regions may explain in part this observation.

To what extent intrinsic susceptibility to mutation and selection on populations has contributed to the association of diseases with proteins with few families is not known. The former mechanism suggests that a larger number of disease alleles exists for more common diseases. The latter mechanism suggests that a small number of common disease alleles in the population account for the high frequency of occurrence in human diseases [27]. Comparisons of disease allele frequencies between common and rare diseases when sufficient data become available may shed more light on this phenomenon. Whatever the dominant mechanism may be, the results of comparing length and fold family counts against disease frequency do suggest a general principle that properties that increase a protein's propensity for disease association would also increase the frequency that the associated diseases would appear in certain populations.

We must emphasize however, that fewer than 300 proteins with SCOP folds detected from our Ensembl database have been mapped to disease frequency categories. Disease frequency is influenced by many factors, including environmental effects and underlying genotypes. Thus, the disease frequency data we use may not reflect certain populations. Although we have provided evidence for an association between disease and SCOP family counts, the nature of this association is not known for many proteins. The extent to which our explanations hold as to why diseases are associated with proteins predicted to be less designable remains to be assessed. Establishment of principles based on our work would require further investigation on larger datasets accounting for multiple factors that influence disease propensity.

Throughout this work, we have extensively used family counts as an estimate of fold designability. How many sequences can encode a protein fold depends, not only on the intrinsic constraints imposed by the geometry of the fold, but also on the external environment. For example, high temperatures could restrict the sequence space occupied by folds [4], whereas the presence of chaperones [39–41] and proteases [42] could do the opposite. These molecules allow sequences more likely to misfold to exist by preventing or reversing misfolding events (in the case of chaperones) or degrading the fraction of deleterious misfolded sequences (in the case of proteases). Family counts may capture, not only the intrinsic designability of a fold, but also the degree of success of the fold within the multitude of environments and fitness constraints experienced throughout the history of the fold. The use of family counts to estimate fold designability assumes that the past success of a fold implies its future designability.

A major disadvantage of using family counts is that it is an imprecise measure. It is likely that different proteins with the same folds, and hence the same family count scores, can have vastly different designabilities. Moreover, if the fold is relatively young, the number of families contained in that fold may be too small to reflect its designability. Although residue contacts [18], oligomerization states [43], and molecular interactions are not direct measures of sequence divergence success, these properties are more specific to individual proteins and may help distinguish designability differences between proteins with similar folds. Data on gene mutation frequency and expression [44–46] may also prove useful in predicting designability. To this day, no comprehensive experiments have been conducted to investigate the relationship between different folds and their ability to withstand environmental stress or mutation. Screening random sequences for ones that fold is one approach [5] to investigate designability. Testing the foldability and functionality of different proteins after mutagenesis in different environments (in vivo or in vitro) is another approach [47–50]. Such experiments are likely to provide new information important to refining measures of designability and its use to estimate fold fitness. It would be highly interesting to repeat experiments carried out in this work using a greater variety of designability measures.

In summary, we have provided evidence that family counts can capture characteristics of fold designability. By estimating fold designability, we suggest explanations regarding how folds are distributed in proteomes and their potential for evolution. We have also provided evidence that our measure of protein designability is associated with properties of diseases. The designability concept remains immature [5], but our work suggests that it may already have practical applications. With further development of this concept, further insights into the evolution of proteins and designability's relation to diseases are anticipated.

## Materials and Methods

### Proteomes and disease annotation.

A total of 34,111 proteins predicted to be encoded in the human genome were obtained from the Ensembl human v23.34e.1 database [51]. OMIM-based [52] disease annotations for human genes were obtained using the EnsMart tool [53] and mapped to 2,113 proteins in the Ensembl protein dataset. OMIM is a database focused mostly on heritable genetic diseases of high penetrance. For inter-species comparisons, the PEDANT [54] mouse and yeast [55] genomes encoding 42,049 and 6,723 proteins, respectively, were used. Common and rare disease annotation was taken from Jimenez-Sanchez et al. [17]. The associated proteins, covering a wide variety of phenotypes, are listed in Protocols S3 and S4.

### Protein fold assignments.

Protein SCOP [6] (December 2004) assignments were obtained from the PEDANT system. SCOP folds were assigned to proteins if the corresponding sequences were within a BLASTP [56] E-value of 10^−6^. Our conclusions did not change when an E-value threshold of 10^−2^ was chosen instead (unpublished data). For the analysis of disease proteins, only the largest protein encoded by each gene was included as done by López-Bigas and Ouzounis [28].

### Ancient folds and families.

Folds identified in human and more than six other genomes *(Bacteroides fragilis* NCTC9434, *Mus musculus, Deinococcus geothermalis, Escherichia coli* K12, Vibrio fischeri ES114, Psychrobacter arcticum 273 4, *Chlorobium vibrioforme, Sulfolobus acidocaldarius* DSM, *Saccharomyces cerevisiae, Mycoplasma hyopneumoniae* 232, *Streptococcus zooepidemicus,* and *Anabaena variabilis)* were considered to be relatively ancient. Similarly, we defined relatively ancient families as those families appearing in human and six other genomes. Our conclusions did not change if we considered domains appearing in human and two other genomes (one of which is a prokaryote) as ancient.

### Sequence divergence and fold designability.

**Figure 6:**
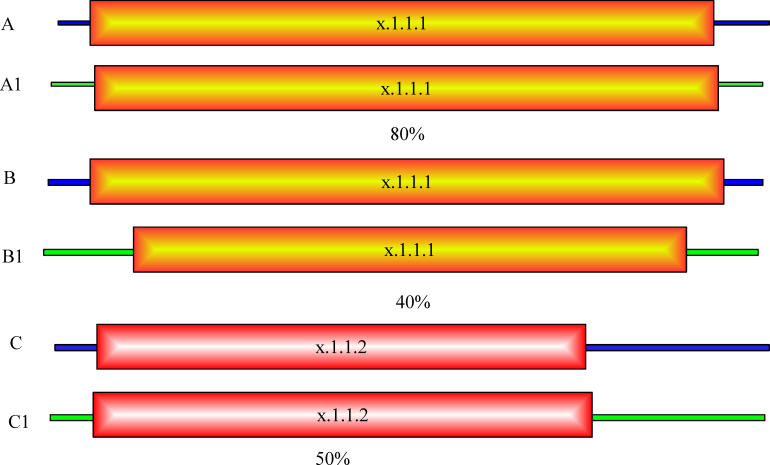
Average Divergence Calculations The average divergence of the fold is calculate as follows: Let one genome encode the proteins A, B, and C and another genome encode the proteins A1, B1 and C1. Let domain F be the hypothetical SCOP fold x.1 in proteins A, A1, B, B1, C, and C1. (A, A1), (B, B1) and (C, C1) are orthologous pairs. Fold x.1 contains only two families, denoted x.1.1.1 and x.1.1.2. The domains (that belong to the same family) are aligned. The average divergence for families x.1.1.1 and x.1.1.2 is (40% + 80%)/2 = 60% and (50% + 50%)/2 = 50%, respectively. The average divergence for fold F would be taken as the mean of the average divergence of all its families, namely (60% + 50%)/2 = 55%.

To test whether the number of families found within folds correlated with their divergence, the number of families associated with each SCOP fold was compared with the sequence divergence of that fold. Individual SCOP domains from human proteins were aligned with the corresponding domains found within corresponding mouse and yeast protein orthologs, and the sequence identity was recorded. Protein orthologs between human, yeast, and mouse were identified as bidirectional best BLASTP hits with exactly the same SCOP domains. Note that the strict ortholog definition in use ensures that the SCOP domains being compared belong to the same SCOP family. Sequence identity between the SCOP families were computed using ClustalW [57] with default parameters. Subsequently, the divergence for each SCOP fold was measured by computing the average divergence of individual SCOP families within that SCOP fold and then taking the average (see Figure 6 for a detailed example). We term this result the “average divergence” of a SCOP fold. By comparing domains between ortholog pairs, we hoped to minimize the potential for large sequence divergence contributions due to functional differences between proteins of vastly different functions.

For human and mouse comparisons, we also defined orthologs as those genes encoding proteins with the same SCOP families and that are bidirectional best hits with at least one nearby gene being a bidirectional best hit to a gene nearby its ortholog. We define nearby genes of gene A as those genes within five genes of A. Using this orthology definition, we obtained similar results (unpublished data).

### Protein designability measures.

Protein designability was measured as done in Wong et al. [7], by counting the number of families in each SCOP fold contained in a given protein and taking the minimum. For example, if protein A contains three domains with folds F1, F2, and F3 and these folds in turn contain eight, three, and seven families, respectively, protein A's minimum family count would be three. By recording the minimum family count of the folds in proteins, we assessed their designability by assessing the designability of their least designable fold.

We also assessed protein designability using a measure that ensures most residues that take part in structural domains in the protein chain contribute to the designability score. This was done by measuring designability by the mean number of families across all folds in each protein. In our example, the score for this measure would be (8 + 3 + 7)/3 = 6. The SCOP folds detected in this work, however, do not necessarily cover the entire protein, simply because either no such structure has been solved yet or the non-covered regions may be intrinsically disordered. Thus, we apply this measure to only those proteins that do not have regions longer than 70 amino acids that have not been covered by our SCOP detection methods.

### Fold binning.

Because of substantial scattering within plots, analysis was conducted using four bins to emphasize trends: Folds containing only one family, more than one family, more than five families and more than ten families. The last three bins overlap with each other.

## Supporting Information

All supporting information is available to download as a combined file called Combined Supporting Information.

Figure S1 Number of Families and Fold Age The mean number of families found in all, ancient (see Materials and Methods), and human/mouse/yeast-only folds are shown. The mean number of families in all three groups are significantly different from each other (MW-test, KS-test: *p* < 0.01) with ancient folds having the most families. Standard deviations of family counts are 19, 21, and 3 amongst all, ancient, and human/mouse/yeast folds, respectively.
(22 KB PDF)

Figure S2 Divergence of SCOP Folds and Families within a Time Interval SCOP domains found in Ensembl human proteins likely to be mammalian in origin were compared to orthologous domains in mouse, and the average divergence was recorded (see Materials and Methods). Only domains found in human and mouse and not in yeast or a number prokaryotes (see Materials and Methods) were considered likely to be mammalian in origin. The SCOP folds were divided into a number of bins according to the number of families that they contain (*x*-axis). The mean of the sequence identities associated with domains in each bin is shown (*y*-axis). At the fold level (black bars), folds with more than one family were more divergent than folds containing only one family. This trend is considered marginally significant (MW-test: *p* < 0.05, KS-test: *p* < 0.15). At the family level, families belonging to folds with more than one family were more divergent than those belonging to folds containing only one family (white bar). This trend was found to be significant (MW-test, KS-test: *p* < 0.01). Similar trends were observed when we considered only domains found in human, mouse, and yeast. However, significance could not be established for unknown reasons.
(25 KB PDF)

Figure S3 Mean Sequence Divergence and Family Counts of All Folds SCOP folds found in Ensembl human proteins were compared to those in mouse and yeast orthologs, and the average divergence was recorded for each fold (see Materials and Methods). The SCOP folds were divided into a number of bins according to the number of families that they contain (*x*-axis). The mean of the sequence identities in each bin is shown (*y*-axis) for mouse and yeast. For mouse, as the number of families in a SCOP fold increases, the sequences that encode the fold become more divergent. For yeast, a relatively sharp drop in sequence identity scores occurs beyond a family count of ten. Statistical significance of the trends could not be established.
(27 KB PDF)

Figure S4 Fold Occurrence and Family Counts of Ancient Folds Ancient SCOP folds were divided into a number of bins according to the number of families that they contain (*x*-axis). For each bin, the mean fold occurrence (the number of proteins the SCOP folds appear in) for human, mouse, and yeast proteins is shown. As the number of families in a SCOP fold increases, its occurrence in the proteome tends to increase. The differences in fold occurrence between human, mouse, and yeast are larger for folds with larger numbers of families. The differences between folds with one family and folds with more than one family within and between the three eukaryotes are significant (MW-test, KS-test: *p* < 0.001).
(24 KB PDF)

Figure S5 Family Duplication and Family Counts on Ancient Folds Ancient SCOP folds were divided into a number of bins according to the number of families that they contain (*x*-axis). The maximum number of times SCOP families in each fold bin were reused in the same protein was counted. For each bin, the mean count for human, mouse, and yeast proteins is shown. As the number of families in a SCOP fold increases, the maximum number of times families belonging to that fold is duplicated in proteins also tends to increase. However, the significance of this trend could not be established. The differences in SCOP family duplication between human, mouse, and yeast are larger for folds with larger numbers of families, but significance of this trend could not be established.
(24 KB PDF)

Figure S6 Family Occurrence and Family Counts of All Folds SCOP folds were divided into a number of bins according to the number of families that they contain (*x*-axis). For each bin, the mean family occurrence (the mean number of proteins in which the SCOP families in these folds appear) for human, mouse, and yeast proteins is shown. As the number of families in a SCOP fold increases, the occurrence of families belonging to these folds in the proteome tends to increase, although statistical the significance of this trend could not be established. The differences in mean family occurrence between mammals and yeast tend to be larger for folds with larger numbers of families. These differences between folds of one family and those of more than one family were significant (MW-test: *p* < 0.1; KS-test: *p* < 0.001).
(25 KB PDF)

Figure S7 Fold Promiscuity and Family Counts of All Folds SCOP folds were divided into a number of bins according to the number of families that they contain (*x*-axis). SCOP folds are connected to other “partner” folds in the same protein. The mean promiscuities (the number of unique partner folds a SCOP fold has) of folds in human, mouse, and yeast are plotted. As the number of families in a SCOP fold increases, its promiscuity tends to increase. The differences in fold promiscuity between human, mouse, and yeast are larger for folds with larger numbers of families. All promiscuity differences shown here between folds with one family and folds with more than one family are significant (MW-test, KS-test: *p* < 0.05).
(24 KB PDF)

Figure S8 Family Promiscuity and Family Counts of Ancient Folds SCOP folds were divided into a number of bins according to the number of families that they contain (*x*-axis). SCOP families are connected to other “partner” families in the same protein. The mean promiscuities (the number of unique partner families a SCOP family has) of families in human, mouse, and yeast are plotted. As the number of families in a SCOP fold increases, the promiscuity of its families tends to increase. However, of the three species, only human family promiscuity differences between folds with one family and folds with more than one family were found to be significant (MW-test, KS-test: *p* < 0.07). The differences in family promiscuity between human, mouse, and yeast are larger for folds with larger numbers of families. The family promiscuity differences between folds of one and more than one family between mammals and yeast were found to be significant (MW-test, KS-test: *p* < 0.05).
(24 KB PDF)

Figure S9 Family Promiscuity and Family Counts of All Folds SCOP folds were divided into a number of bins according to the number of families that they contain (*x*-axis). SCOP families are connected to other “partner” families in the same protein. The mean promiscuities (the number of unique partner families a SCOP family has) of families in human, mouse, and yeast are plotted. As the number of families in a SCOP fold increases, the promiscuity of its families tends to increase. The differences in family promiscuity between human, mouse, and yeast are larger for folds with larger numbers of families. Statistical significance of the trends could not be established.
(23 KB PDF)

Figure S10 Fold Duplication and Family Counts of All Folds SCOP folds were divided into a number of bins according to the number of families that they contain (*x*-axis). The maximum number of times each fold is reused in the same protein was counted. The mean count for each bin is shown for human, mouse, and yeast proteins. As the number of families in a SCOP fold increases, the maximum number of times the fold is duplicated in proteins also tends to increase. The differences in magnitude of duplication between folds with one family and folds with more than one family are significant (MW-test, KS-test: *p* < 0.05). The differences in fold duplication between human, mouse, and yeast are larger for folds with larger numbers of families. The increase in fold duplication in mammals compared with yeast between folds with one family and folds with more than one family are significant (MW-test: *p* < 0.05; KS-test: *p* < 0.001). The differences between human and mouse were much less pronounced achieving significance only between folds of one family and those of more than ten families (MW-test: *p* < 0.1; KS-test: *p* < 0.001).
(25 KB PDF)

Figure S11 Family Duplication and Family Counts of All Folds SCOP folds were divided into a number of bins according to the number of families that they contain (*x*-axis). The maximum number of times SCOP families in each fold bin were reused in the same protein was counted. For each bin, the mean count for human, mouse, and yeast proteins is shown. As the number of families in a SCOP fold increases, the maximum number of times families belonging to that fold is duplicated in proteins also tends to increase. However, the significance of this trend could not be established. The differences in SCOP family duplication between human, mouse, and yeast are larger for folds with larger numbers of families. These differences were found to be significant between folds of one and more than one family (MW-test, KS-test: *p* < 0.05).
(23 KB PDF)

Figure S12 Fold Occurrence and Family Counts of All Folds SCOP folds were divided into a number of bins according to the number of families that they contain (*x*-axis). For each bin, the mean fold occurrence (the number of proteins the SCOP folds appear in) for human, mouse, and yeast proteins is shown. As the number of families in a SCOP fold increases, its occurrence in the proteome tends to increase. The differences in fold occurrence between human, mouse, and yeast are larger for folds with larger numbers of families. The differences between folds with one family and folds with more than one family within human and between the three eukaryotes are significant (MW-test, KS-test: *p* < 0.001).
(24 KB PDF)

Figure S13 Mean Sequence Divergence and Family Counts of Ancient Folds Ancient SCOP folds found in E. coli proteins were compared to those in Vibrio vulnificus YJ016 and Yersinia pseudotuberculosis IP32953 orthologs, and the average divergence was recorded for each fold (see Materials and Methods). The SCOP folds were divided into a number of bins according to the number of families that they contain (*x*-axis). The mean of the sequence identities in each bin is shown (*y*-axis). For both *Vibrio* and *Yersinia,* as the number of families in a SCOP fold increases, the sequences that encode the fold become more divergent. The difference in conservation between folds of one family and those of more than one family are significant (MW-test: *p* < 0.02; KS-test: *p* < 0.01). With respect to *E. coli–Yersinia* divergence, similar significant trends (MW-test, KS-test: *p* < 0.04) were also observed when all folds were considered. With respect to divergence of all folds between E. coli and the more distant *Vibrio* species, similar trends were observed, but significance could not be established.
(23 KB PDF)

Figure S14 Fold Occurrence and Family Counts of γ-Proteobacterial Folds Ancient SCOP folds were divided into a number of bins according to the number of families that they contain (*x*-axis). For each bin, the mean fold occurrence (the number of proteins in which the SCOP folds appear) for *E. coli, Y. pseudotuberculosis* IP32953, and V. vulnificus YJ016 proteins is shown. As the number of families in a SCOP fold increases, its occurrence in the proteome tends to increase. The differences in fold occurrence between *E. coli, Yersinia,* and *Vibrio* are larger for folds with larger numbers of families. The differences between folds with one family and folds with more than ten families within each bacteria and between the three bacteria are significant (MW-test, KS-test: *p* < 0.02).
(24 KB PDF)

Figure S15 Family Occurrence and Family Counts of γ-Proteobacterial Folds SCOP folds were divided into a number of bins according to the number of families that they contain (*x*-axis). For each bin, the mean family occurrence (the mean number of proteins, the SCOP families in these folds appear in) for *E. coli, Y. pseudotuberculosis* IP32953, and V. vulnificus YJ016 proteins is shown. As the number of families in a SCOP fold increases, the occurrence of families belonging to these folds in the proteome tends to increase. Significant (MW-test: *p* < 0.1, KS-test: *p* < 0.01) occurrence differences were found between folds of one and more than one family in E. coli and *Yersinia*. The trend for V. vulnificus was found to be much weaker (MW-test: *p* < 0.19, KS-test: *p* < 0.01).
(23 KB PDF)

Figure S16 Fold Promiscuity and Family Counts of γ-Proteobacterial Folds SCOP folds were divided into a number of bins according to the number of families that they contain (*x*-axis). SCOP folds are connected to other “partner” folds in the same protein. The mean promiscuities (the number of unique partner folds a SCOP fold has) of folds in *E. coli, Y. pseudotuberculosis* IP32953, and V. vulnificus YJ016 are plotted. As the number of families in a SCOP fold increases, its promiscuity tends to increase. The differences in fold promiscuity between *E. coli, Y. pseudotuberculosis* IP32953, and V. vulnificus YJ016 are larger for folds with larger numbers of families. The promiscuity differences between and within each bacteria between folds with one family and folds with more than ten families are significant (MW-test, KS-test: *p* < 0.02).
(22 KB PDF)

Figure S17 Family Promiscuity and Family Counts of γ-Proteobacterial Folds SCOP folds were divided into a number of bins according to the number of families that they contain (*x*-axis). SCOP families are connected to other “partner” families in the same protein. The mean promiscuities (the number of unique partner families a SCOP family has) of families in *E. coli, Y. pseudotuberculosis* IP32953, and V. vulnificus YJ016 are plotted. As the number of families in a SCOP fold increases, the promiscuity of its families tends to increase. Mean family promiscuity between folds of one family and folds of more than one family were found to be significant for E. coli (MW-test: *p* < 0.05, KS-test: *p* < 0.01). For *Yersinia* and *Vibrio,* the difference was statistically much weaker (MW-test: *p* < 0.2, KS-test: *p* < 0.01).
(901 KB PDF)

Figure S18 Sequence Divergence and Fold Age SCOP folds in human (*x*-axis) were compared to orthologs in mouse and the mean sequence identity (*y*-axis) was recorded (see Materials and Methods). In all comparisons, ancient folds (see Materials and Methods) were found to be significantly more sequence conserved than young folds found only in human and mouse (MW-test, KS-test: *p* < 0.01).
(23 KB PDF)

Figure S19 SCOP Domain Occurrence and Fold Age The occurrence of ancient SCOP domains (see Materials and Methods) in the human proteome was compared to that of young domains (appearing only in human and mouse). Ancient families were significantly less abundant than young families (MW-test, KS-test: *p* < 0.01). At the fold level, no statistical difference in occurrence could be established between ancient and young folds.
(106 KB PDF)

Figure S20 SCOP Domain Duplication and Fold Age The number of times ancient SCOP domains (see Materials and Methods) were duplicated in the same human protein was compared to that of young domains (appearing only in human and mouse). Ancient families were significantly less duplicated than young families (MW-test: *p* < 0.05, KS-test: *p* < 0.01). At the fold level, no statistical difference in duplication could be established between ancient and young folds.
(28 KB PDF)

Figure S21 SCOP Domain Promiscuity and Fold Age The promiscuity of ancient SCOP domains (see Materials and Methods) in the human proteome was compared to that of young domains (appearing only in human and mouse). Ancient families were significantly less promiscuous than young families (MW-test: *p* < 0.1, KS-test: *p* < 0.01). At the fold level, ancient folds were more promiscuous than young folds (MW-test: *p* < 0.07, KS-test: *p* < 0.01).
(23 KB PDF)

Figure S22 Length and Number of Families The number of families in ancient SCOP folds detected in human proteins were plotted against their length. Little correlation (*R^2^* = −0.1) was found. A similar relation with little correlation between length and family counts was found when all human folds were included in the analysis (unpublished data).
(24 KB PDF)

Figure S23 Occurrence of Human Families and Fold Class SCOP folds were divided into a number of bins according to the number of families that they contain (*x*-axis). For each bin, the mean family occurrence (the mean number of human proteins in which families in the SCOP folds appear) is shown, divided among five SCOP fold classes. In general, as the number of families in a SCOP fold increases, the occurrence of these families in the human proteome tends to increase. A notable exception to this trend is shown for folds classified as Other. In this class, the C2H2, Ring finger, and the Rhodopsin-like families occupy over 500, 400, and 300 proteins, respectively, but belong to folds with only two to three known families.
(26 KB PDF)

Figure S24 Occurrence of Folds and Families across Genomes SCOP folds and families were searched for (see Materials and Methods) in proteins predicted to occur in the following genomes: Bacteroides fragilis NCTC9434, *Mus musculus, Deinococcus geothermalis, Escherichia coli* K12, Vibrio fischeri ES114, Psychrobacter arcticum 273 4, *Chlorobium vibrioforme, Sulfolobus acidocaldarius* DSM, *Saccharomyces cerevisiae, Mycoplasma hyopneumoniae* 232, *Streptococcus zooepidemicus,* and *Anabaena variabilis.* Folds were divided into a number of bins according to the number of families that they contain (*x*-axis). The mean number of organisms the folds (white bar) or families (black bar) appear in is plotted for each bin. Folds containing greater number of families tend to occur more often across organisms. Mean genome occurrences between folds with one and more than family were significantly different (MW-test, KS-test: *p* < 0.01). However, individual families belonging to folds with greater number of families tend not to occur more often across these organisms.
(23 KB PDF)

Protocol S1 Top Ten Fold Occurrence Differences between Organism Pairs (11 KB DOC)

Protocol S2 Disease Proteins Containing Folds with One Family (16 KB DOC)

Protocol S3 List of Common Disease Proteins (54 KB DOC)

Protocol S4 List of Rare Disease Proteins (15 KB DOC)

Protocol S5 Trend Exceptions For more information on the occurrence of human families and fold class, see Figure S23; for more information on folds and families across genomes, see Figure S24.
(65 KB DOC)

Combined Supporting Information All of the supporting material is combined into one file. (494 KB DOC)

### Accession Numbers

The Ensembl database (http://www.ensembl.org) ID numbers for the genes discussed in this paper are *GBA-*associated peptide (ensp00000314508), *G6PD* (ensp00000342362), *OPN1LW* (ensp00000218195), and *OPNL1MW* [*OPN1MW*] (ensp00000276343).

## Abbreviations

KS-test: Kolmogorov-Smirnov test
MW-test: Mann-Whitney test
SCOP: Structural Classification of Proteins
