# Supplementary material for: Fold Designability, Distribution, and Disease
Source: PLoS Comput Biol. 2006 May 5;2(5):e40. doi: 10.1371/journal.pcbi.0020040 (PMC1456317; doi:10.1371/journal.pcbi.0020040)
Supplement: Supplementary file 1 [file pcbi.0020040.sg001.pdf]

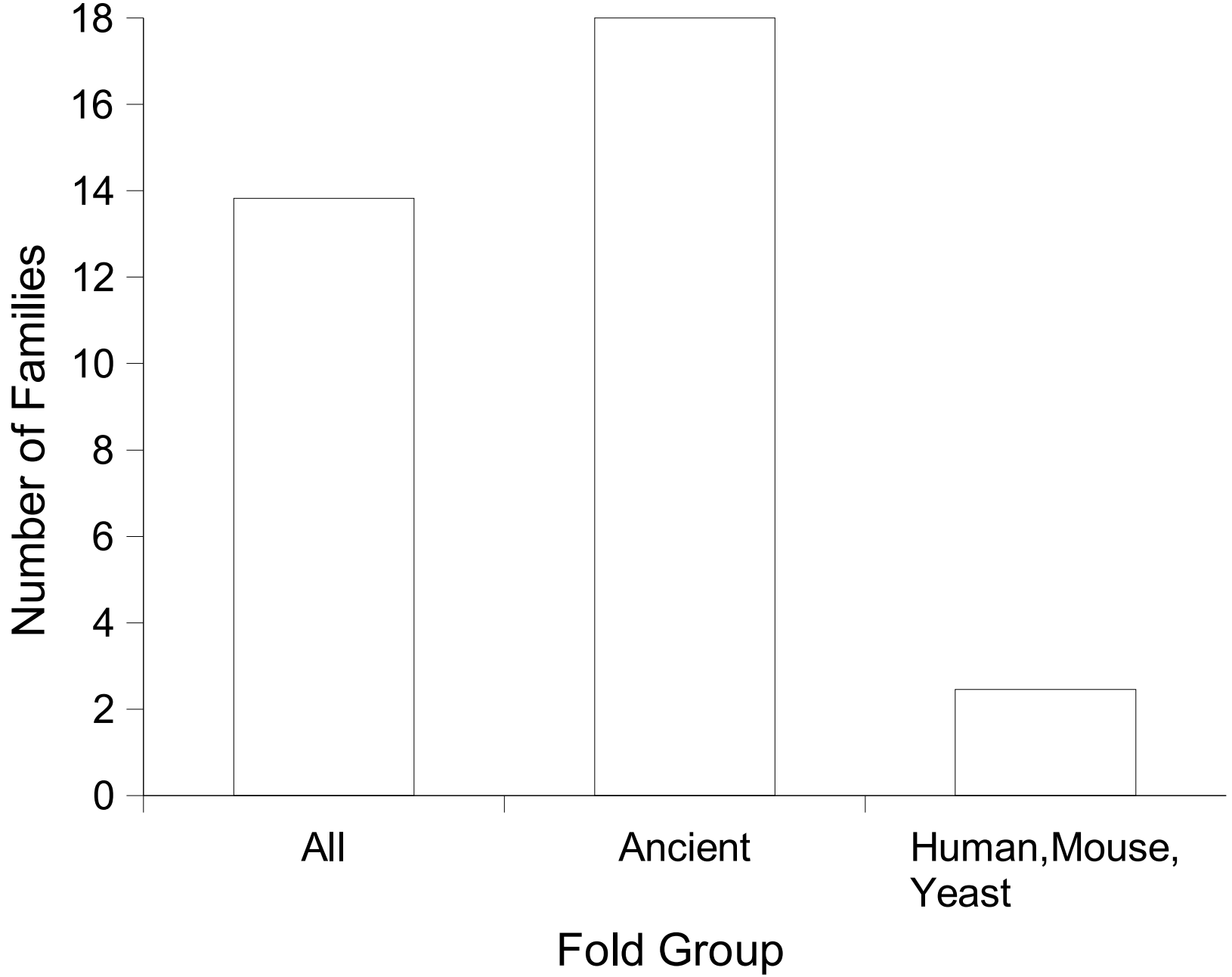

Number of Families
0
2
4
6
8
10
12
14
16
18
All
Ancient
Human,Mouse,
Yeast
Fold Group
