# Supplementary material for: Fold Designability, Distribution, and Disease
Source: PLoS Comput Biol. 2006 May 5;2(5):e40. doi: 10.1371/journal.pcbi.0020040 (PMC1456317; doi:10.1371/journal.pcbi.0020040)
Supplement: Supplementary file 2 [file pcbi.0020040.sg002.pdf]

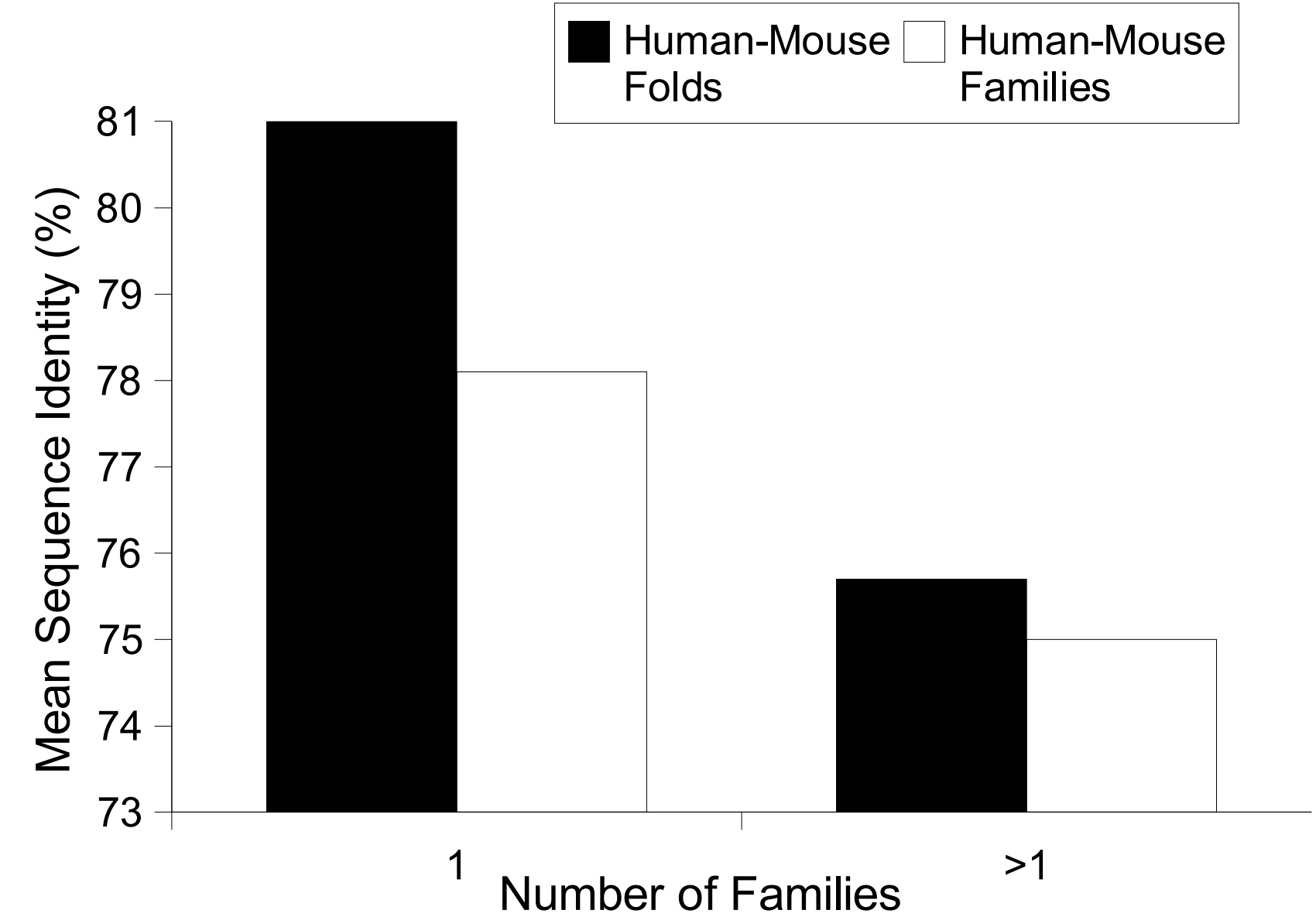
Human-Mouse Folds
Human-Mouse Families
81
80
79
78
77
76
75
74
73
Mean Sequence Identity (%)
1
>1
Number of Families
