# Supplementary material for: Fold Designability, Distribution, and Disease
Source: PLoS Comput Biol. 2006 May 5;2(5):e40. doi: 10.1371/journal.pcbi.0020040 (PMC1456317; doi:10.1371/journal.pcbi.0020040)
Supplement: Supplementary file 3 [file pcbi.0020040.sg003.pdf]

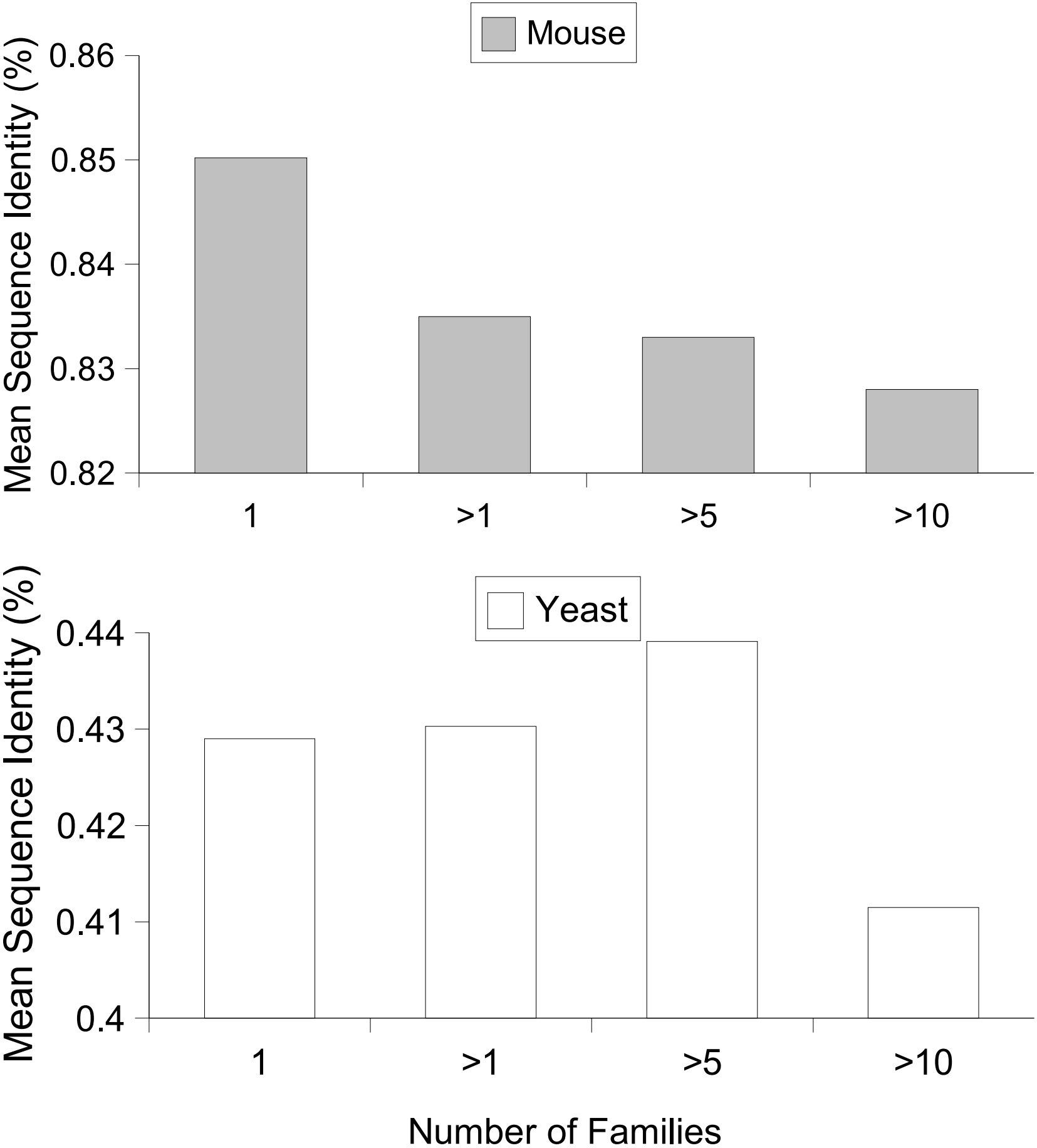

Mouse
Mean Sequence Identity (%)
0.86
0.85
0.84
0.83
0.82
1
>1
>5
>10
Yeast
Mean Sequence Identity (%)
0.44
0.43
0.42
0.41
0.4
1
>1
>5
>10
Number of Families
