# Supplementary material for: Fold Designability, Distribution, and Disease
Source: PLoS Comput Biol. 2006 May 5;2(5):e40. doi: 10.1371/journal.pcbi.0020040 (PMC1456317; doi:10.1371/journal.pcbi.0020040)
Supplement: Supplementary file 6 [file pcbi.0020040.sg006.pdf]

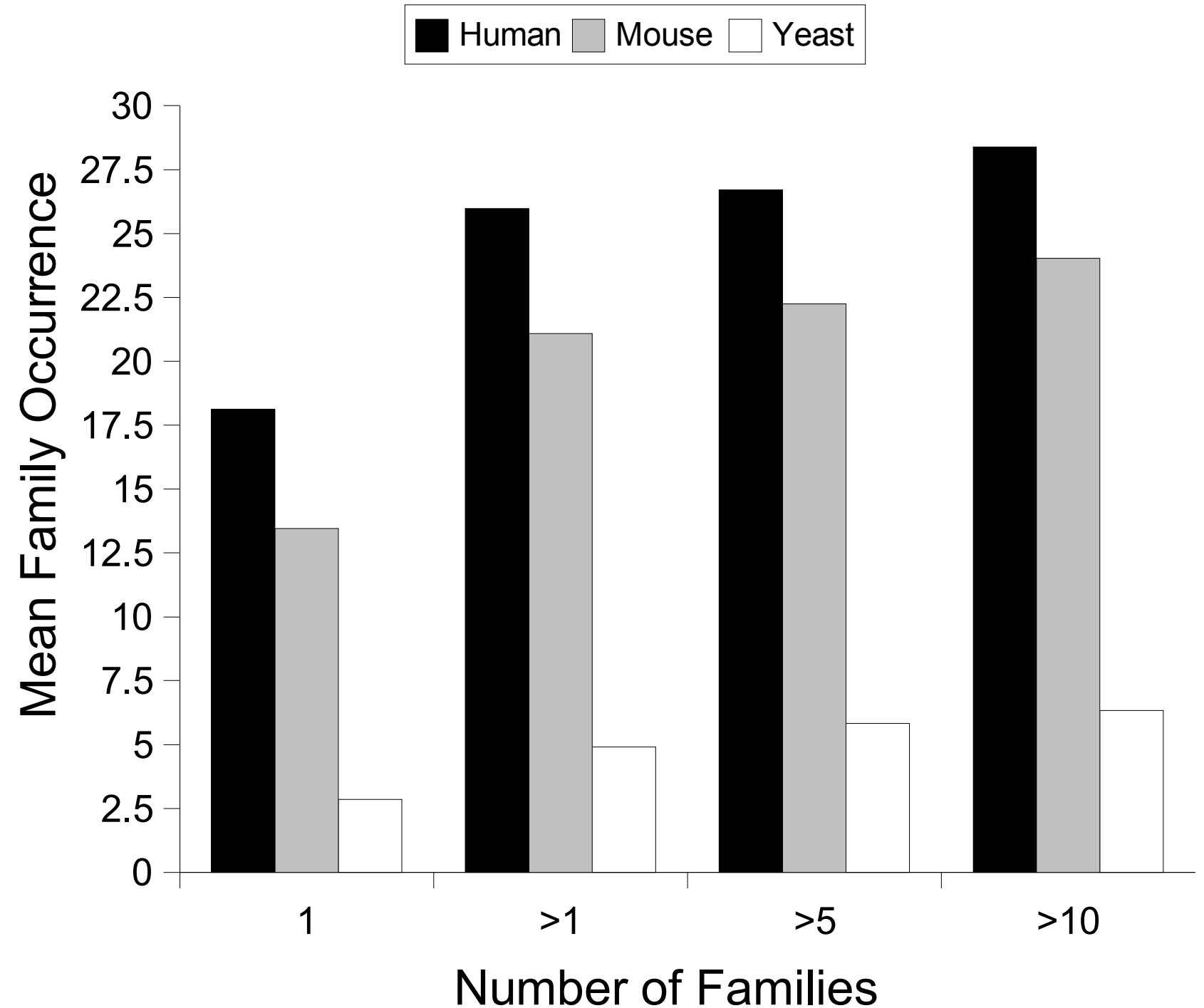
Human
Mouse
Yeast
30
27.5
25
22.5
20
17.5
15
12.5
10
7.5
5
2.5
0
Mean Family Occurrence
1
>1
>5
>10
Number of Families
