# Supplementary material for: Fold Designability, Distribution, and Disease
Source: PLoS Comput Biol. 2006 May 5;2(5):e40. doi: 10.1371/journal.pcbi.0020040 (PMC1456317; doi:10.1371/journal.pcbi.0020040)
Supplement: Supplementary file 11 [file pcbi.0020040.sg011.pdf]

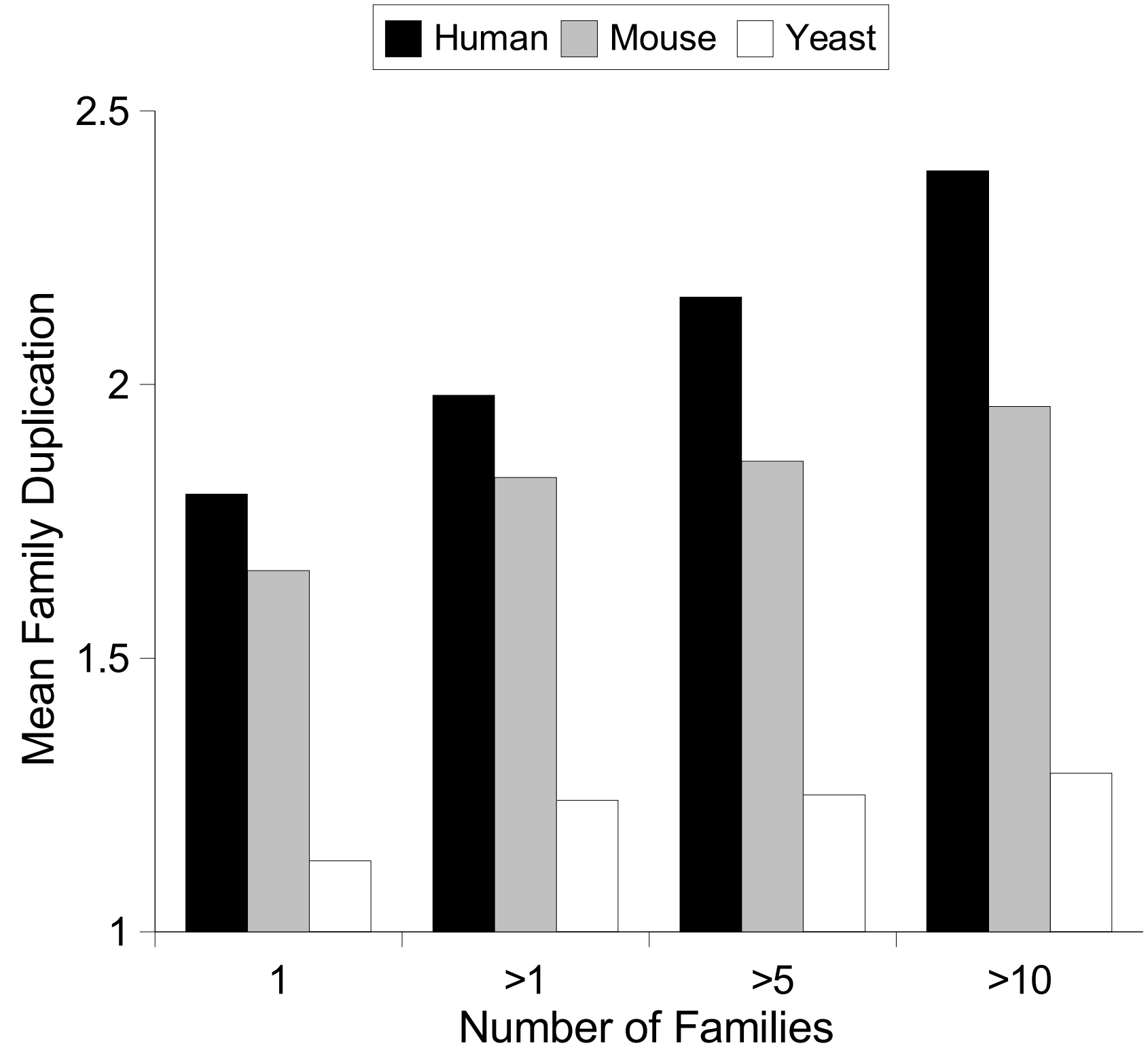
Human
Mouse
Yeast
2.5
2
1.5
1
Mean Family Duplication
1
>1
>5
>10
Number of Families
