# Supplementary material for: Fold Designability, Distribution, and Disease
Source: PLoS Comput Biol. 2006 May 5;2(5):e40. doi: 10.1371/journal.pcbi.0020040 (PMC1456317; doi:10.1371/journal.pcbi.0020040)
Supplement: Supplementary file 12 [file pcbi.0020040.sg012.pdf]

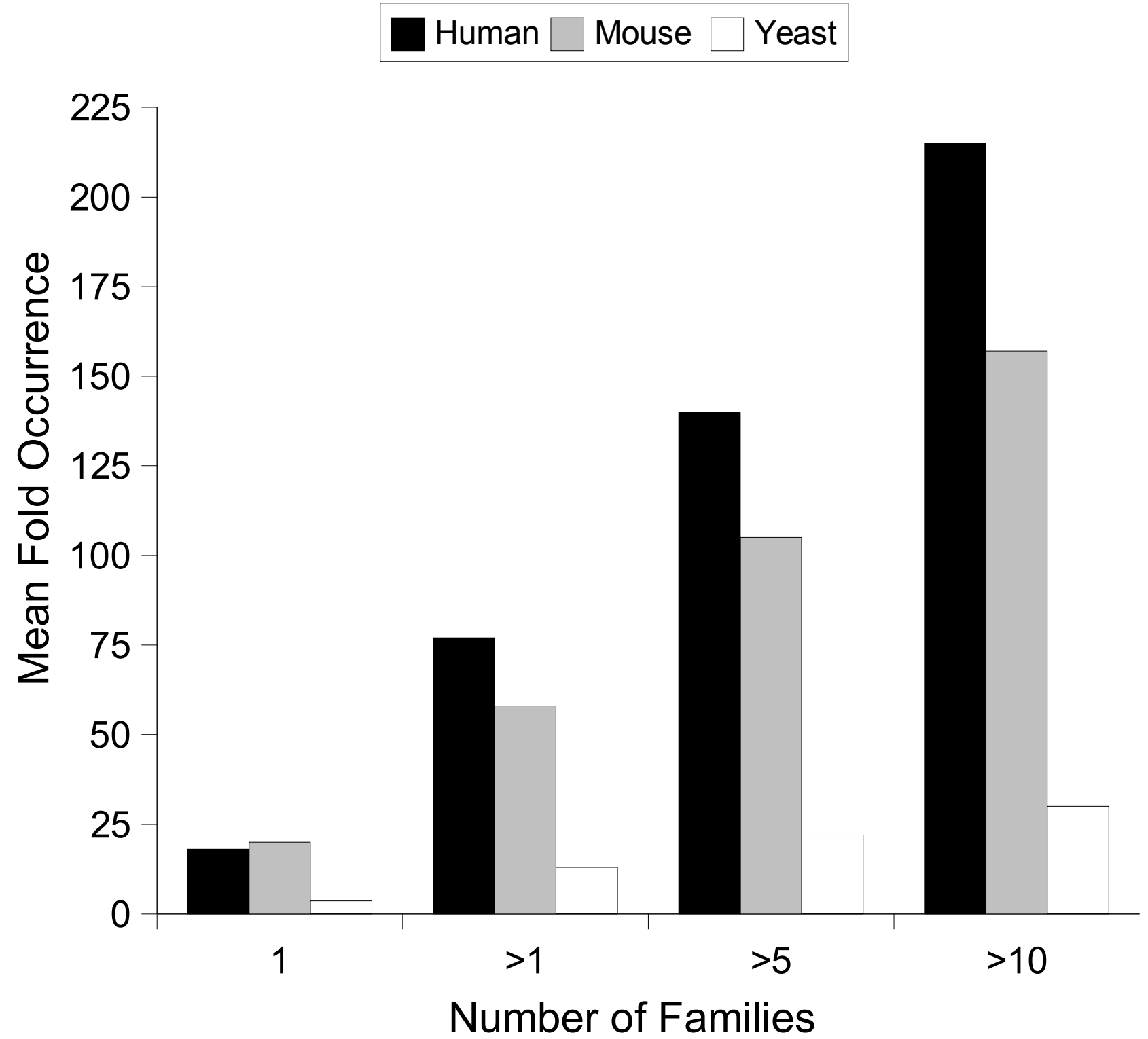

Human
Mouse
Yeast
Mean Fold Occurrence
225
200
175
150
125
100
75
50
25
0
1
>1
>5
>10
Number of Families
