# Supplementary material for: Fold Designability, Distribution, and Disease
Source: PLoS Comput Biol. 2006 May 5;2(5):e40. doi: 10.1371/journal.pcbi.0020040 (PMC1456317; doi:10.1371/journal.pcbi.0020040)
Supplement: Supplementary file 13 [file pcbi.0020040.sg013.pdf]

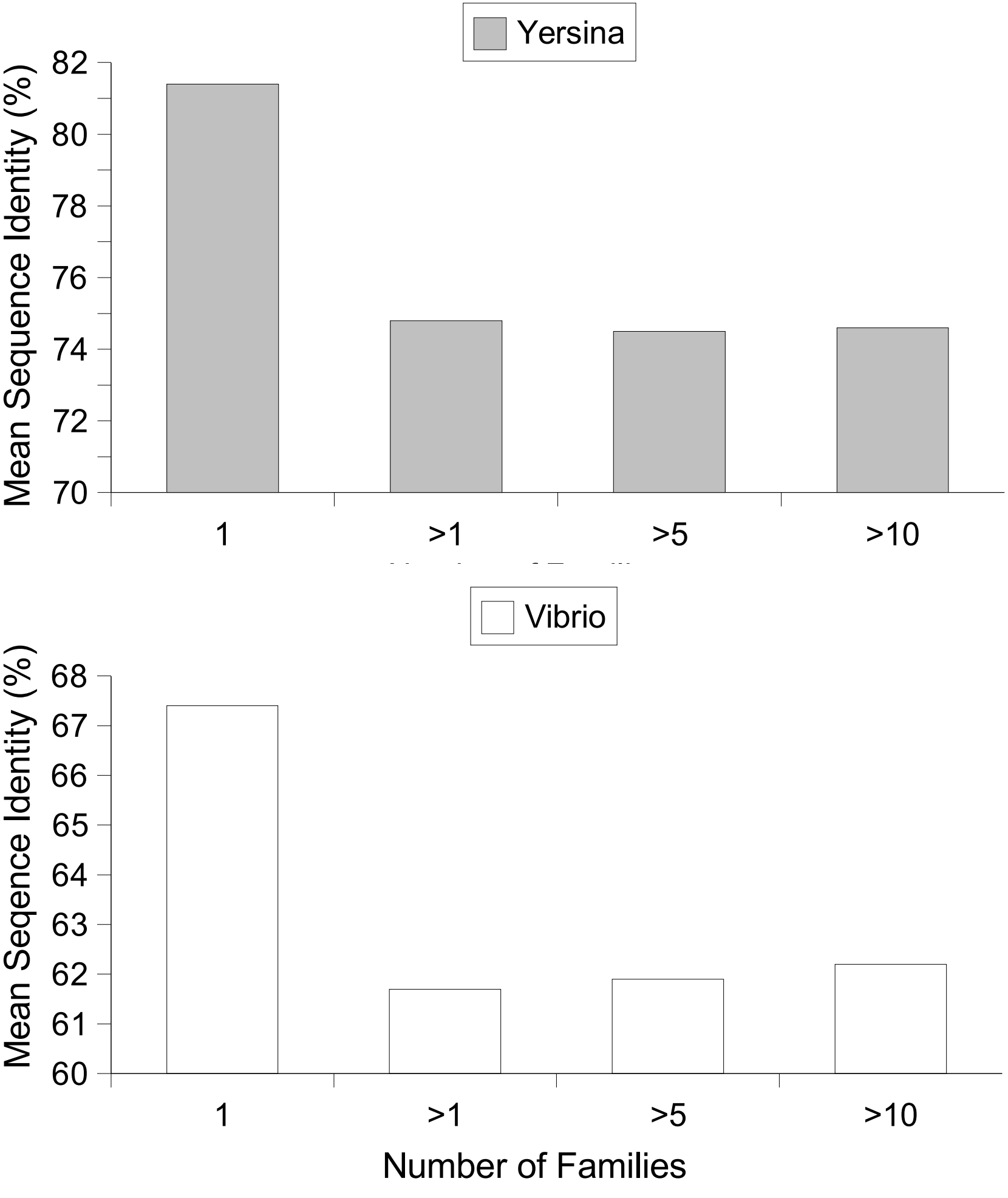
Yersina
Mean Sequence Identity (%)
82
80
78
76
74
72
70
1
>1
>5
>10
Vibrio
Mean Segence Identity (%)
68
67
66
65
64
63
62
61
60
1
>1
>5
>10
Number of Families
