# Supplementary material for: Fold Designability, Distribution, and Disease
Source: PLoS Comput Biol. 2006 May 5;2(5):e40. doi: 10.1371/journal.pcbi.0020040 (PMC1456317; doi:10.1371/journal.pcbi.0020040)
Supplement: Supplementary file 14 [file pcbi.0020040.sg014.pdf]

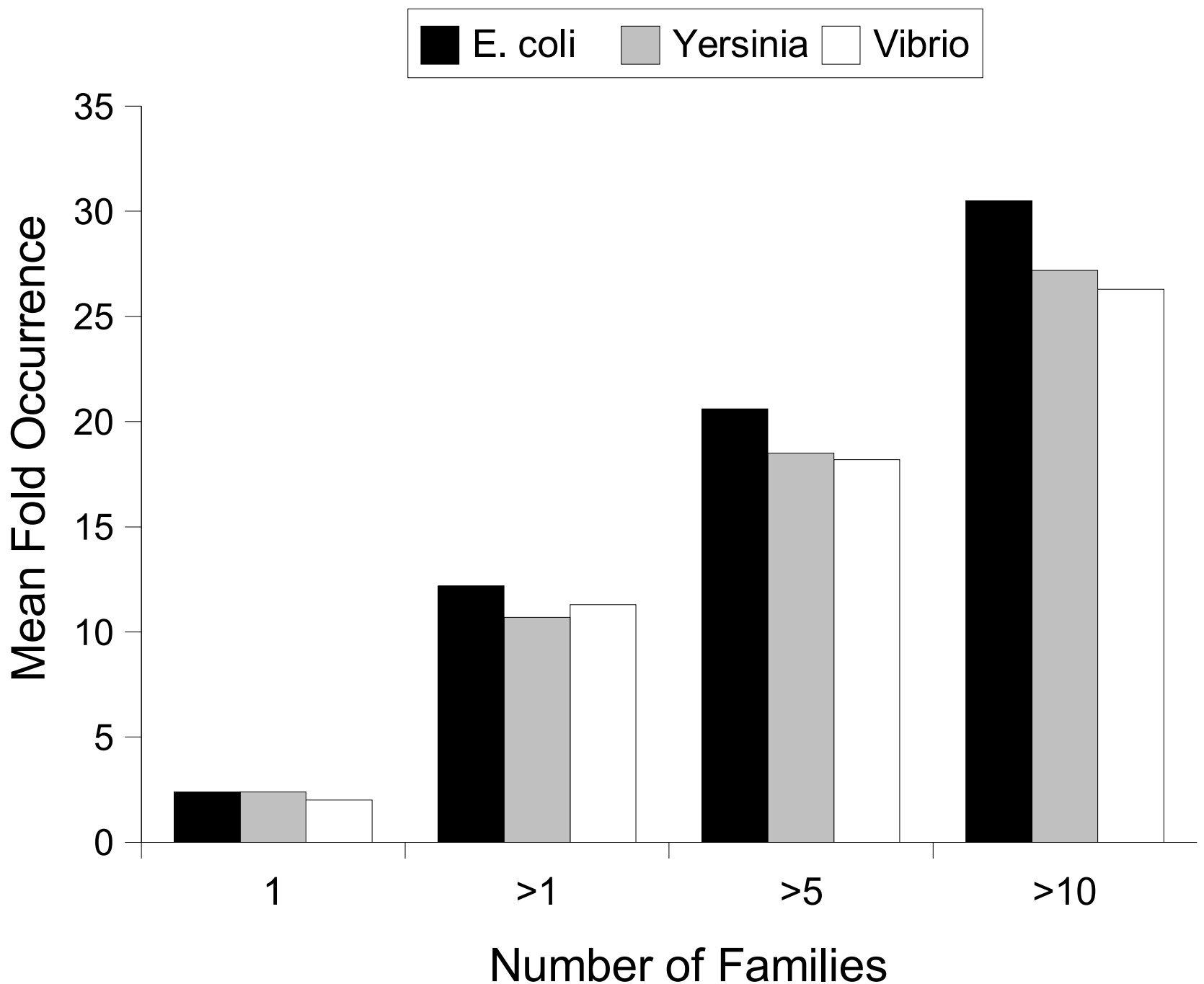

E. coli
Yersinia
Vibrio
35
30
25
20
15
10
5
0
Mean Fold Occurrence
1
>1
>5
>10
Number of Families
