# Supplementary material for: Fold Designability, Distribution, and Disease
Source: PLoS Comput Biol. 2006 May 5;2(5):e40. doi: 10.1371/journal.pcbi.0020040 (PMC1456317; doi:10.1371/journal.pcbi.0020040)
Supplement: Supplementary file 15 [file pcbi.0020040.sg015.pdf]

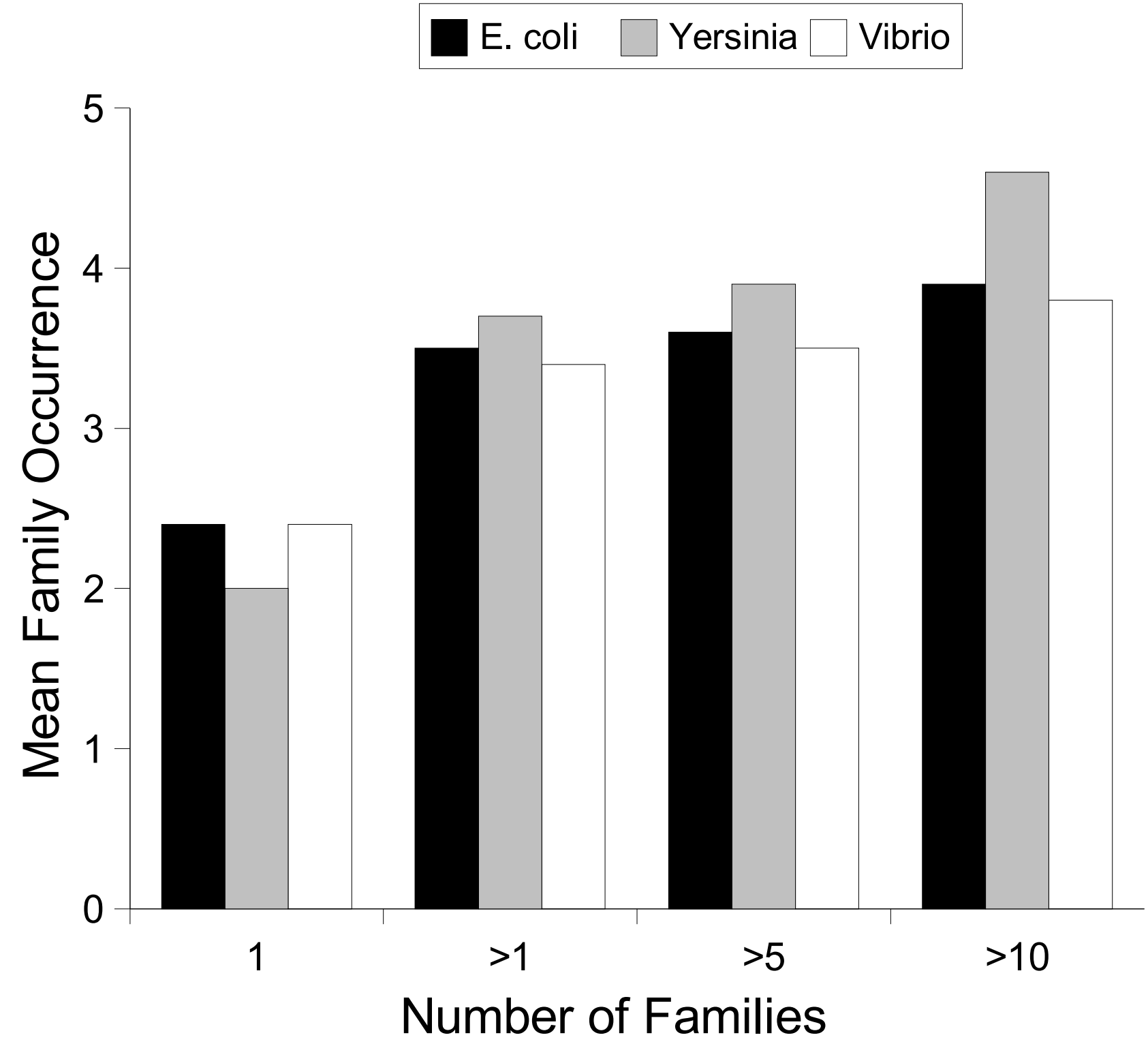
E. coli
Yersinia
Vibrio
Mean Family Occurrence
0
1
2
3
4
5
1
>1
>5
>10
Number of Families
