# Supplementary material for: Fold Designability, Distribution, and Disease
Source: PLoS Comput Biol. 2006 May 5;2(5):e40. doi: 10.1371/journal.pcbi.0020040 (PMC1456317; doi:10.1371/journal.pcbi.0020040)
Supplement: Supplementary file 18 [file pcbi.0020040.sg018.pdf]

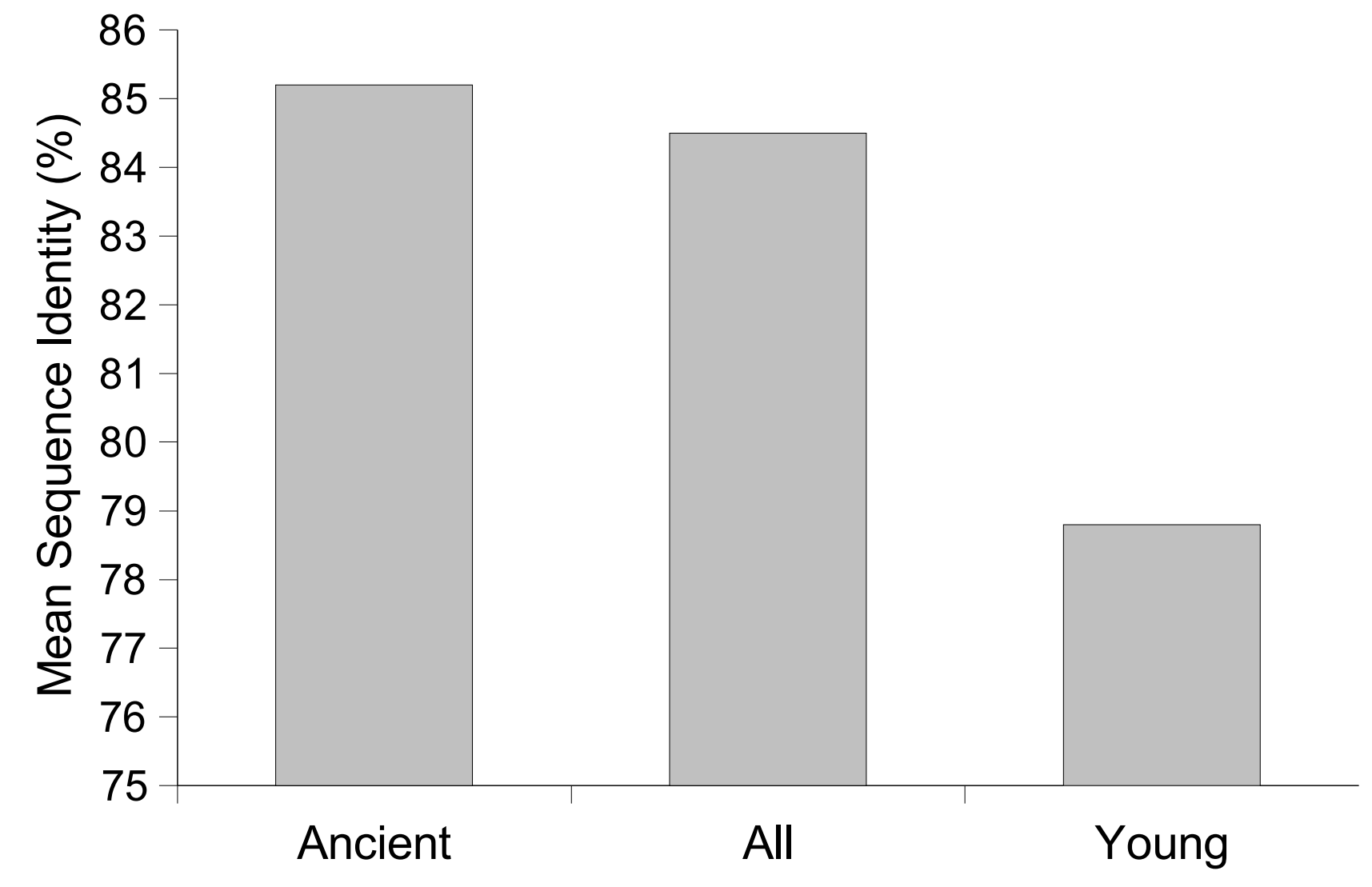

Mean Sequence Identity (%)
86
85
84
83
82
81
80
79
78
77
76
75
Ancient
All
Young
