# Supplementary material for: Fold Designability, Distribution, and Disease
Source: PLoS Comput Biol. 2006 May 5;2(5):e40. doi: 10.1371/journal.pcbi.0020040 (PMC1456317; doi:10.1371/journal.pcbi.0020040)
Supplement: Supplementary file 19 [file pcbi.0020040.sg019.pdf]

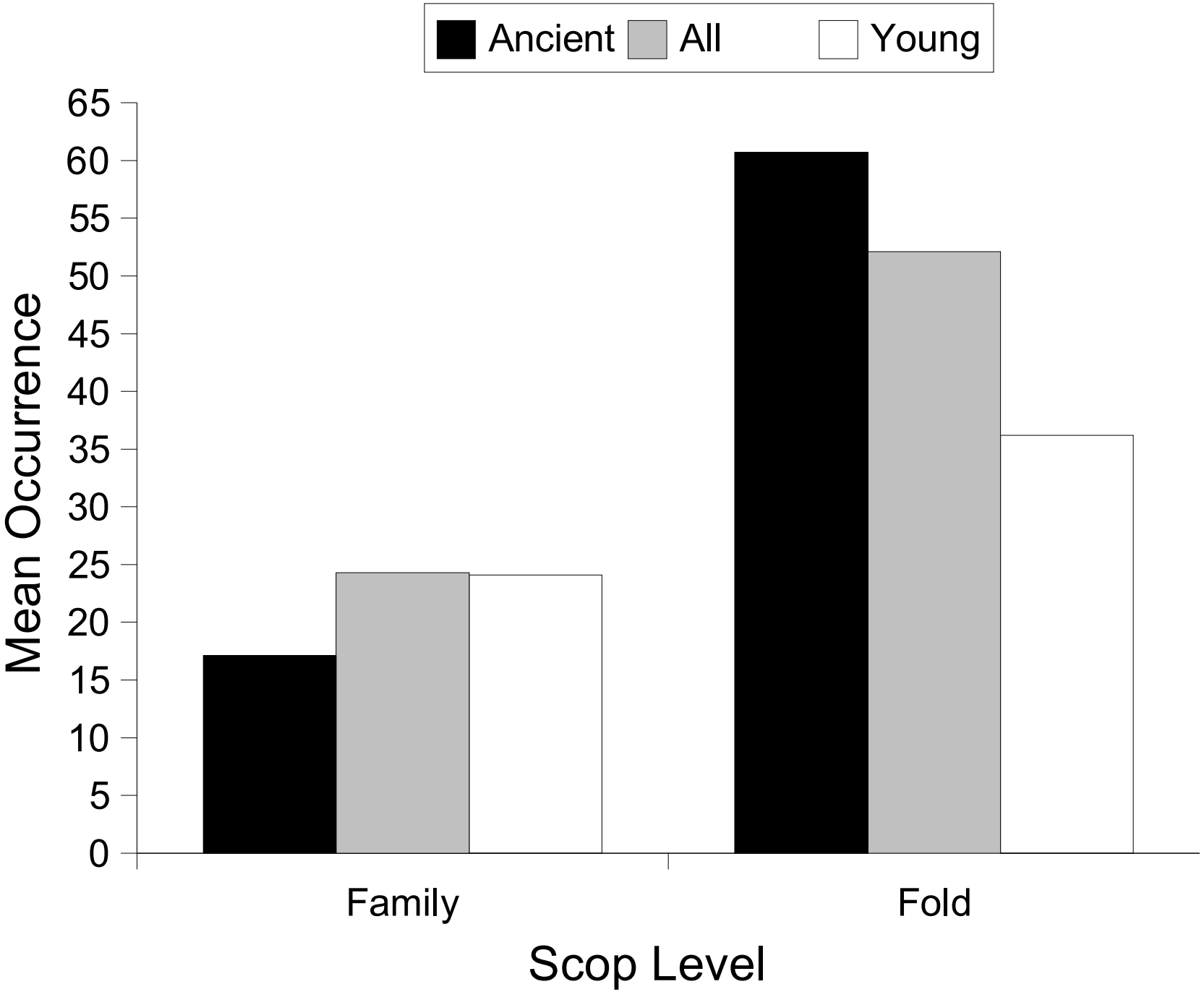
Ancient
All
Young
65
60
55
50
45
40
35
30
25
20
15
10
5
0
Mean Occurrence
Family
Fold
Scop Level
