# Supplementary material for: Fold Designability, Distribution, and Disease
Source: PLoS Comput Biol. 2006 May 5;2(5):e40. doi: 10.1371/journal.pcbi.0020040 (PMC1456317; doi:10.1371/journal.pcbi.0020040)
Supplement: Supplementary file 21 [file pcbi.0020040.sg021.pdf]

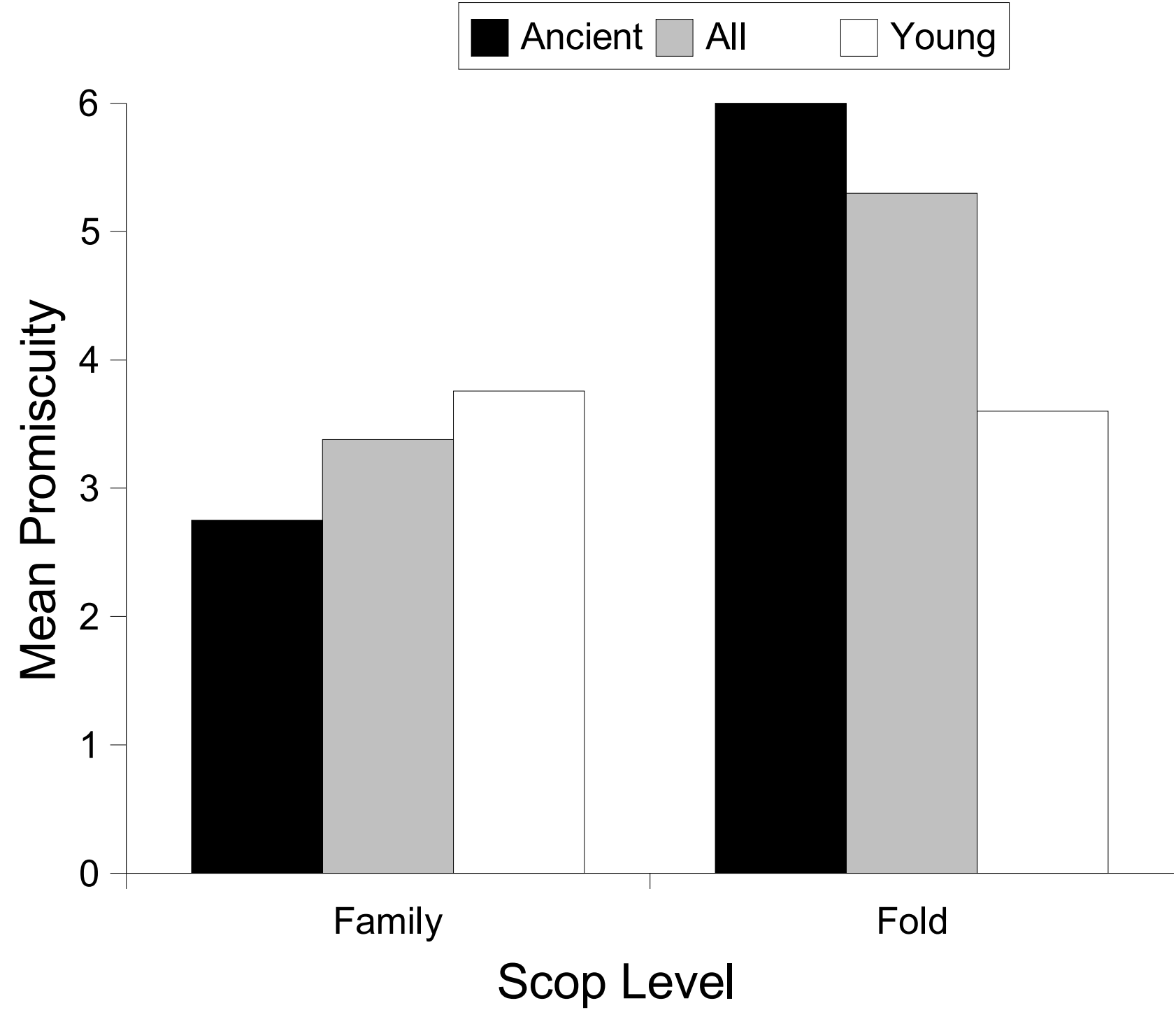

Ancient
All
Young
Mean Promiscuity
0
1
2
3
4
5
6
Family
Fold
Scop Level
