# Supplementary material for: Fold Designability, Distribution, and Disease
Source: PLoS Comput Biol. 2006 May 5;2(5):e40. doi: 10.1371/journal.pcbi.0020040 (PMC1456317; doi:10.1371/journal.pcbi.0020040)
Supplement: Supplementary file 22 [file pcbi.0020040.sg022.pdf]

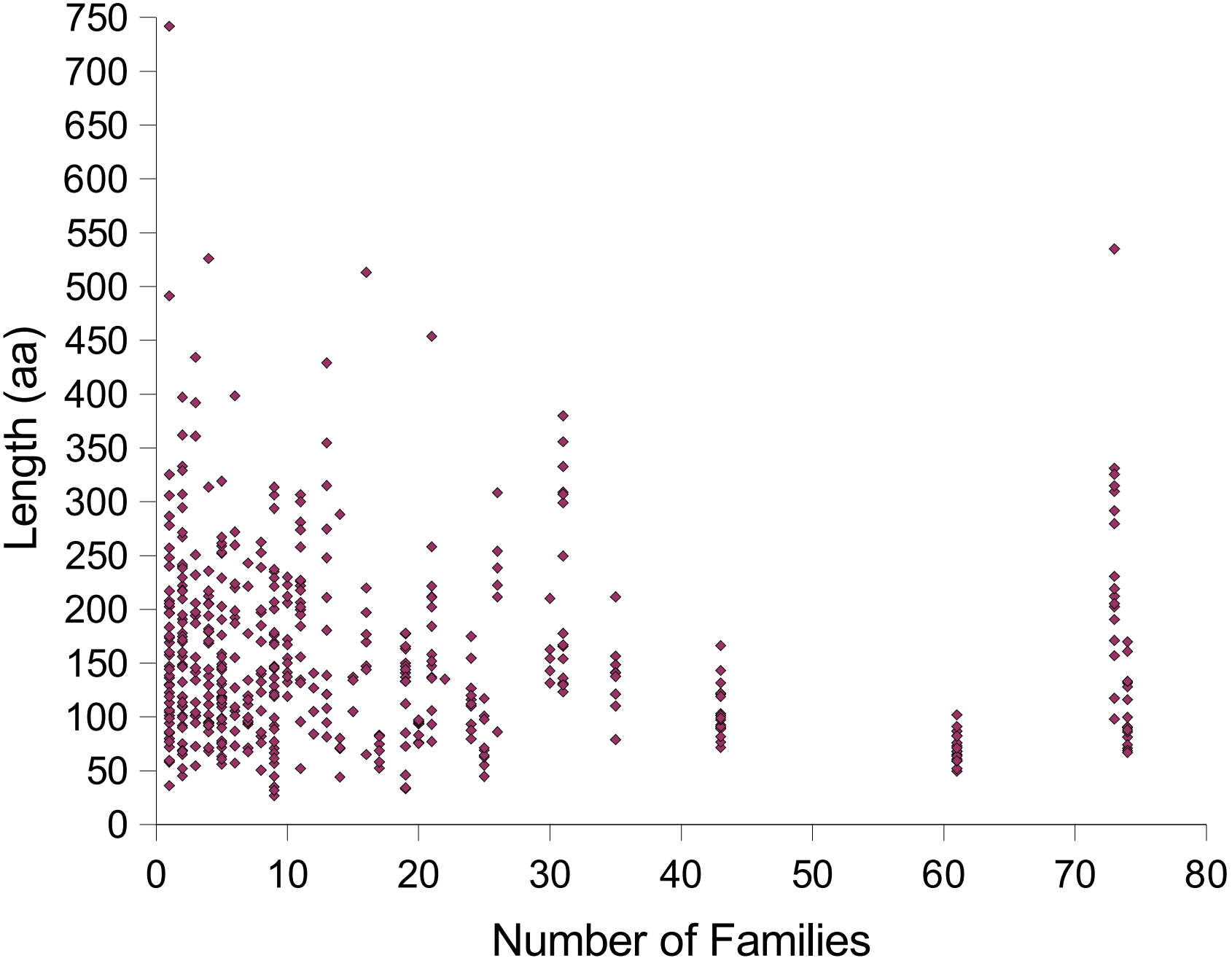

750
700
650
600
550
500
450
400
350
300
250
200
150
100
50
0
Length (aa)
0
10
20
30
40
50
60
70
80
Number of Families
