# Supplementary material for: Fold Designability, Distribution, and Disease
Source: PLoS Comput Biol. 2006 May 5;2(5):e40. doi: 10.1371/journal.pcbi.0020040 (PMC1456317; doi:10.1371/journal.pcbi.0020040)
Supplement: Supplementary file 23 [file pcbi.0020040.sg023.pdf]

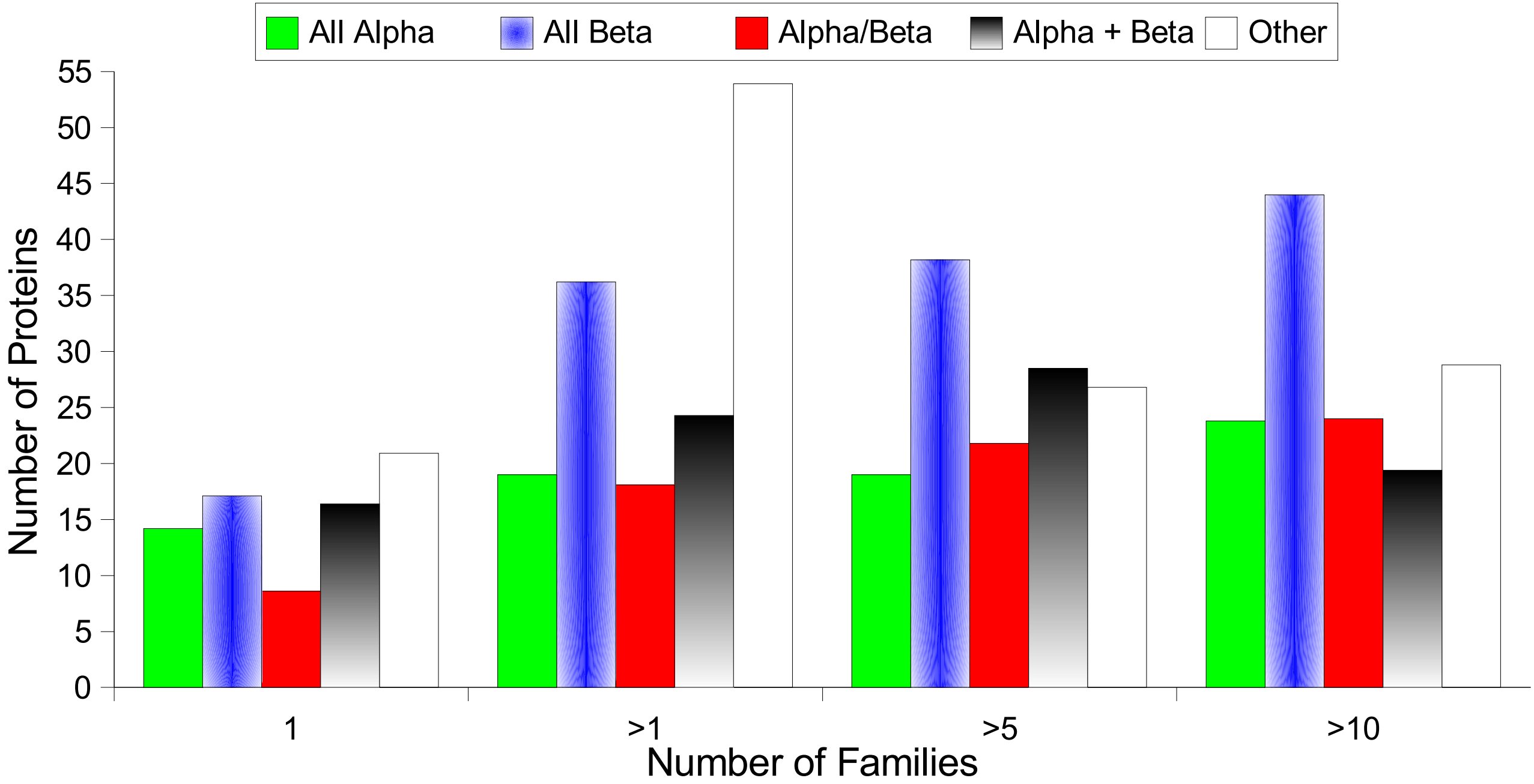
All Alpha
All Beta
Alpha/Beta
Alpha + Beta
Other
Number of Proteins
0
5
10
15
20
25
30
35
40
45
50
55
1
>1
>5
>10
Number of Families
