# Supplementary material for: Fold Designability, Distribution, and Disease
Source: PLoS Comput Biol. 2006 May 5;2(5):e40. doi: 10.1371/journal.pcbi.0020040 (PMC1456317; doi:10.1371/journal.pcbi.0020040)
Supplement: Supplementary file 25 [file pcbi.0020040.sd001.doc]

|  | yeast - ecoli | mouse - ecoli | human - ecoli | mouse - yeast | human - yeast | human - mouse |
| --- | --- | --- | --- | --- | --- | --- |
| 1 | c.37 | b.1 | b.1 | b.1 | b.1 | b.1 |
| 2 | a.118 | c.37 | c.37 | c.37 | d.144 | d.144 |
| 3 | b.69 | a.4 | a.4 | d.144 | c.37 | c.37 |
| 4 | b.34 | d.58 | b.34 | a.4 | a.4 | g.37 |
| 5 | d.153 | c.2 | b.69 | c.2 | d.58 | b.34 |
| 6 | g.41 | b.69 | d.58 | d.58 | b.34 | a.4 |
| 7 | c.55 | a.118 | a.118 | c.10 | d.211 | b.55 |
| 8 | b.43 | d.81 | b.36 | d.81 | c.10 | b.69 |
| 9 | b.80 | b.34 | b.47 | d.211 | b.69 | d.211 |
| 10 | a.2 | b.47 | b.7 | b.69 | b.55 | g.3 |

**Protocol S1: Top Ten Fold Occurrence Differences between Organism Pairs**

The 10 folds with the largest fold occurrence differences for each organism pair are listed in each column. Comparing the last 3 columns: fold occurrence differences between yeast and the two mammals is similar to fold occurrence differences between human and mouse. Comparing the first 3 columns: fold occurrence differences between *E. coli* and each of the three eukaryotes is less similar. The c.37 (P-loop containing nucleoside triphosphate hydrolases) and the b.69 (7-bladed beta-propeller) fold have consistently exhibited large occurrence differences between organism pairs. Other notable folds include b.1, d.58, and a.4, all of which are predicted to be highly designable.
