# Supplementary material for: Fold Designability, Distribution, and Disease
Source: PLoS Comput Biol. 2006 May 5;2(5):e40. doi: 10.1371/journal.pcbi.0020040 (PMC1456317; doi:10.1371/journal.pcbi.0020040)
Supplement: Supplementary file 26 [file pcbi.0020040.sd002.doc]

**Protocol S2: Disease Proteins containing folds with 1 family**

In this and previous work [7], it was found that disease proteins have significantly higher number of proteins containing folds with 1 family as compared with non-disease proteins. We list these proteins here, along with their folds and associated diseases as annotated in OMIM. The list contains 228 proteins and 106 different folds associated with a diversity of diseases. 15 and 10 proteins respectively are annotated with complement system and leukemia related diseases and form the largest grouping of proteins related by disease annotation. Even after removing these proteins, disease proteins were predicted to be significantly less designable than non-disease proteins by their fold family counts (MW-test: *p* < 0.05, KS-test: *p* < 0.01; data not shown). An * in the fold column indicates that the SCOP definition highly covers the protein (see Methods). The folds below were also scanned across genomes (Supplementary Material S24). Most of the folds (~2/3) were detected only in human, mouse and/or yeast suggesting that they are eukaryotic in origin. However, (~1/3) were detected in 3 other prokaryotes and (~1/4) of the folds below present in over 7 genomes (see Methods). These latter folds are relatively ancient and the absence of many families in these folds suggests that they are relatively less designable. Disease proteins were predicted to be significantly less designable than non-disease proteins by their fold family counts even if we excluded the young folds predicted to be eukaryotic in origin (MW-test: *p* < 0.1, KS-test: *p* < 0.01; data not shown).

ENSEMBL Protein	Folds	Associated OMIM Disease Annotation

ensp00000226359	a.126	[AFP deficiency congenital] (1),[Hereditary persistence of alpha-fetoprotein] (3)ensp00000261779	g.18	[Anhaptoglobinemia] (3),[Hypohaptogloginemia] (3)

ensp00000205948	g.18,	[Apolipoprotein H deficiency] (3)

ensp00000200676	d.83	[CETP deficiency] (3)

ensp00000329374	e.1*	[Euthyroidal hyper- and hypothyroxinemia] (1)

ensp00000342850	e.1*	[Transcortin deficiency] (1)

ensp00000289416	e.23	{?Hypertension essential} (1)

ensp00000224541	d.9	{AIDS resistance to} (3)

ensp00000331736	g.18,	{Atherosclerosis susceptibility to} (2)

ensp00000302692	d.19*	{Beryllium disease chronic susceptibility to} (3)

ensp00000234420	a.113	{Cancer susceptibility} (3)

ensp00000258224	e.1	{Hypertension essential susceptibility to} (3),{Preeclampsia susceptibility to} (3)

ensp00000313875	g.18,	{Measles susceptibility to} (1)

ensp00000335243	d.19*	{Pemphigoid susceptibility to} (2),Diabetes mellitus insulin-dependent-1 (2) (?)

ensp00000304123	b.22	{Systemic lupus erythematosus susceptibility} 152700 (3)

ensp00000218425	a.87	Aarskog-Scott syndrome (3)

ensp00000265517	f.7	Abetalipoproteinemia 200100 (3)

ensp00000233242	f.7	Abetalipoproteinemia (3),Apolipoprotein B-100 ligand-defective (3),

Hyperbetalipoproteinemia (3),Hypobetalipoproteinemia (3)

ensp00000241052	e.5*	Acatalasemia (3)

ensp00000292427	a.104	Adrenal hyperplasia congenital due to 11-beta-hydroxylase deficiency (3)

Aldosteronism glucocorticoid-remediable (3)

ensp00000278017	a.104*	Adrenal hyperplasia congenital due to 17-alpha-hydroxylase deficiency (3)

ensp00000303875	a.104*	Adrenal hyperplasia congenital due to 21-hydroxylase deficiency (3)

Carbamoylphosphate synthetase I deficiency (3)

ensp00000302390	a.123	Adrenal hypoplasia congenital with hypogonadotropic hypogonadism (3)

ensp00000311469	a.45	Agammaglobulinemia 601495 (3)

ensp00000337233	d.93	Agammaglobulinemia type 1 X-linked (3)

XLA and isolated growth hormone deficiency 307200 (3) (?)

ensp00000261981	e.1*	Alpha-1-antichymotrypsin deficiency (3),Cerebrovascular disease occlusive (3)

ensp00000306361	d.171	Amyloidosis hereditary renal 105200 (3)

Dysfibrinogenemia alpha type causing bleeding diathesis (3)

Dysfibrinogenemia alpha type causing recurrent thrombosis (3)

ensp00000295897	a.126	Analbuminemia (3),[Dysalbuminemic hyperthyroxinemia] (3)

[Dysalbuminemic hyperzincemia] 194470 (3)

ensp00000313925	a.123	Androgen insensitivity several forms (3),Breast cancer male with Reifenstein syndrome (3),	Perineal hypospadias (3),Prostate cancer (3),

Spinal and bulbar muscular atrophy of Kennedy 313200 (3)

ensp00000271945	b.58	Anemia hemolytic due to PK deficiency (3)

ensp00000343033	c.71*	Anemia megaloblastic due to DHFR deficiency (1) (?)

ensp00000345138	c.71	Anemia megaloblastic due to DHFR deficiency (1) (?)

ensp00000200691	g.46*	Anemia pernicious congenital due to deficiency of intrinsic factor (1)

ensp00000232165	d.148	Angelman syndrome (3),Ankylosing spondylitis (2)

ensp00000278407	e.1	Angioedema hereditary (3)

ensp00000289824	b.22	Anhidrotic ectodermal dysplasia (2)

ensp00000296507	a.38	Anhidrotic ectodermal dysplasia (2),Ectodermal dysplasia hidrotic (2)

ensp00000236260	e.1*	Antithrombin III deficiency (3)

ensp00000327433	f.37	Bare lymphocyte syndrome type I due to TAP2 deficiency (1)

ensp00000274376	d.93	Basal cell carcinoma (3)

ensp00000286548	a.66	Bleeding diathesis due to GNAQ deficiency (1)

ensp00000242261	a.38	Blepharophimosis epicanthus inversus and ptosis type 2 (2),

Saethre-Chotzen syndrome 101400 (3)

ensp00000206249	a.123	Breast cancer (1),Estrogen resistance (3)

ensp00000267071	a.170,a.171	Breast cancer 2 early onset (3),Pancreatic cancer (3),Fanconi anemia type D (2)

ensp00000315969	f.33,d.220	Brody myopathy 601003 (3)

ensp00000259523	a.38	Burkitt lymphoma (3)

ensp00000313967	b.22	C1q deficiency type B (3)

ensp00000290301	b.22	C1q deficiency type C (3)

ensp00000290575	g.18	C1r/C1s deficiency combined (1)

ensp00000328173	g.18	C1r/C1s deficiency combined (1)

ensp00000299367	g.18	C2 deficiency (3)

ensp00000245907	a.50	C3 deficiency (3)

ensp00000265172	g.12	C3b inactivator deficiency (3)

ensp00000299406	a.50	C4 deficiency (3)

ensp00000223642	a.50	C5 deficiency (1)

ensp00000338861	g.60,g.12	C6 deficiency (1),Combined C6/C7 deficiency (1)

ensp00000322061	g.60,g.18,g.12	C7 deficiency (1)

ensp00000305651	d.9*	C7 deficiency (1)

ensp00000287797	g.60,g.12	C8 deficiency type I (2)

ensp00000234643	g.60,g.12	C8 deficiency type II (3)

ensp00000263408	g.12	C9 deficiency (3)

ensp00000245479	a.21	Campomelic dysplasia with autosomal sex reversal (3),Cardiomyopathy familial dilated 1B (2)

ensp00000233072	a.92	Carbamoylphosphate synthetase I deficiency (3)

ensp00000316712	d.93	Cardiofaciocutaneous syndrome 115150 (2),Noonan syndrome-1 (2)

ensp00000341922	a.173	Cataract cerulean type 1 (2)

ensp00000258415	a.104	Cerebrotendinous xanthomatosis (3)

ensp00000272000	a.169	Chediak-Higashi syndrome (3)

ensp00000265723	f.37	Cholestasis progressive familial intrahepatic type III 602347 (3)

ensp00000333122	a.123	Chondrosarcoma extraskeletal myxoid (1)

ensp00000289473	d.189	Chronic granulomatous disease due to deficiency of NCF-1 (3)

ensp00000342903	d.210	Citrullinemia (3)

ensp00000325822	a.104	CMO II deficiency (3)

ensp00000265724	f.37	Colchicine resistance (3)

ensp00000269305	a.53	Colorectal cancer 114500 (3),Li-Fraumeni syndrome (3)

ensp00000233146	a.113	Colorectal cancer hereditary nonpolyposis type 1 (3) Ovarian cancer (3),Muir-Torre syndrome 158320 (3)

ensp00000343888	a.21	Colorectal cancer hereditary nonpolyposis type 3 (3)

ensp00000311165	f.19	Colton blood group 110450 (3)

ensp00000251047	a.21	Combined factor V and VIII deficiency 227300 (3)

ensp00000181839	e.32	Congenital hereditary endothelial dystrophy of cornea (2)

ensp00000262464	g.23	Contractural arachnodactyly congenital (3)

ensp00000306306	b.118	Corneal dystrophy Avellino type (3),Corneal dystrophy Groenouw type I 121900 (3),

Corneal dystrophy lattice type I 122200 (3),Reis-Bucklers corneal dystrophy (3)

ensp00000305913	b.22	Corneal dystrophy posterior polymorphous (2)

ensp00000231509	a.123	Cortisol resistance (3)

ensp00000301141	a.104*	Coumarin resistance 122700 (3),{Nicotine addiction protection from} (3)

ensp00000263045	d.111*	Craniosynostosis Adelaide type (2)

ensp00000339996	d.6	Creutzfeldt-Jakob disease 123400 (3),Gerstmann-Straussler disease 137440 (3),

Insomnia fatal familial (3),5-oxoprolinuria 266130 (3),

Hemolytic anemia due to glutathione synthetase deficiency 231900 (3)

ensp00000222399	a.114	Cutis laxa marfanoid neonatal type (1) (?)

ensp00000231004	a.80	Cutis laxa recessive type I 219100 (1)

ensp00000256216	d.106	D-bifunctional protein deficiency (3)

ensp00000324892	f.33,d.220	Darier disease (keratosis follicularis) (2)

ensp00000329773	f.14	Deafness autosomal dominant 2 (2)

ensp00000216361	d.209	Deafness autosomal dominant 9 (2)

ensp00000291532	g.12	Deafness autosomal recessive 8 (2)

ensp00000248928	a.104*	Debrisoquine sensitivity (3),{?Parkinsonism susceptibility to} (1)

ensp00000223955	g.62	Dementia familial nonspecific (2)

ensp00000304257	f.20	Dent disease 300009 (3),Hypophosphatemia type III (3),Nephrolithiasis type I 310468 (3),

Proteinuria low molecular weight with hypercalciuric nephrocalcinosis (3)

ensp00000199280	f.19*	Diabetes insipidus nephrogenic autosomal recessive 222000 (3)

ensp00000217172	b.9*	Diabetes insipidus neurohypophyseal 125700 (3)

ensp00000250971	g.1*	Diabetes mellitus rare form (1),Hyperproinsulinemia familial (3),MODY one form (3)

ensp00000263196	g.12	DiGeorge syndrome/velocardiofacial syndrome complex-2 (2)

ensp00000266063	f.37	Dubin-Johnson syndrome 237500 (3)

ensp00000306099	d.171	Dysfibrinogenemia beta type (3)

ensp00000336829	d.171	Dysfibrinogenemia gamma type (3),Hypofibrinogenemia gamma type (3)

ensp00000308541	g.32	Dysprothrombinemia (3),Hypoprothrombinemia (3)

ensp00000325527	g.23	Ectopia lentis ?isolated (3),Marfan syndrome 154700 (3),

Shprintzen-Goldberg syndrome 182212 (3)

ensp00000323534	g.27	Ehlers-Danlos syndrome type X (1) (?)

ensp00000298844	e.1*	Emphysema (3),Emphysema-cirrhosis (3),

Hemorrhagic diathesis due to `antithrombin' Pittsburgh (3)

ensp00000265081	a.113	Endometrial carcinoma (3)

ensp00000234590	d.54	Enolase deficiency (1)

ensp00000284885	g.12	Enterokinase deficiency (1)

ensp00000342552	f.14	Epilepsy benign neonatal type 1 121200 (3),

Epilepsy benign neonatal type 1 121200 (3),Epilepsy nocturnal frontal lobe 600513 (3)

ensp00000217176	b.96,f.36	Epilepsy benign neonatal type 1 121200 (3),Epilepsy nocturnal frontal lobe 600513 (3),	Epilepsy benign neonatal type 1 121200 (3)

ensp00000330043	f.14	Epilepsy benign neonatal type 2 121201 (3)

ensp00000228858	f.14	Episodic ataxia/myokymia syndrome 160120 (3)

ensp00000001567	g.18,	Factor H deficiency (1),Hemolytic-uremic syndrome 235400 (3),

Membroproliferative glomerulonephritis (1)

ensp00000261967	g.32	Factor VII deficiency (3)

ensp00000246496	g.32	Factor X deficiency (3)

ensp00000271509	g.18,	Factor XIIIB deficiency (3)

ensp00000272146	a.41	Fanconi anemia (1) (?),Xeroderma pigmentosum (1) (?)

ensp00000263879	a.117,a.87	Fibromatosis gingival (2)

ensp00000345039	d.51	Fragile X syndrome (3)

ensp00000262017	d.200	Glanzmann thrombasthenia type B (3)

ensp00000260630	a.104*	Glaucoma 3A primary infantile 231300 (3)

ensp00000282439	c.89	Glycogen storage disease VII (3)

ensp00000297137	b.95*	GM2-gangliosidosis AB variant (3)

ensp00000220616	g.28,	Goiter adolescent multinodular (1),Goiter nonendemic simple (3),

Hypothyroidism hereditary congenital (3)

ensp00000329869	g.18	Goiter congenital (3),Hypothyroidism congenital (3),

Thrombocythemia essential 187950 (3),Thyroid iodine peroxidase deficiency (1)

ensp00000329362	a.21*	Gonadal dysgenesis XY type (3)

ensp00000302665	g.1	Growth retardation with deafness and mental retardation (3)

ensp00000260433	a.104	Gynecomastia familial due to increased aromatase activity (1),

Virilization maternal and fetal from placental aromatase deficiency (3)

ensp00000311698	d.19*	Hemochromatosis (3)

ensp00000328511	f.14	Hemolytic anemia due to hexokinase deficiency (3)

ensp00000218265	c.86*	Hemolytic anemia due to PGK deficiency (3),

Myoglobinuria/hemolysis due to PGK deficiency (3)

ensp00000309438	c.89,c.89	Hemolytic anemia due to phosphofructokinase deficiency (1)

ensp00000218099	g.32	Hemophilia B (3)

ensp00000223095	e.1*	Hemorrhagic diathesis due to PAI1 deficiency (1),

Thrombophilia due to excessive plasminogen activator inhibitor (1)

ensp00000343606	b.64	Hepatocellular carcinoma (3)

ensp00000344460	c.79	Homocystinuria B6-responsive and nonresponsive types (3)

ensp00000252444	g.12,	Hypercholesterolemia familial (3)

ensp00000274576	b.96	Hyperekplexia and spastic paraparesis (3),Startle disease autosomal recessive (3),

Startle disease/hyperekplexia autosomal dominant 149400 (3)

ensp00000261192	e.17	Hyperleucinemia-isoleucinemia or hypervalinemia (1) (?)

ensp00000280867	d.130	Hypermethioninemia persistent autosomal dominant due to

methionine adenosyltransferase I/III deficiency (3)

ensp00000297494	d.174	Hypertension pregnancy-induced (2)

ensp00000265074	c.93	Hypertension salt-resistant (1) (?)

ensp00000322991	e.17*	Hypervalinemia or hyperleucine-isoleucinemia (1) (?)

ensp00000340482	c.93	Hypocalcemia autosomal dominant 601198 (3),

Hypocalciuric hypercalcemia type I 145980 (3),Neonatal hyperparathyroidism 239200 (3)

ensp00000319814	c.109	Hypoglycemia due to PCK1 deficiency (1) (?)

ensp00000218368	b.22	Immunodeficiency X-linked with hyper-IgM (3)

ensp00000257555	a.34	Insulin-dependent diabetes mellitus (3),

MODY type 3 600496 (3),Non-insulin-dependent diabetes mellitus-2 601407 (2)

ensp00000251819	b.66,g.64	Jacobs syndrome (2)

ensp00000155840	f.14	Jervell and Lange-Nielsen syndrome 220400 (3),Long QT syndrome-1 (3)

ensp00000227868	a.9	Lacticacidemia due to PDX1 deficiency (3)

ensp00000264932	d.168	Leigh syndrome (3)

ensp00000262965	a.38	Leukemia acute lymphoblastic (1)

ensp00000282111	a.21	Leukemia acute lymphoblastic (1)

ensp00000254066	a.123	Leukemia acute promyelocytic (1)

ensp00000303507	a.87,a.147	Leukemia chronic myeloid (3)

ensp00000298467	d.93	Leukemia chronic myeloid (3)

ensp00000306443	a.154	Leukemia myeloid/lymphoid or mixed-lineage (2)

ensp00000277541	g.65	Leukemia T-cell acute lymphoblastic (2)

ensp00000264824	a.38	Leukemia T-cell acute lymphoblastoid (2)

ensp00000294339	a.38	Leukemia-1 T-cell acute lymphoblastic (3)

ensp00000216612	b.63*	Leukemia/lymphoma T-cell (2)

ensp00000331181	d.93*	Lymphoproliferative syndrome X-linked (2),{?Amyloidosis secondary susceptibility to} (1)

ensp00000343136	a.9	Maple syrup urine disease type II (3)

ensp00000261978	g.23	Marfan syndrome atypical (3)

ensp00000301873	g.23	Marfan syndrome atypical (3)

ensp00000302237	a.66	McCune-Albright polyostotic fibrous dysplasia 174800 (3),

Pituitary ACTH secreting adenoma (3),

Pseudohypoparathyroidism type Ia 103580 (3),Somatotrophinoma (3)

ensp00000285981	a.104*	Mephenytoin poor metabolizer (3)

ensp00000285979	a.104*	Mephenytoin poor metabolizer (3)

ensp00000243222	b.22	Metaphyseal chondrodysplasia Schmid type (3)

ensp00000341625	d.120*	Methemoglobinemia due to cytochrome b5 deficiency (3)

ensp00000343807	a.123	MODY type 1 125850 (3),Non-insulin-dependent diabetes mellitus 125853 (3)

ensp00000225893	a.34	MODY type 5 (3)

ensp00000258391	f.36,b.96	Myasthenia gravis neonatal transient (2)

ensp00000304290	f.36,b.96	Myasthenic syndrome slow-channel congenital 601462 (3)

ensp00000261007	f.36,b.96	Myasthenic syndrome slow-channel congenital 601462 (3)

ensp00000293780	f.36	Myasthenic syndrome slow-channel congenital 601462 (3)

ensp00000290858	b.54*	Myeloid leukemia acute M4Eo subtype (2)

ensp00000303844	g.40,g.40	Myotonic dystrophy 2 (2)

ensp00000314415	g.67	N syndrome 310465 (1) (?)

ensp00000255622	a.125	Night blindness congenital stationary type 3 163500 (3),Retinitis pigmentosa autosomal recessive (3)

ensp00000232461	a.66	Night blindness congenital stationary (3)

ensp00000334876	a.91*	Oguchi disease-2 258100 (3)

ensp00000229022	a.123	Osteoporosis involutional (1) (?),Rickets vitamin D-resistant 277440 (3)

ensp00000294304	g.12	Osteoporosis-pseudoglioma syndrome (2),[High bone mass] (2)

ensp00000338276	d.111*	Pancreatic cancer (2)

ensp00000341551	d.164	Pancreatic cancer (3)

ensp00000303960	f.37	Persistent hyperinsulinemic hypoglycemia of infancy 256450 (3)

ensp00000303500	d.178	Phenylketonuria (3),[Hyperphenylalaninemia mild] (3)

ensp00000312999	a.66	Pituitary ACTH-secreting adenoma (3)

ensp00000321853	e.1	Plasmin inhibitor deficiency (3)

ensp00000220809	g.27	Plasminogen activator deficiency (1)

ensp00000263686	g.18	Platelet alpha/delta storage pool deficiency (1)

ensp00000268053	a.104	Polycystic ovary syndrome with hyperandrogenemia (2)

ensp00000339185	c.113*	Porphyria congenital erythropoietic (3)

ensp00000263817	f.37	Progressive intrahepatic cholestasis-2 (2)

ensp00000244137	d.127	Prolidase deficiency (3)

ensp00000247153	g.60	Properdin deficiency X-linked (3)

ensp00000331152	a.38	Prostate cancer 176807 (3)

ensp00000298842	e.1*	Protein C inhibitor deficiency (2)

ensp00000330021	g.32	Protein S deficiency (3)

ensp00000228606	a.104	Pseudo-vitamin D dependency rickets 1 (2)

ensp00000281330	a.123	Pseudohypoaldosteronism type I autosomal dominant 177735 (3)

ensp00000234071	g.32	Purpura fulminans neonatal (1),Thrombophilia due to protein C deficiency (3)

ensp00000285379	b.74*	Renal tubular acidosis-osteopetrosis syndrome (3)

ensp00000255266	a.125	Retinitis pigmentosa autosomal recessive (3)

ensp00000324122	a.183	Retinitis pigmentosa-11 (2)

ensp00000229771	d.23	Retinitis pigmentosa-14 600132 (3)

ensp00000235382	a.91	Rieger syndrome type 2 (2)

ensp00000262367	g.53,a.12,a.153	Rubenstein-Taybi syndrome 180849 (3),Colorblindness protan (3),

Rett syndrome (2) (?),Rothmund-Thomson syndrome (2) (?)

ensp00000320324	b.98	Salivary gland pleomorphic adenoma (2)

ensp00000303727	f.36,b.96	Schizophrenia neurophysiologic defect in (2)

ensp00000325831	d.178	Segawa syndrome recessive (3)

ensp00000264972	d.93	Selective T-cell defect (3)

ensp00000286628	f.14	Smith-Lemli-Opitz syndrome (2)

ensp00000337994	a.114	Speech-language disorder-1 (2)

ensp00000305769	d.164	Spinal muscular atrophy distal with upper limb predominance (2)

ensp00000317862	a.68	Thrombocytopenia X-linked 313900 (3),Wiskott-Aldrich syndrome (3)

ensp00000215727	e.1	Thrombophilia due to heparin cofactor II deficiency (3)

ensp00000338087	a.104*	Thromboxane synthase deficiency (2)

ensp00000280696	a.123	Thyroid hormone resistance 274300 188570 (3)

ensp00000295600	a.38	Tietz syndrome 103500 (3),Waardenburg syndrome type IIA 193510 (3),

Waardenburg syndrome/ocular albinism digenic 103470 (3)

ensp00000260682	a.104*	Tolbutamide poor metabolizer (3)

ensp00000258070	d.93	Tylosis with esophageal cancer (2)

ensp00000261755	d.177	Tyrosinemia type I (3)

ensp00000215964	a.21	Waardenburg-Shah syndrome 277580 (3)

ensp00000289041	d.133,a.56	Xanthinuria type I (3)
