# Supplementary material for: Fold Designability, Distribution, and Disease
Source: PLoS Comput Biol. 2006 May 5;2(5):e40. doi: 10.1371/journal.pcbi.0020040 (PMC1456317; doi:10.1371/journal.pcbi.0020040)
Supplement: Supplementary file 27 [file pcbi.0020040.sd003.doc]

**Protocol S3: List of Common Disease Proteins**

Common disease proteins with detectable SCOP folds are listed here. An * in the fold column indicates that the SCOP definition highly covers the protein (see Methods).

ENSEMBL Protein	Folds	Associated OMIM Disease Annotation

ensp00000217961	c.76	Ichthyosis X-linked (3),Placental steroid sulfatase deficiency (3)

ensp00000218195	f.13*	Colorblindness blue/protan (3),Rubenstein-Taybi syndrome 180849 (3)

ensp00000229264	b.69*	{Hypertension essential susceptibility to} 145500 (3)

ensp00000233242	a.118,f.7	Abetalipoproteinemia (3),Apolipoprotein B-100 ligand-defective (3),Hyperbetalipoproteinemia	(3),Hypobetalipoproteinemia (3)

ensp00000236260	e.1*	Antithrombin III deficiency (3)

ensp00000246337	c.1*	Porphyria cutanea tarda (3),Porphyria hepatoerythropoietic (3)

ensp00000249389	f.13*	Colorblindness tritan (3)

ensp00000252444	b.68,g.3,g.12	Hypercholesterolemia familial(3)

ensp00000256993	b.1	Cardiomyopathy familial hypertrophic 4 115197 (3),Hypercholesterolemia familial (3)

ensp00000260682	a.104*	Tolbutamide poor metabolizer (3)

ensp00000261733	c.82*	Alcohol intolerance acute (3),{?Fetal alcohol syndrome} (1)

ensp00000262367	g.53,a.12,a.29,a.153	Rubenstein-Taybi syndrome 180849 (3),Colorblindness protan (3),Rett syndrome	(2) (?),Rothmund-Thomson syndrome (2) (?)

ensp00000264381	c.69	Apnea postanesthetic (3)

ensp00000264691	b.47	Factor XI deficiency (3)

ensp00000267071	a.170,b.40,a.171,b.40	Breastcancer 2 early onset (3),Pancreatic cancer (3),

Fanconi anemia type D (2)

ensp00000276343	f.13*	Colorblindness blue monochromatic (3),Colorblindness deutan (3)

ensp00000276348	f.13*	Colorblindness blue monochromatic (3),Colorblindness deutan (3)

ensp00000289928	b.1	Charcot-Marie-Tooth neuropathy-1B 118200 (3),

Dejerine-Sottas disease myelin P(0)-related 145900 (3),Hypomyelination congenital (3)

ensp00000292301	f.13*	{HIV infection susceptibility/resistence to} (3)

ensp00000292303	f.13*	{HIV infection susceptibility/resistance to} (3)

ensp00000292888	b.1	Nephrosis-1 congenital Finnish type 256300 (3)

ensp00000298844	e.1*	Emphysema (3),Emphysema-cirrhosis (3),

Hemorrhagic diathesis due to `antithrombin' Pittsburgh (3)

ensp00000299367	b.47,g.18	C2 deficiency (3)

ensp00000301141	a.104*	Coumarin resistance 122700 (3),{Nicotine addiction protection from} (3)

ensp00000306780	f.13.1,c.10	Ovarian dysgenesis hypergonadotropic with normal karyotype 233300 (3)

ensp00000314508	c.1,b.71	Gaucher disease (3),Gaucher disease with cardiovascular calcification (3)

ensp00000331327	g.37	Denys-Drash syndrome (3),Frasier syndrome 136680 (3),Wilms tumor type 1 (3)

ensp00000334146	g.37	Factor XI deficiency (3)

ensp00000340258	b.1	{Atopy susceptibility to} (3)

ensp00000342362	d.81,c.2	Favism (3),G6PD deficiency (3),Hemolytic anemia due to G6PD deficiency (3)

ensp00000343985	f.13*	{HIV infection susceptibility/resistance to} (3)

ensp00000344419	a.93	Myeloperoxidase deficiency (3)

ensp00000344867	f.13	{HIV infection susceptibility/resistence to} (3)
