# Supplementary material for: Fold Designability, Distribution, and Disease
Source: PLoS Comput Biol. 2006 May 5;2(5):e40. doi: 10.1371/journal.pcbi.0020040 (PMC1456317; doi:10.1371/journal.pcbi.0020040)
Supplement: Supplementary file 28 [file pcbi.0020040.sd004.doc]

**Protocol S4: List of Rare Disease Proteins**

Rare disease proteins with detectable SCOP folds are listed here. An * in the fold column indicates that the SCOP definition highly covers the protein (see Methods).

ENSEMBL Protein	Folds	Associated OMIM Disease Annotation

ensp00000006951	b.69*	Lissencephaly-1 (3),Miller-Dieker lissencephaly syndrome (2)

ensp00000008553	c.3	[Fish-odor syndrome] 602079 (3)

ensp00000008588	c.76	Mucopolysaccharidosis II (3)

ensp00000039007	c.78*	Ornithine transcarbamylase deficiency (3)

ensp00000053469	a.39*	Cone dystrophy-3 602093 (3)

ensp00000064324	a.4	Iridogoniodysgenesis 601631 (3)

ensp00000164139	c.87*	McArdle disease (3)

ensp00000172196	a.4	Trichodontoosseous syndrome 190320 (3)

ensp00000176643	c.82*	Sjogren-Larsson syndrome (3)

ensp00000196371	c.63	Ketoacidosis due to SCOT deficiency (3)

ensp00000205832	a.127*	Fumarase deficiency (3)

ensp00000206249	a.123,g.39 Breast cancer (1),Estrogen resistance (3)

ensp00000206765	d.3,b.1	Ichthyosiform erythroderma congenital-242100 (3),Ichthyosis lamellar AR 242300 (3)

ensp00000215754	d.80*	Persistent Mullerian duct syndrome type I 261550 (3),

Anemia pernicious congenital due to deficiency of intrinsic factor (1)

ensp00000215964	a.21	Waardenburg-Shah syndrome 277580 (3)

ensp00000215995	c.1,b.71*	Kanzaki disease (3),NAGA deficiency mild (3),Schindler disease (3)

ensp00000216124	c.76 *	Metachromatic leukodystrophy (3)

ensp00000216392	c.87*	Glycogen storage disease VI (3)

ensp00000216727	d.58	Oculopharyngeal muscular dystorphy 164300 (3),

Oculopharyngeal muscular dystrophy autosomal recessive 257950 (3)

ensp00000216951	d.142,c.30 5-oxoprolinuria 266130 (3),

Hemolytic anemia due to glutathione synthetase deficiency 231900 (3)

ensp00000217172	b.9*	Diabetes insipidus neurohypophyseal 125700 (3)

ensp00000218099	b.47,g.3,g.32;g.3	Hemophilia B (3)

ensp00000218265	c.86*	Hemolytic anemia due to PGK deficiency (3),

Myoglobinuria/hemolysis due to PGK deficiency (3)

ensp00000218368	b.22	Immunodeficiency X-linked with hyper-IgM (3)

ensp00000218425	a.87,b.55,g.50	Aarskog-Scott syndrome (3)

ensp00000218516	c.1	Fabry disease (3)

ensp00000220616	c.69,g.28	Goiter adolescent multinodular (1),Goiter nonendemic simple (3),

Hypothyroidism hereditary congenital (3)

ensp00000221496	g.17	Persistent Mullerian duct syndrome type I 261550 (3)

ensp00000221996	a.4	Cone-rod retinal dystrophy-2 120970 (3),Leber congenital amaurosis type III (3)

ensp00000222139	b.1	[Erythrocytosis familial] 133100 (3)

ensp00000222214	e.6,a.29*	Glutaricaciduria type I (3)

ensp00000222246	d.144	SCID autosomal recessive T-negative/B-positive type (3)

ensp00000224242	c.67*	Gyrate atrophy of choroid and retina with ornithinemia B6 responsive or unresponsive (3)

ensp00000225893	a.35,a.4,a.34	MODY type 5 (3)

ensp00000226413	f.13	Hypogonadotropic hypogonadism (3)

ensp00000227157	d.162,c.2	Exertional myoglobinuria due to deficiency of LDH-A (3)

ensp00000227868	c.43,b.84,a.9	Lacticacidemia due to PDX1 deficiency (3)

ensp00000228606	a.104	Pseudo-vitamin D dependency rickets 1 (2)

ensp00000228841	a.39*	Cardiomyopathy hypertrophic mid-left ventricular chamber type (3)

ensp00000229022	a.123,g.39	Osteoporosis involutional (1) (?),Rickets vitamin D-resistant 277440 (3)

ensp00000229270	c.1*	Hemolytic anemia due to triosephosphate isomerase deficiency (3)

ensp00000229725	b.68*	Sialidosis type I (3),Sialidosis type II (3)

ensp00000230732	a.35,a.4	Deafness autosomal dominant 15 602459 (3)

ensp00000230882	b.1	Laron dwarfism 262500 (3),

Short stature autosomal dominant with normal serum growth hormone binding protein (3),

Short stature idiopathic (3)

ensp00000231509	a.123,g.39	Cortisol resistance (3)

ensp00000232461	c.37,a.66	Night blindness congenital stationary (3)

ensp00000233072	d.142,c.23,a.92,c.8,c.30	Carbamoylphosphate synthetase I deficiency (3)

ensp00000233710	e.6,a.29*	Acyl-CoA dehydrogenase long chain deficiency of (3)

ensp00000234071	b.47,g.3,g.32	Purpura fulminans neonatal (1),Thrombophilia due to protein C deficiency (3)

ensp00000234736	b.6.1,b.18	Hemorrhagic diathesis due to factor V deficiency (1),

{Thromboembolism susceptibility due to factor V Leiden} (3)

ensp00000235547	c.2	3-beta-hydroxysteroid dehydrogenase type II deficiency (3)

ensp00000235701	e.6,a.29*	Acyl-CoA dehydrogenase medium chain deficiency of (3)

ensp00000235958	c.1	HMG-CoA lyase deficiency (3)

ensp00000237310	c.42*	Argininemia (3)

ensp00000239243	a.4	Craniosynostosis type 2 (3),Parietal foramina (2)

ensp00000239259	d.144,b.1	Venous malformations multiple cutaneous and mucosal 600195 (3)

ensp00000241052	e.5*	Acatalasemia (3)

ensp00000242261	a.38	Blepharophimosis epicanthus inversus and ptosis type 2 (2),

Saethre-Chotzen syndrome 101400 (3)

ensp00000242480	g.37	Neuropathy congenital hypomyelinating 1 (3)

ensp00000243929	g.3	Hereditary hemorrhagic telangiectasia-2 600376 (3),{Atherosclerosis susceptibility to} (2)

ensp00000244137	d.127	Prolidase deficiency (3)

ensp00000248419	f.13	Bleeding disorder due to defective thromboxane A2 receptor (3)

ensp00000248594	c.45	Colon cancer (3)

ensp00000248633	c.37	Adrenoleukodystrophy neonatal 202370 (3),

Refsum disease infantile 266510 (3),Zellweger syndrome-1 214100 (3)

ensp00000249505	a.4	Synpolydactyly type II 186000 (3)

ensp00000249760	e.6,a.29*	Isovalericacidemia (3)

ensp00000251020	b.121,g.37	Townes-Brocks syndrome 107480 (3)

ensp00000251654	c.14*	Propionicacidemia type II or pccB type (3)

ensp00000252857	b.1	Oguchi disease-1 258100 (3)

ensp00000253127	d.58	Dyskeratosis congenita-1 305000 (3)

ensp00000253260	c.2*	Pseudohermaphroditism male with gynecomastia (3)

ensp00000253496	b.47,g.14	Factor XII deficiency (3)

ensp00000253546	c.69*	Ceroid lipofuscinosis neuronal variant juvenile type with granular osmiophilic deposits (3),	Ceroid lipofuscinosis neuronal-1 infantile 256730 (3)

ensp00000254142	c.36*	Pyruvate dehydrogenase deficiency (3)

ensp00000254299	d.96*	Dystonia DOPA-responsive 128230 (3),

Phenylketonuria atypical due to GCH1 deficiency 233910 (1)

ensp00000254663	c.67*	Leukemia-1 T-cell acute lymphoblastic (3)

ensp00000254854	d.144,d.58 Cone dystrophy progressive (2),Leber congenital amaurosis type I 204000 (3)

ensp00000255266	a.125,d.110	Retinitis pigmentosa autosomal recessive (3)

ensp00000256216	c.2,d.106,d.38	D-bifunctional protein deficiency (3)

ensp00000256592	g.17*	Hypothyroidism nongoitrous (3)

ensp00000256635	c.45	Lymphoproliferative syndrome X-linked (2)

ensp00000256996	b.69	Xeroderma pigmentosum group E DDB-negative subtype 278740 (3)

ensp00000257340	c.120	Xeroderma pigmentosum group G 278780 (3)

ensp00000257555	a.35,a.4,a.34	Insulin-dependent diabetes mellitus (3),

MODY type 3 600496 (3),Non-insulin-dependent diabetes mellitus-2 601407 (2)

ensp00000257566	b.2	Ulnar-mammary syndrome 181450 (3)

ensp00000257904	d.144*	Melanoma (3)

ensp00000258224	e.1	{Hypertension essential susceptibility to} (3),{Preeclampsia susceptibility to} (3)

ensp00000259398	c.1*	Fructose intolerance (3)

ensp00000259463	a.6,g.39	Xeroderma pigmentosum group A (3)

ensp00000260630	a.104*	Glaucoma 3A primary infantile 231300 (3)

ensp00000261007	f.36,b.96	Myasthenic syndrome slow-channel congenital 601462 (3)

ensp00000261170	d.58,d.144	Lipoid adrenal hyperplasia 201710 (3)

ensp00000261267	d.2*	Amyloidosis renal 105200 (3)

ensp00000261416	c.1,d.92	Sandhoff disease infantile juvenile and adult forms (3),

Spinal muscular atrophy HEXB-related (3)

ensp00000261967	b.47,g.3,g.32,g.3.1	Factor VII deficiency (3)

ensp00000261978	g.23,g.3;g.3.1	Marfan syndrome atypical (3)

ensp00000261981	e.1*	Alpha-1-antichymotrypsin deficiency (3),Cerebrovascular disease occlusive (3)

ensp00000262602	c.1*	Hemolytic anemia due to ADA excess (1),

Severe combined immunodeficiency due to ADA deficiency (3)

ensp00000263374	b.42	Central core disease 117000 (3),{Malignant hyperthermia susceptibility 1} 145600 (3)

ensp00000264870	d.3,b.1	Factor XIIIA deficiency (3)

ensp00000264914	c.76*	Maroteaux-Lamy syndrome several forms (3)

ensp00000264932	c.3,d.168,a.7,c.3	Leigh syndrome (3)

ensp00000265023	d.17	[Kininogen deficiency] (3)

ensp00000265172	b.47,g.12	C3b inactivator deficiency (3)

ensp00000265573	a.118	Sorsby fundus dystrophy 136900 (3)

ensp00000265833	d.16,c.3	Brunner syndrome (3)

ensp00000265838	c.95*	3-ketothiolase deficiency (3)

ensp00000265849	d.122,d.14	Colorectal cancer hereditary nonpolyposis type 4 (3),

Turcot syndrome with glioblastoma 276300 (3)

ensp00000266085	b.40*	Sorsby fundus dystrophy 136900 (3)

ensp00000266950	a.4	Pancreatic agenesis 260370 (3),Lacticacidemia due to PDX1 deficiency (3)

ensp00000267008	d.144	Hereditary hemorrhagic telangiectasia-2 600376 (3)

ensp00000267163	a.74	Bladder cancer 109800 (3),Osteosarcoma 259500 (2),

Pinealoma with bilateral retinoblastoma(2),Retinoblastoma (3)

ensp00000268058	g.44	Leukemia acute promyelocytic PML/RARA type (3)

ensp00000268097	c.1,d.92	GM2-gangliosidosis juvenile adult (3),Tay-Sachs disease (3),[Hex A pseudodeficiency] (1)

ensp00000268476	c.67	Tyrosinemia type II (3)

ensp00000268695	c.76	Mucopolysaccharidosis IVA (3)

ensp00000268704	c.37	Spastic paraplegia-7 (3)

ensp00000269305	b.2,a.53	Colorectal cancer 114500 (3),Li-Fraumeni syndrome (3)

ensp00000269385	b.34	Obestiy with impaired prohormone processing 600955 (3)

ensp00000269571	d.144,c.10,g.3	Sialidosis type I (3),Sialidosis type II (3)

ensp00000269980	c.36	Maple syrup urine disease type Ia (3)

ensp00000270142	b.1*	Amytrophic lateral sclerosis due to SOD1 deficiency 105400 (3)

ensp00000271064	d.3	Argininemia (3)

ensp00000271448	c.3	Porphyria variegata 176200 (3)

ensp00000271509	g.18	Factor XIIIB deficiency (3)

ensp00000271651	d.3	Pycnodysostosis 265800 (3)

ensp00000272425	d.108	Fabry disease (3)

ensp00000272852	c.56*	Coproporphyria (3),Harderoporphyrinuria (3)

ensp00000273480	g.44*	Oguchi disease-1 258100 (3)

ensp00000274376	a.116,d.93,b.55	Basal cell carcinoma (3)

ensp00000274576	b.96	Hyperekplexia and spastic paraparesis (3),Startle disease autosomal recessive (3),

Startle disease/hyperekplexia autosomal dominant 149400 (3)

ensp00000274813	c.1,c.23*	Methylmalonicaciduria mutase deficiency type (3)

ensp00000276449	d.129*	Lipoid adrenal hyperplasia 201710 (3)

ensp00000277268	d.109	Amyloidosis Finnish type 105120 (3)

ensp00000277315	c.1*	Porphyria acute hepatic (3),{Lead poisoning susceptibility to} (3)

ensp00000277865	c.58,c.2	Hyperinsulinism-hyperammonemia syndrome (3)

ensp00000277940	a.64	Gaucher disease variant form (3),

Metachromatic leukodystrophy due to deficiency of SAP-1 (3)

ensp00000278017	a.104*	Adrenal hyperplasia congenital due to 17-alpha-hydroxylase deficiency (3)

ensp00000278715	c.94,d.50*	Porphyria acute intermittent (3)

ensp00000280362	d.96*	Phenylketonuria due to PTS deficiency (3)

ensp00000280496	a.4	Rhabdomyosarcoma alveolar 268220 (3)

ensp00000280696	a.123,g.39	Thyroid hormone resistance 274300 188570 (3)

ensp00000280867	d.130	Hypermethioninemia persistent autosomal dominant due to methionine

adenosyltransferase I/III deficiency (3)

ensp00000281243	c.2*	Phenylketonuria due to dihydropteridine reductase deficiency (3)

ensp00000281330	a.123,g.39 Pseudohypoaldosteronism type I autosomal dominant 177735 (3)

ensp00000283871	b.82*	Alkaptonuria (3)

ensp00000285379	b.74*	Renal tubular acidosis-osteopetrosis syndrome (3)

ensp00000289041	d.133,d.145,d.41,d.87,a.56,d.15	Xanthinuria type I (3)

ensp00000289473	d.189,b.34	Chronic granulomatous disease due to deficiency of NCF-1 (3)

ensp00000290524	a.4	Bare lymphocyte syndrome complementation group C (1)

ensp00000292144	b.1	Immunodeficiency due to defect in CD3-gamma (3)

ensp00000292327	a.39*	Cardiomopathy hypertrophic mid-ventricular chamber type (3)

ensp00000292427	a.104	Adrenal hyperplasia congenital due to 11-beta-hydroxylase deficiency (3),

Aldosteronism glucocorticoid-remediable(3)

ensp00000293288	f.1*	Colorectal cancer (3)

ensp00000293780	f.36	Myasthenic syndrome slow-channel congenital 601462 (3)

ensp00000294066	d.144	Hyperinsulinism familial 602485 (3),MODY type 2 125851 (3)

ensp00000294339	a.38	Leukemia-1 T-cell acute lymphoblastic (3)

ensp00000294782	a.77*	Albinism ocular autosomal recessive (3),Albinism oculocutaneous type II (3)

ensp00000294954	f.13,c.10	Leydig cell hypoplasia (3),Precocious puberty male 176410 (3)

ensp00000295382	a.39*	Persistent Mullerian duct syndrome type I 261550 (3)

ensp00000295600	a.38	Tietz syndrome 103500 (3),

Waardenburg syndrome type IIA 193510 (3),

Waardenburg syndrome/ocular albinism digenic 103470 (3)

ensp00000295754	d.144,g.7	Colon cancer (3),Colorectal cancer familial nonpolyposis type 6 (3)

ensp00000296271	f.13*	Night blindness congenital stationery rhodopsin-related (3),

Retinitis pigmentosa autosomal recessive (3),Retinitis pigmentosa-4 autosomal dominant (3)

ensp00000297137	b.95*	GM2-gangliosidosis AB variant (3)

ensp00000297283	c.60*	Myopathy due to phosphoglycerate mutase deficiency (3)

ensp00000298467	d.144,d.93,b.34	Leukemia chronic myeloid (3)

ensp00000298556	c.61*	HPRT-related gout (3),Lesch-Nyhan syndrome (3)

ensp00000299299	d.74*	Hyperphenylalaninemia due to pterin-4a-carbinolamine dehydratase deficiency 264070 (3)

ensp00000301873	g.23,g.3	Marfan syndrome atypical (3)

ensp00000302036	c.55*	Glycerol kinase deficiency (3)

ensp00000302237	c.37,a.66	McCune-Albright polyostotic fibrous dysplasia 174800 (3),

PituitaryACTH secreting adenoma (3),

Pseudohypoparathyroidism type Ia 103580 (3),Somatotrophinoma (3)

ensp00000302257	c.55	Hemolytic anemia due to hexokinase deficiency (3)

ensp00000302390	a.123	Adrenal hypoplasia congenital with hypogonadotropic hypogonadism (3)

ensp00000302728	b.18,c.1,b.1	Mucopolysaccharidosis VII (3)

ensp00000302777	c.32,d.79	[Red hair/fair skin] (3),{UV-induced skin damage vulnerability to} (3)

ensp00000302942	a.4	Schizencephaly (3)

ensp00000303500	d.178,d.58	Phenylketonuria (3),[Hyperphenylalaninemia mild] (3)

ensp00000303532	g.9*	Gaucher disease variant form (3),

Metachromatic leukodystrophy due to deficiency of SAP-1 (3)

ensp00000303875	a.104*	Adrenal hyperplasia congenital due to 21-hydroxylase deficiency (3),

Carbamoylphosphate synthetase I deficiency (3)

ensp00000304290	f.36,b.96	Myasthenic syndrome slow-channel congenital 601462 (3)

ensp00000304973	c.45,b.7*	Bannayan-Zonana syndrome 153480 (3),Cowden disease 158350 (3),

Endometrial carcinoma (3),Lhermitte-Duclos syndrome (3)

ensp00000306099	d.171	Dysfibrinogenemia beta type (3)

ensp00000306361	d.171	Amyloidosis hereditary renal 105200 (3),

Dysfibrinogenemia alpha type causing bleeding diathesis (3),

Dysfibrinogenemia alpha type causing recurrent thrombosis (3)

ensp00000306477	d.160	Biotinidase deficiency (3)

ensp00000307786	a.3*	Multiple carboxylase deficiency biotin-responsive (3)

ensp00000308024	c.41,b.18,d.58	Obestiy with impaired prohormone processing 600955 (3)

ensp00000308554	d.142,c.30,b.84	Propionicacidemia type I or pccA type (1)

ensp00000308576	c.37*	Night blindness congenital stationery rhodopsin-related (3),

Retinitis pigmentosa autosomal recessive (3),Retinitis pigmentosa-4 autosomal dominant (3)

ensp00000308619	g.50	Alpha-thalassemia/mental retardation syndrome type 2 301040 (3),

Juberg-Marsidi syndrome 309590 (3)

ensp00000309757	c.69	Chylomicronemia syndrome familial (3),

Combined hyperlipemia familial (3),Hyperlipoproteinemia I (1),

Lipoprotein lipase deficiency (3)

ensp00000309845	c.37*	Bladder cancer 109800 (3)

ensp00000311280	a.4	Familial Mediterranean fever (3)

ensp00000311290	a.4	Pituitary hormone deficiency combined (3)

ensp00000312673	a.26*	Isolated growth hormone deficiency Illig type with absent GH

and Kowarski type with bioinactive GH (3)

ensp00000312999	c.37,a.66	Pituitary ACTH-secreting adenoma (3)

ensp00000313026	c.2*	Galactose epimerase deficiency (3)

ensp00000313547	a.118,b.34	Chronic granulomatous disease due to deficiency of NCF-2 (1)

ensp00000313925	a.123,g.39	Androgen insensitivity several forms (3),

Breast cancer male with Reifenstein syndrome (3),Perineal hypospadias (3),

Prostate cancer (3),Spinal and bulbar muscular atrophy of Kennedy 313200 (3)

ensp00000314606	c.76*	Sanfilippo syndrome type A (3)

ensp00000315029	d.169,g.3	Thrombophilia due to thrombomodulin defect (3)

ensp00000315680	b.69*	Rhizomelic chondrodysplasia punctata type 1 215100 (3)

ensp00000316786	c.2	Apparent mineralocorticoid excess hypertension due to (3)

ensp00000317862	b.55,a.68	Thrombocytopenia X-linked 313900 (3),Wiskott-Aldrich syndrome (3)

ensp00000318318	b.82*	Carbohydrate-deficient glycoprotein syndrome type Ib 602579 (3)

ensp00000318351	c.36,c.48	Maple syrup urine disease type Ib (3)

ensp00000318568	b.47*	Pancreatitis hereditary 167800 (3),Trypsinogen deficiency (1)

ensp00000319034	b.1	Kostmann neutropenia 202700 (3)

ensp00000319075	b.1	Immunodeficiency T-cell receptor/CD3 complex (3)

ensp00000319899	d.13;d.13*	Galactosemia (3)

ensp00000320187	c.1*	Aldolase A deficiency (3)

ensp00000320568	c.55	Glycerol kinase deficiency (3)

ensp00000321853	e.1	Plasmin inhibitor deficiency (3)

ensp00000321935	c.37	Griscelli disease 214450 (3)

ensp00000324856	d.144	Peutz-Jeghers syndrome 175200 (3)

ensp00000324895	g.9*	Gaucher disease variant form (3),

Metachromatic leukodystrophy due to deficiency of SAP-1 (3)

ensp00000325136	c.95*	Trifunctional protein deficiency type II (3)

ensp00000325831	d.178	Segawa syndrome recessive (3)

ensp00000327074	c.80*	Hemolytic anemia due to glucosephosphate isomerase deficiency (3),

Hydrops fetalis one form (1)

ensp00000327758	a.4	Atrial septal defect with atrioventricular conduction defects 108900 (3)

ensp00000328107	d.15	Parkinson disease juvenile type 2 600116 (3)

ensp00000328511	d.42,f.14	Hemolytic anemia due to hexokinase deficiency (3)

ensp00000328662	c.68	Chondrosarcoma 215300 (3),Exostoses multiple type 1 (3)

ensp00000329380	c.10	Bernard-Soulier syndrome (3)

ensp00000329869	a.93,g.18,g.3	Goiter congenital (3),Hypothyroidism congenital (3),

Thrombocythemia essential 187950 (3),Thyroid iodine peroxidase deficiency (1)

ensp00000329968	d.144	Glycogenosis hepatic autosomal (3)

ensp00000330382	g.17	Dermatofibrosarcoma protuberans (3),Giant-cell fibroblastoma (3),Meningioma SIS-related (3)

ensp00000331181	d.93*	Lymphoproliferative syndrome X-linked (2),{?Amyloidosis secondary susceptibility to} (1)

ensp00000332369	c.67	Anemia sideroblastic/hypochromic (3)

ensp00000334876	a.91,d.144*	Oguchi disease-2 258100 (3)

ensp00000335505	a.4	Langer mesomelic dysplasia 249700 (3),Leri-Weill dyschondrosteosis 127300 (3),

Short stature idiopathic familial (3)

ensp00000336575	c.35*	Hemolytic anemia due to glucosephosphate isomerase deficiency (3),

Hydrops fetalis one form (1)

ensp00000336709	d.142,b.84,c.30,b.84	Pyruvate carboxylase deficiency (3)

ensp00000336829	d.171	Dysfibrinogenemia gamma type (3),Hypofibrinogenemia gamma type (3)

ensp00000337247	d.144,b.1	Pfeiffer syndrome 101600 (3),Ichthyosis vulgaris 146700 (1) (?)

ensp00000338009	c.55	Hyperinsulinism familial 602485 (3),MODY type 2 125851 (3)

ensp00000338072	f.13	Diabetes insipidus nephrogenic (3)

ensp00000338387	d.104	Multiple carboxylase deficiency biotin-responsive (3)

ensp00000339824	d.144,b.1	Achondroplasia 100800 (3),Craniosynostosis nonsyndromic (3),

Crouzon syndrome with acanthosis nigricans (3),Hypochondroplasia 146000 (3),

Thanatophoric dysplasia types I and II 187600 (3)

ensp00000339996	d.6	Creutzfeldt-Jakob disease 123400 (3),Gerstmann-Straussler disease 137440 (3),

Insomnia fatal familial (3),5-oxoprolinuria 266130 (3),

Hemolytic anemia due to glutathione synthetase deficiency 231900 (3)

ensp00000340482	c.93	Hypocalcemia autosomal dominant 601198 (3),

Hypocalciuric hypercalcemia type I 145980 (3),Neonatal hyperparathyroidism 239200 (3)

ensp00000340690	b.2	Leukemia acute myeloid (3)

ensp00000341625	d.120*	Methemoglobinemia due to cytochrome b5 deficiency (3)

ensp00000342092	a.4	Craniofacial-deafness-hand syndrome 122880 (3),Rhabdomyosarcoma alveolar268220 (3),	Waardenburg syndrome type I (3),Waardenburg syndrome type III 148820 (3)

ensp00000342172	b.2.5	Cleidocranial dysplasia 119600 (3)

ensp00000342269	c.1*	Androgen insensitivity several forms (3),Breast cancer male with Reifenstein syndrome (3),	Perineal hypospadias (3),Prostate cancer (3),

Spinal and bulbar muscular atrophy of Kennedy 313200 (3)

ensp00000342297	a.4	Gaucher disease variant form (3),

Metachromatic leukodystrophy due to deficiency of SAP-1 (3)

ensp00000342568	g.17*	Hypogonadism hypergonadotropic (3),

Male pseudohermaphroditism due to defective LH (1) (?)

ensp00000343136	c.43,b.84,a.9	Maple syrup urine disease type II (3)

ensp00000343420	c.3.1,d.16	Choroideremia (3)

ensp00000343671	b.69	Cone dystrophy progressive X-linked 1 (2),Cone-rod retinal dystrophy-2 120970 (3),

Leber congenital amaurosis type III (3),Retinitis pigmentosa-15 (2),Retinitis pigmentosa-3 (3)

ensp00000343711	a.4.1,g.39	Nail-patella syndrome with open-angle glaucoma 137750 (3),

Nail-patella syndrome 161200 (3)

ensp00000343807	a.123,g.39	MODY type 1 125850 (3),Non-insulin-dependent diabetes mellitus 125853 (3)

ensp00000343888	d.122,d.14,a.21	Colorectal cancer hereditary nonpolyposis type 3 (3)

ensp00000343922	f.13	Hirschsprung disease-2 600155 (3)

ensp00000343937	c.76	Hypophosphatasia adult 146300 (1) (?),Hypophosphatasia infantile 241500 (3)

ensp00000344281	a.39*	Melanoma 155601 (3)

ensp00000344313	d.14,d.58*	Mevalonicaciduria (3)

ensp00000344379	b.82*	Alkaptonuria (3)

ensp00000344460	c.79	Homocystinuria B6-responsive and nonresponsive types (3)

ensp00000344708	c.68	Exostoses multiple type 2 (3)

ensp00000344751	b.69,b.1	Myopathy congenital (3)

ensp00000345166	a.39*	Melanoma 155601 (3)

ensp00000345169	a.77,g.24	{Autoimmune lymphoproliferative syndrome} (3)

ensp00000345489	a.127*	Argininosuccinicaciduria (3)

ensp00000345997	d.144	Myotonic dystrophy (3)
