# Supplementary material for: Fold Designability, Distribution, and Disease
Source: PLoS Comput Biol. 2006 May 5;2(5):e40. doi: 10.1371/journal.pcbi.0020040 (PMC1456317; doi:10.1371/journal.pcbi.0020040)
Supplement: Supplementary file 29 [file pcbi.0020040.sd005.doc]

**Protocol S5: Trend Exceptions**

Although we've shown that folds of greater designability tend to be more widespread within proteomes certain exceptions are known. These exceptions can be more readily spotted if one subdivides the set of folds into classes or if one examines individual folds or families (Supplementary Figure S23). For example, even with our strict method of SCOP fold detection (see Methods), the C2H2 zinc finger fold was found in over 500 human proteins, a much higher than expected quantity given that less than 5 families belonging to the fold are currently known (Supplementary Figure S12). Although it seems that the zinc finger fold is somewhat constrained in its ability to sequence divergence (probably due to its functional involvement in molecular interactions), the need for functions carried out by such fold in eukaryotic genomes has maintained its high abundance.

We also profiled SCOP folds across genomes. SCOP folds with greater numbers of families appeared more often in a set of arbitrarily selected proteomes (Supplementary Figure S24), consistent with the idea that more designable folds should be more widespread. In contrast, we found that families belonging to folds with greater numbers of families were not present more often across the same proteomes. These results suggest that individual selective pressures for functions carried out by certain SCOP families also influence the abundance and appearance of these families in organisms.
