# Supplementary material for: Fold Designability, Distribution, and Disease
Source: PLoS Comput Biol. 2006 May 5;2(5):e40. doi: 10.1371/journal.pcbi.0020040 (PMC1456317; doi:10.1371/journal.pcbi.0020040)
Supplement: Supplementary file 30 [file pcbi.0020040.sd006.doc]

**Supporting Information**

**Supplementary Figures S1-S12**

Number of Families

| Fold Group | Number of Families |
| --- | --- |
| All | 13.825 |
| Ancient | 17.998 |
| Human,Mouse,Yeast | 2.453 |

**Figure S1. Number of Families and Fold Age**

The mean number of families found in all, ancient (see Methods) and human/mouse/yeast only folds are shown. The mean number of families in all three groups are significantly different from each other (MW-test, KS-test: *p* < 0.01) with ancient folds having the most families. Standard deviations of family counts are 19, 21, and 3 amongst all, ancient and human/mouse/yeast folds, respectively.

Mean Sequence Identity (%)

| Number of Families | Human-Mouse Folds | Human-Mouse Families |
| --- | --- | --- |
| 1 | 81 | 78.1 |
| >1 | 75.7 | 75 |

**Figure S2. Divergence of SCOP Folds and Families within a Time Interval**

SCOP domains found in Ensembl human proteins likely to be mammalian in origin were compared to orthologous domains in mouse and the average divergence was recorded (see Methods). Only domains found in human and mouse and not in yeast or a number prokaryotes (see Methods) were considered likely to be mammalian in origin. The SCOP folds were divided into a number of bins according to the number of families that they contain (x-axis). The mean of the sequence identities associated with domains in each bin is shown (y-axis). At the fold level (black bars), folds with more than one family were more divergent than folds containing only one family. This trend is considered marginally significant (MW-test: *p* < 0.05, KS-test: *p* < 0.15). At the family level, families belonging to folds more than one family were more divergent than those belonging to folds containing only one family (white bar). This trend was found to be significant (MW-test, KS-test: *p* < 0.01). Similar trends were observed when we considered only domains found in human, mouse and yeast. However significance could not be established for unknown reasons.

Mean Sequence Identity (%)

| Number of Families | Yeast |
| --- | --- |
| 1 | 0.429 |
| >1 | 0.4303 |
| >5 | 0.4391 |
| >10 | 0.4115 |

Mean Sequence Identity (%)

| Number of Families | Mouse |
| --- | --- |
| 1 | 0.8502 |
| >1 | 0.835 |
| >5 | 0.833 |
| >10 | 0.828 |

**Figure S3. Mean Sequence Divergence and Family Counts of all folds**

SCOP folds found in Ensembl human proteins were compared to those in mouse and yeast orthologs and the average divergence was recorded for each fold (see Methods). The SCOP folds were divided into a number of bins according to the number of families that they contain (x-axis). The mean of the sequence identities in each bin is shown (y-axis) for mouse and yeast. For mouse, as the number of families in a SCOP fold increases, the sequences that encode the fold become more divergent. For yeast, a relatively sharp drop in sequence identity scores occurs beyond a family count of 10. Statistical significance of the trends could not be established.

Mean Fold Occurrence

| Number of Families | Human | Mouse | Yeast |
| --- | --- | --- | --- |
| 1 | 10.3 | 8.8 | 3.5 |
| >1 | 76.2 | 60.7 | 14.3 |
| >5 | 144.5 | 107 | 23.6 |
| >10 | 228.1 | 159.3 | 32.9 |

**Figure S4. Fold occurrence and Family counts of Ancient Folds**

Ancient SCOP folds were divided into a number of bins according to the number of families that they contain (x-axis). For each bin, the mean fold occurrence (the number of proteins the SCOP folds appear in) for human, mouse and yeast proteins is shown. As the number of families in a SCOP fold increases, its occurrence in the proteome tends to increase. The differences in fold occurrence between human, mouse and yeast are larger for folds with larger numbers of families. The differences between folds with one family and folds with more than one family within and between the three eukaryotes are significant (MW-test, KS-test: *p* < 0.001).

Mean Family Duplication

| Number of Families | Human | Mouse | Yeast |
| --- | --- | --- | --- |
| 1 | 1.302 | 1.282 | 1.189 |
| >1 | 1.922 | 1.698 | 1.193 |
| >5 | 2.142 | 1.781 | 1.217 |
| >10 | 2.372 | 1.878 | 1.271 |

**Figure S5. Family Duplication and Family Counts on Ancient Folds**

Ancient SCOP folds were divided into a number of bins according to the number of families that they contain (x-axis). The maximum number of times SCOP families in each fold bin were reused in the same protein was counted. For each bin, the mean count for human, mouse and yeast proteins is shown. As the number of families in a SCOP fold increases, the maximum number of times families belonging to that fold is duplicated in proteins also tends to increase. However, the significance of this trend could not be established. The differences in SCOP family duplication between human, mouse and yeast are larger for folds with larger numbers of families but significance of this trend could not be established.

Mean Family Occurrence

| Number of Families | Human | Mouse | Yeast |
| --- | --- | --- | --- |
| 1 | 18.12 | 13.45 | 2.85 |
| >1 | 25.98 | 21.09 | 4.91 |
| >5 | 26.71 | 22.25 | 5.82 |
| >10 | 28.39 | 24.03 | 6.33 |

**Figure S6. Family occurrence and Family Counts of All Folds**

SCOP folds were divided into a number of bins according to the number of families that they contain (x-axis). For each bin, the mean family occurrence (the mean number of proteins, the SCOP families in these folds appear in) for human, mouse and yeast proteins is shown. As the number of families in a SCOP fold increases, the occurrence of families belonging to these folds in the proteome tends to increase although statistical the significance of this trend could not be established. The differences in mean family occurrence between mammals and yeast tend to be larger for folds with larger numbers of families. These differences between folds of one family and those of more than one family were significant (MW-test: *p* < 0.1; KS-test: *p* < 0.001).

Mean Fold Promiscuity

| Number of Families | Human | Mouse | Yeast |
| --- | --- | --- | --- |
| 1 | 2.7 | 2.8 | 1.6 |
| >1 | 7.2 | 6.7 | 3.1 |
| >5 | 11.7 | 10.4 | 4.6 |
| >10 | 16.8 | 14.9 | 5.5 |

**Figure S7. Fold Promiscuity and Family Counts of ALL Folds**

SCOP folds were divided into a number of bins according to the number of families that they contain (x-axis). SCOP folds are connected to other “partner” folds in the same protein. The mean promiscuities (the number of unique partner folds a SCOP fold has) of folds in human, mouse and yeast are plotted. As the number of families in a SCOP fold increases, its promiscuity tends to increase. The differences in fold promiscuity between human, mouse and yeast are larger for folds with larger numbers of families. All promiscuity differences shown here between folds with one family and folds with more than one family are significant (MW-test, KS-test: *p* < 0.05).

Mean Family Promiscuity

| Number of Families | Human | Mouse | Yeast |
| --- | --- | --- | --- |
| 1 | 1.977 | 2.025 | 1.757 |
| >1 | 3.067 | 3.003 | 2.015 |
| >5 | 3.243 | 3.106 | 2.054 |
| >10 | 3.314 | 3.169 | 1.949 |

**Figure S8. Family Promiscuity and Family Counts of Ancient Folds**

SCOP folds were divided into a number of bins according to the number of families that they contain (x-axis). SCOP families are connected to other “partner” families in the same protein. The mean promiscuities (the number of unique partner families a SCOP family has) of families in human, mouse and yeast are plotted. As the number of families in a SCOP fold increases, the promiscuity of its families tends to increase. However, of the three species, only human family promiscuity differences between folds with one family and folds with more than one family were found to be significant (MW-test, KS-test: *p* < 0.07). The differences in family promiscuity between human, mouse and yeast are larger for folds with larger numbers of families. The family promiscuity differences between folds of one and more than one family between mammals and yeast were found to be significant (MW-test, KS-test: *p* < 0.05).

Mean Family Promiscuity

| Number of Families | Human | Mouse | Yeast |
| --- | --- | --- | --- |
| 1 | 2.852 | 2.888 | 1.612 |
| >1 | 3.527 | 3.473 | 2.007 |
| >5 | 3.541 | 3.395 | 2.042 |
| >10 | 3.594 | 3.451 | 1.943 |

**Figure S9. Family Promiscuity and Family Counts of All Folds**

SCOP folds were divided into a number of bins according to the number of families that they contain (x-axis). SCOP families are connected to other “partner” families in the same protein. The mean promiscuities (the number of unique partner families a SCOP family has) of families in human, mouse and yeast are plotted. As the number of families in a SCOP fold increases, the promiscuity of its families tends to increase. The differences in family promiscuity between human, mouse and yeast are larger for folds with larger numbers of families. Statistical significance of the trends could not be established.

Mean Fold Duplication

| Number of Families | Human | Mouse | Yeast |
| --- | --- | --- | --- |
| 1 | 1.9 | 1.7 | 1.1 |
| >1 | 3.2 | 2.5 | 1.5 |
| >5 | 5.2 | 3.2 | 1.6 |
| >10 | 8.1 | 3.8 | 1.9 |

**Figure S10. Fold Duplication and Family Counts of ALL Folds**

SCOP folds were divided into a number of bins according to the number of families that they contain (x-axis). The maximum number of times each fold is reused in the same protein was counted. The mean count for each bin is shown for human, mouse and yeast proteins. As the number of families in a SCOP fold increases, the maximum number of times the fold is duplicated in proteins also tends to increase. The differences in magnitude of duplication between folds with one family and folds with more than one family are significant (MW-test, KS-test: *p* < 0.05). The differences in fold duplication between human, mouse and yeast are larger for folds with larger numbers of families. The increase in fold duplication in mammals compared with yeast between folds with one family and folds with more than one family are significant (MW-test: *p* < 0.05; KS-test: *p* < 0.001). The differences between human and mouse were much less pronounced achieving significance only between folds of one family and those of more than ten families (MW-test: *p* < 0.1; KS-test: *p* < 0.001).

Mean Family Duplication

| Number of Families | Human | Mouse | Yeast |
| --- | --- | --- | --- |
| 1 | 1.8 | 1.66 | 1.13 |
| >1 | 1.98 | 1.83 | 1.24 |
| >5 | 2.16 | 1.86 | 1.25 |
| >10 | 2.39 | 1.96 | 1.29 |

**Figure S11. Family Duplication and Family Counts of ALL Folds**

SCOP folds were divided into a number of bins according to the number of families that they contain (x-axis). The maximum number of times SCOP families in each fold bin were reused in the same protein was counted. For each bin, the mean count for human, mouse and yeast proteins is shown. As the number of families in a SCOP fold increases, the maximum number of times families belonging to that fold is duplicated in proteins also tends to increase. However, the significance of this trend could not be established. The differences in SCOP family duplication between human, mouse and yeast are larger for folds with larger numbers of families. These differences were found to be significant between folds of one and more than one family (MW-test, KS-test: *p* < 0.05).

Mean Fold Occurrence

| Number of Families | Human | Mouse | Yeast |
| --- | --- | --- | --- |
| 1 | 18.1 | 20 | 3.6 |
| >1 | 77 | 58 | 13 |
| >5 | 139.8 | 105 | 22 |
| >10 | 215 | 157 | 30 |

**Figure S12. Fold occurrence and Family counts of All Folds**

SCOP folds were divided into a number of bins according to the number of families that they contain (x-axis). For each bin, the mean fold occurrence (the number of proteins the SCOP folds appear in) for human, mouse and yeast proteins is shown. As the number of families in a SCOP fold increases, its occurrence in the proteome tends to increase. The differences in fold occurrence between human, mouse and yeast are larger for folds with larger numbers of families. The differences between folds with one family and folds with more than one family within human and between the three eukaryotes are significant (MW-test, KS-test: *p* < 0.001).

**Supporting Information**

**Supplementary Figures S13-S17**

This section repeats the analysis of eukaryotes using proteomes from *E. coli*, *Y. pseudotuberculosis IP32953* and *V. vulnificus YJ016*. In these bacteria, we find hints that as families in folds increase, the folds and families within these folds tend to become more divergent, occur more often in proteomes and are more promiscuous. Similar trends were not observed for duplications within proteins perhaps because duplication is a relative rare event in bacteria compared to eukaryotes.

Mean Sequence Identity (%)

| Number of Families | Yersina |
| --- | --- |
| 1 | 81.4 |
| >1 | 74.8 |
| >5 | 74.5 |
| >10 | 74.6 |

Mean Seqence Identity (%)

| Number of Families | Vibrio |
| --- | --- |
| 1 | 67.4 |
| >1 | 61.7 |
| >5 | 61.9 |
| >10 | 62.2 |

**Figure S13. Mean Sequence Divergence and Family Counts of Ancient folds**

Ancient SCOP folds found in *E. coli* proteins were compared to those in *Vibrio vulnificus YJ016* and *Yersinia pseudotuberculosis IP32953* orthologs and the average divergence was recorded for each fold (see Methods). The SCOP folds were divided into a number of bins according to the number of families that they contain (x-axis). The mean of the sequence identities in each bin is shown (y-axis). For both *Vibrio* and *Yersinia*, as the number of families in a SCOP fold increases, the sequences that encode the fold become more divergent. The difference in conservation between folds of one family and those of more than one family are significant (MW-test: *p* < 0.02; KS-test: *p* < 0.01). With respect to *E. coli*-*Yersinia* divergence, similar significant trends (MW-test, KS-test: *p* < 0.04) were also observed when all folds were considered. With respect to divergence of all folds between *E. coli* and the more distant *Vibrio* species, similar trends were observed but significance could not be established.

Mean Fold Occurrence

| Number of Families | E. coli | Yersinia | Vibrio |
| --- | --- | --- | --- |
| 1 | 2.4 | 2.4 | 2 |
| >1 | 12.2 | 10.7 | 11.3 |
| >5 | 20.6 | 18.5 | 18.2 |
| >10 | 30.5 | 27.2 | 26.3 |

**Figure S14. Fold occurrence and Family counts of γ-Proteobacterial Folds**

Ancient SCOP folds were divided into a number of bins according to the number of families that they contain (x-axis). For each bin, the mean fold occurrence (the number of proteins the SCOP folds appear in) for *E. coli,* *Y. pseudotuberculosis IP32953* and *V. vulnificus YJ016* proteins is shown. As the number of families in a SCOP fold increases, its occurrence in the proteome tends to increase. The differences in fold occurrence between *E. coli*, *Yersinia* and *Vibrio* are larger for folds with larger numbers of families. The differences between folds with one family and folds with more than ten families within each bacteria and between the three bacteria are significant (MW-test, KS-test: *p* < 0.02).

Mean Family Occurrence

| Number of Families | E. coli | Yersinia | Vibrio |
| --- | --- | --- | --- |
| 1 | 2.4 | 2 | 2.4 |
| >1 | 3.5 | 3.7 | 3.4 |
| >5 | 3.6 | 3.9 | 3.5 |
| >10 | 3.9 | 4.6 | 3.8 |

**Figure S15. Family occurrence and Family counts of γ-Proteobacterial Folds**

SCOP folds were divided into a number of bins according to the number of families that they contain (x-axis). For each bin, the mean family occurrence (the mean number of proteins, the SCOP families in these folds appear in) for *E. coli*, *Y. pseudotuberculosis IP32953* and *V. vulnificus YJ016* proteins is shown. As the number of families in a SCOP fold increases, the occurrence of families belonging to these folds in the proteome tends to increase. Significant (MW-test: *p* < 0.1, KS-test: *p* < 0.01) occurrence differences were found between folds of one and more than one family in *E. coli* and *Yersinia*. The trend for *V. vulnificus* was found to be much weaker(MW-test: *p* < 0.19, KS-test: *p* < 0.01).

Mean Fold Promiscuity

| Number of Families | E. coli | Yersinia | Vibrio |
| --- | --- | --- | --- |
| 1 | 1.5 | 1.6 | 1.5 |
| >1 | 4.1 | 4 | 3.8 |
| >5 | 6.6 | 6.4 | 5.8 |
| >10 | 9.3 | 8.8 | 7.5 |

**Figure S16. Fold Promiscuity and Family Counts of γ-Proteobacterial Folds**

SCOP folds were divided into a number of bins according to the number of families that they contain (x-axis). SCOP folds are connected to other “partner” folds in the same protein. The mean promiscuities (the number of unique partner folds a SCOP fold has) of folds in *E. coli*, *Y. pseudotuberculosis IP32953* and *V. vulnificus YJ016* are plotted. As the number of families in a SCOP fold increases, its promiscuity tends to increase. The differences in fold promiscuity between *E. coli*, *Y. pseudotuberculosis IP32953* and *V. vulnificus YJ016* are larger for folds with larger numbers of families. The promiscuity differences between and within each bacteria between folds with one family and folds with more than ten families are significant (MW-test, KS-test: *p* < 0.02).

Mean Family Promiscuity

| Number of Families | E. coli | Yersinia | Vibrio |
| --- | --- | --- | --- |
| 1 | 1.5 | 1.5 | 1.6 |
| >1 | 2 | 2 | 2 |
| >5 | 2.1 | 2.1 | 2.1 |
| >10 | 2.1 | 2 | 2.1 |

**Figure S17. Family Promiscuity and Family Counts of γ-Proteobacterial Folds**

SCOP folds were divided into a number of bins according to the number of families that they contain (x-axis). SCOP families are connected to other “partner” families in the same protein. The mean promiscuities (the number of unique partner families a SCOP family has) of families in *E. coli*, *Y. pseudotuberculosis IP32953* and *V. vulnificus YJ016* are plotted. As the number of families in a SCOP fold increases, the promiscuity of its families tends to increase. Mean family promiscuity between folds of one family and folds of more than one family were found to be significant for *E. coli* (MW-test: *p* < 0.05, KS-test: *p* < 0.01). For *Yersinia* and *Vibrio*, the difference was statistically much weaker (MW-test: *p* < 0.2, KS-test: *p* < 0.01).

Mean Sequence Identity (%)

|  | Mean Sequence Identity (%) |
| --- | --- |
| Ancient | 85.2 |
| All | 84.5 |
| Young | 78.8 |

**Figure S18. Sequence Divergence and Fold Age**

SCOP folds in human (x-axis) were compared to orthologs in mouse and the mean sequence identity (y-axis) was recorded (see Methods). In all comparisons, ancient folds (see Methods), were found to be significantly more sequence conserved than young folds found only in human and mouse (MW-test, KS-test: *p* < 0.01).

Mean Occurrence

| Scop Level | Ancient | All | Young |
| --- | --- | --- | --- |
| Family | 17.1 | 24.3 | 24.1 |
| Fold | 60.7 | 52.1 | 36.2 |

**Figure S19. SCOP domain occurrence and Fold Age**

The occurrence of ancient SCOP domains (see Methods) in the human proteome was compared to that of young domains (appearing only in human and mouse). Ancient families were significantly less abundant than young families (MW-test, KS-test: *p* < 0.01). At the fold level, no statistical difference in occurrence could be established between ancient and young folds.

**Figure S20. SCOP domain duplication and Fold Age**

Mean Duplication

| Scop Level | Ancient | All | Young |
| --- | --- | --- | --- |
| Family | 1.3 | 1.953 | 2.8 |
| Fold | 2.9 | 2.7 | 3.2 |

The number of times ancient SCOP domains (see Methods) were duplicated in the same human protein was compared to that of young domains (appearing only in human and mouse). Ancient families were significantly less duplicated than young families (MW-test: *p* < 0.05, KS-test: *p* < 0.01). At the fold level, no statistical difference in duplication could be established between ancient and young folds.

Mean Promiscuity

| Scop Level | Ancient | All | Young |
| --- | --- | --- | --- |
| Family | 2.75 | 3.38 | 3.758 |
| Fold | 6 | 5.3 | 3.6 |

**Figure S21. SCOP domain promiscuity and Fold Age**

The promiscuity of ancient SCOP domains (see Methods) in the human proteome was compared to that of young domains (appearing only in human and mouse). Ancient families were significantly less promiscuous than young families (MW-test: *p* < 0.1, KS-test: *p* < 0.01). At the fold level, ancient folds were more promiscuous than young folds (MW-test: *p* < 0.07, KS-test: *p* < 0.01).

Length (aa)

| Number of Families | Column C |
| --- | --- |
| 1 | 85 |
| 1 | 248 |
| 1 | 146.969 |
| 1 | 101 |
| 1 | 196.4 |
| 1 | 104.5 |
| 1 | 202.5 |
| 1 | 240 |
| 1 | 305.727 |
| 1 | 130 |
| 1 | 169 |
| 1 | 36 |
| 1 | 106 |
| 1 | 173.4 |
| 1 | 94 |
| 1 | 278 |
| 1 | 79.9375 |
| 1 | 129.25 |
| 1 | 157.385 |
| 1 | 136.25 |
| 1 | 742 |
| 1 | 58 |
| 1 | 113 |
| 1 | 59.4286 |
| 1 | 217.047 |
| 1 | 183.357 |
| 1 | 122.675 |
| 1 | 85.8955 |
| 1 | 491.333 |
| 1 | 72 |
| 1 | 207.75 |
| 1 | 325.2 |
| 1 | 98.7083 |
| 1 | 160.111 |
| 1 | 138.5 |
| 1 | 175 |
| 1 | 205.2 |
| 1 | 119 |
| 1 | 77 |
| 1 | 286.5 |
| 1 | 144.25 |
| 1 | 257 |
| 1 | 196.333 |
| 2 | 191 |
| 2 | 75 |
| 2 | 148.5 |
| 2 | 177.7 |
| 2 | 109.6 |
| 2 | 147 |
| 2 | 45 |
| 2 | 267.269 |
| 2 | 397.083 |
| 2 | 362 |
| 2 | 174.739 |
| 2 | 118.431 |
| 2 | 68.5 |
| 2 | 156 |
| 2 | 187.857 |
| 2 | 118.8 |
| 2 | 91.8889 |
| 2 | 221.957 |
| 2 | 194.815 |
| 2 | 160 |
| 2 | 239.913 |
| 2 | 132.333 |
| 2 | 241.5 |
| 2 | 101 |
| 2 | 169.667 |
| 2 | 306.933 |
| 2 | 65.1818 |
| 2 | 333 |
| 2 | 69 |
| 2 | 52 |
| 2 | 209.667 |
| 2 | 146.462 |
| 2 | 294.4 |
| 2 | 217.786 |
| 2 | 171.5 |
| 2 | 110 |
| 2 | 118.769 |
| 2 | 271.333 |
| 2 | 113.667 |
| 2 | 68 |
| 2 | 229.5 |
| 2 | 238.25 |
| 2 | 217 |
| 2 | 99.4737 |
| 2 | 90 |
| 2 | 328.938 |
| 3 | 101.366 |
| 3 | 231.929 |
| 3 | 72.6667 |
| 3 | 434.286 |
| 3 | 194 |
| 3 | 134.143 |
| 3 | 197.333 |
| 3 | 155.333 |
| 3 | 100.833 |
| 3 | 392 |
| 3 | 54.5 |
| 3 | 250.63 |
| 3 | 205.667 |
| 3 | 94.5 |
| 3 | 141.727 |
| 3 | 113.4 |
| 3 | 361 |
| 3 | 145.5 |
| 3 | 103.912 |
| 3 | 187 |
| 4 | 91.6667 |
| 4 | 168.333 |
| 4 | 111.417 |
| 4 | 86.0667 |
| 4 | 212.365 |
| 4 | 101.667 |
| 4 | 170.5 |
| 4 | 181.714 |
| 4 | 129.63 |
| 4 | 179.045 |
| 4 | 68.0909 |
| 4 | 194 |
| 4 | 94.6304 |
| 4 | 138 |
| 4 | 205.458 |
| 4 | 95 |
| 4 | 71.25 |
| 4 | 313.55 |
| 4 | 526 |
| 4 | 235.5 |
| 4 | 93.9474 |
| 4 | 204.8 |
| 4 | 119.333 |
| 4 | 217 |
| 4 | 144.125 |
| 4 | 179.5 |
| 4 | 92.1429 |
| 5 | 118.727 |
| 5 | 202.7 |
| 5 | 112.743 |
| 5 | 118 |
| 5 | 252 |
| 5 | 93.9703 |
| 5 | 146.167 |
| 5 | 117 |
| 5 | 76 |
| 5 | 176 |
| 5 | 71.5 |
| 5 | 99 |
| 5 | 89.8571 |
| 5 | 149 |
| 5 | 56 |
| 5 | 252.917 |
| 5 | 77.5417 |
| 5 | 63.1818 |
| 5 | 98 |
| 5 | 117.333 |
| 5 | 158.667 |
| 5 | 60.8 |
| 5 | 109 |
| 5 | 112 |
| 5 | 143.667 |
| 5 | 130.462 |
| 5 | 259 |
| 5 | 75 |
| 5 | 133.5 |
| 5 | 229 |
| 5 | 123 |
| 5 | 145.714 |
| 5 | 155.692 |
| 5 | 261.333 |
| 5 | 115.8 |
| 5 | 319 |
| 5 | 267 |
| 5 | 190.5 |
| 6 | 155.079 |
| 6 | 398.333 |
| 6 | 192.375 |
| 6 | 259.665 |
| 6 | 198.5 |
| 6 | 127 |
| 6 | 57 |
| 6 | 220 |
| 6 | 73 |
| 6 | 271.95 |
| 6 | 223.7 |
| 6 | 86.6667 |
| 6 | 105.636 |
| 6 | 187 |
| 6 | 101.2 |
| 6 | 109 |
| 7 | 93.3333 |
| 7 | 71 |
| 7 | 221.4 |
| 7 | 134 |
| 7 | 177.5 |
| 7 | 119.5 |
| 7 | 94.8333 |
| 7 | 242.917 |
| 7 | 111.25 |
| 7 | 96 |

**Figure S22. Length and Number of Families**

The number of families in ancient SCOP folds detected in human proteins were plotted against their length. Little correlation (R^2^ = -0.1) was found. A similar relation with little correlation between length and family counts was found when all human folds were included in the analysis (data not shown).

Number of Proteins

| Number of Families | All Alpha | All Beta | Alpha/Beta | Alpha + Beta | Other |
| --- | --- | --- | --- | --- | --- |
| 1 | 14.2 | 17.1 | 8.6 | 16.4 | 20.9 |
| >1 | 19 | 36.2 | 18.1 | 24.3 | 53.9 |
| >5 | 19 | 38.2 | 21.8 | 28.5 | 26.8 |
| >10 | 23.8 | 44 | 24 | 19.4 | 28.8 |

**Figure S23. Occurrence of Human Families and Fold Class**

SCOP folds were divided into a number of bins according to the number of families that they contain (x-axis). For each bin, the mean family occurrence (the mean number of human proteins, families in the SCOP folds appear in) is shown, divided amongst 5 SCOP fold classes. In general, as the number of families in a SCOP fold increases, the occurrence of these families in the human proteome tends to increase. A notable exception to this trend is shown for folds classified as Other. In this class, the C2H2, Ring finger and the Rhodopsin-like families occupy over 500, 400 and 300 proteins respectively but belong to folds with only 2-3 known families.

Mean Number of Organisms

| Number of Families | Folds | Families |
| --- | --- | --- |
| 1 | 3.35 | 3.34 |
| >1 | 6.45 | 3.59 |
| >5 | 8.44 | 3.43 |
| >10 | 8.78 | 3.37 |

**Figure S24. Occurrence of Folds and Families Across Genomes**

SCOP folds and families were searched for (see Methods) in proteins predicted to occur in the following genomes: *Bacteroides fragilis NCTC9434, Mus musculus, Deinococcus geothermalis, Escherichia coli K12, Vibrio fischeri ES114, Psychrobacter arcticum 273 4, Chlorobium vibrioforme, Sulfolobus acidocaldarius DSM, Saccharomyces cerevisiae, Mycoplasma hyopneumoniae 232, Streptococcus zooepidemicus, and Anabaena variabilis.* Folds were divided into a number of bins according to the number of families that they contain (x-axis). The mean number of organisms the folds (white bar) or families (black bar) appear in is plotted for each bin. Folds containing greater number of families tend to occur more often across organisms. Mean genome occurrences between folds with one and more than family were significantly different (MW-test, KS-test: *p* < 0.01). However, individual families belonging to folds with greater number of families tend not to occur more often across these organisms.

|  | yeast - ecoli | mouse - ecoli | human - ecoli | mouse - yeast | human - yeast | human - mouse |
| --- | --- | --- | --- | --- | --- | --- |
| 1 | c.37 | b.1 | b.1 | b.1 | b.1 | b.1 |
| 2 | a.118 | c.37 | c.37 | c.37 | d.144 | d.144 |
| 3 | b.69 | a.4 | a.4 | d.144 | c.37 | c.37 |
| 4 | b.34 | d.58 | b.34 | a.4 | a.4 | g.37 |
| 5 | d.153 | c.2 | b.69 | c.2 | d.58 | b.34 |
| 6 | g.41 | b.69 | d.58 | d.58 | b.34 | a.4 |
| 7 | c.55 | a.118 | a.118 | c.10 | d.211 | b.55 |
| 8 | b.43 | d.81 | b.36 | d.81 | c.10 | b.69 |
| 9 | b.80 | b.34 | b.47 | d.211 | b.69 | d.211 |
| 10 | a.2 | b.47 | b.7 | b.69 | b.55 | g.3 |

**Protocol S1: Top Ten Fold Occurrence Differences between Organism Pairs**

The 10 folds with the largest fold occurrence differences for each organism pair are listed in each column. Comparing the last 3 columns: fold occurrence differences between yeast and the two mammals is similar to fold occurrence differences between human and mouse. Comparing the first 3 columns: fold occurrence differences between *E. coli* and each of the three eukaryotes is less similar. The c.37 (P-loop containing nucleoside triphosphate hydrolases) and the b.69 (7-bladed beta-propeller) fold have consistently exhibited large occurrence differences between organism pairs. Other notable folds include b.1, d.58, and a.4, all of which are predicted to be highly designable.

**Protocol S2: Disease Proteins containing folds with 1 family**

In this and previous work [7], it was found that disease proteins have significantly higher number of proteins containing folds with 1 family as compared with non-disease proteins. We list these proteins here, along with their folds and associated diseases as annotated in OMIM. The list contains 228 proteins and 106 different folds associated with a diversity of diseases. 15 and 10 proteins respectively are annotated with complement system and leukemia related diseases and form the largest grouping of proteins related by disease annotation. Even after removing these proteins, disease proteins were predicted to be significantly less designable than non-disease proteins by their fold family counts (MW-test: *p* < 0.05, KS-test: *p* < 0.01; data not shown). An * in the fold column indicates that the SCOP definition highly covers the protein (see Methods). The folds below were also scanned across genomes (Supplementary Material S24). Most of the folds (~2/3) were detected only in human, mouse and/or yeast suggesting that they are eukaryotic in origin. However, (~1/3) were detected in 3 other prokaryotes and (~1/4) of the folds below present in over 7 genomes (see Methods). These latter folds are relatively ancient and the absence of many families in these folds suggests that they are relatively less designable. Disease proteins were predicted to be significantly less designable than non-disease proteins by their fold family counts even if we excluded the young folds predicted to be eukaryotic in origin (MW-test: *p* < 0.1, KS-test: *p* < 0.01; data not shown).

ENSEMBL Protein	Folds	Associated OMIM Disease Annotation

ensp00000226359	a.126	[AFP deficiency congenital] (1),[Hereditary persistence of alpha-fetoprotein] (3)ensp00000261779	g.18	[Anhaptoglobinemia] (3),[Hypohaptogloginemia] (3)

ensp00000205948	g.18,	[Apolipoprotein H deficiency] (3)

ensp00000200676	d.83	[CETP deficiency] (3)

ensp00000329374	e.1*	[Euthyroidal hyper- and hypothyroxinemia] (1)

ensp00000342850	e.1*	[Transcortin deficiency] (1)

ensp00000289416	e.23	{?Hypertension essential} (1)

ensp00000224541	d.9	{AIDS resistance to} (3)

ensp00000331736	g.18,	{Atherosclerosis susceptibility to} (2)

ensp00000302692	d.19*	{Beryllium disease chronic susceptibility to} (3)

ensp00000234420	a.113	{Cancer susceptibility} (3)

ensp00000258224	e.1	{Hypertension essential susceptibility to} (3),{Preeclampsia susceptibility to} (3)

ensp00000313875	g.18,	{Measles susceptibility to} (1)

ensp00000335243	d.19*	{Pemphigoid susceptibility to} (2),Diabetes mellitus insulin-dependent-1 (2) (?)

ensp00000304123	b.22	{Systemic lupus erythematosus susceptibility} 152700 (3)

ensp00000218425	a.87	Aarskog-Scott syndrome (3)

ensp00000265517	f.7	Abetalipoproteinemia 200100 (3)

ensp00000233242	f.7	Abetalipoproteinemia (3),Apolipoprotein B-100 ligand-defective (3),

Hyperbetalipoproteinemia (3),Hypobetalipoproteinemia (3)

ensp00000241052	e.5*	Acatalasemia (3)

ensp00000292427	a.104	Adrenal hyperplasia congenital due to 11-beta-hydroxylase deficiency (3)

Aldosteronism glucocorticoid-remediable (3)

ensp00000278017	a.104*	Adrenal hyperplasia congenital due to 17-alpha-hydroxylase deficiency (3)

ensp00000303875	a.104*	Adrenal hyperplasia congenital due to 21-hydroxylase deficiency (3)

Carbamoylphosphate synthetase I deficiency (3)

ensp00000302390	a.123	Adrenal hypoplasia congenital with hypogonadotropic hypogonadism (3)

ensp00000311469	a.45	Agammaglobulinemia 601495 (3)

ensp00000337233	d.93	Agammaglobulinemia type 1 X-linked (3)

XLA and isolated growth hormone deficiency 307200 (3) (?)

ensp00000261981	e.1*	Alpha-1-antichymotrypsin deficiency (3),Cerebrovascular disease occlusive (3)

ensp00000306361	d.171	Amyloidosis hereditary renal 105200 (3)

Dysfibrinogenemia alpha type causing bleeding diathesis (3)

Dysfibrinogenemia alpha type causing recurrent thrombosis (3)

ensp00000295897	a.126	Analbuminemia (3),[Dysalbuminemic hyperthyroxinemia] (3)

[Dysalbuminemic hyperzincemia] 194470 (3)

ensp00000313925	a.123	Androgen insensitivity several forms (3),Breast cancer male with Reifenstein syndrome (3),	Perineal hypospadias (3),Prostate cancer (3),

Spinal and bulbar muscular atrophy of Kennedy 313200 (3)

ensp00000271945	b.58	Anemia hemolytic due to PK deficiency (3)

ensp00000343033	c.71*	Anemia megaloblastic due to DHFR deficiency (1) (?)

ensp00000345138	c.71	Anemia megaloblastic due to DHFR deficiency (1) (?)

ensp00000200691	g.46*	Anemia pernicious congenital due to deficiency of intrinsic factor (1)

ensp00000232165	d.148	Angelman syndrome (3),Ankylosing spondylitis (2)

ensp00000278407	e.1	Angioedema hereditary (3)

ensp00000289824	b.22	Anhidrotic ectodermal dysplasia (2)

ensp00000296507	a.38	Anhidrotic ectodermal dysplasia (2),Ectodermal dysplasia hidrotic (2)

ensp00000236260	e.1*	Antithrombin III deficiency (3)

ensp00000327433	f.37	Bare lymphocyte syndrome type I due to TAP2 deficiency (1)

ensp00000274376	d.93	Basal cell carcinoma (3)

ensp00000286548	a.66	Bleeding diathesis due to GNAQ deficiency (1)

ensp00000242261	a.38	Blepharophimosis epicanthus inversus and ptosis type 2 (2),

Saethre-Chotzen syndrome 101400 (3)

ensp00000206249	a.123	Breast cancer (1),Estrogen resistance (3)

ensp00000267071	a.170,a.171	Breast cancer 2 early onset (3),Pancreatic cancer (3),Fanconi anemia type D (2)

ensp00000315969	f.33,d.220	Brody myopathy 601003 (3)

ensp00000259523	a.38	Burkitt lymphoma (3)

ensp00000313967	b.22	C1q deficiency type B (3)

ensp00000290301	b.22	C1q deficiency type C (3)

ensp00000290575	g.18	C1r/C1s deficiency combined (1)

ensp00000328173	g.18	C1r/C1s deficiency combined (1)

ensp00000299367	g.18	C2 deficiency (3)

ensp00000245907	a.50	C3 deficiency (3)

ensp00000265172	g.12	C3b inactivator deficiency (3)

ensp00000299406	a.50	C4 deficiency (3)

ensp00000223642	a.50	C5 deficiency (1)

ensp00000338861	g.60,g.12	C6 deficiency (1),Combined C6/C7 deficiency (1)

ensp00000322061	g.60,g.18,g.12	C7 deficiency (1)

ensp00000305651	d.9*	C7 deficiency (1)

ensp00000287797	g.60,g.12	C8 deficiency type I (2)

ensp00000234643	g.60,g.12	C8 deficiency type II (3)

ensp00000263408	g.12	C9 deficiency (3)

ensp00000245479	a.21	Campomelic dysplasia with autosomal sex reversal (3),Cardiomyopathy familial dilated 1B (2)

ensp00000233072	a.92	Carbamoylphosphate synthetase I deficiency (3)

ensp00000316712	d.93	Cardiofaciocutaneous syndrome 115150 (2),Noonan syndrome-1 (2)

ensp00000341922	a.173	Cataract cerulean type 1 (2)

ensp00000258415	a.104	Cerebrotendinous xanthomatosis (3)

ensp00000272000	a.169	Chediak-Higashi syndrome (3)

ensp00000265723	f.37	Cholestasis progressive familial intrahepatic type III 602347 (3)

ensp00000333122	a.123	Chondrosarcoma extraskeletal myxoid (1)

ensp00000289473	d.189	Chronic granulomatous disease due to deficiency of NCF-1 (3)

ensp00000342903	d.210	Citrullinemia (3)

ensp00000325822	a.104	CMO II deficiency (3)

ensp00000265724	f.37	Colchicine resistance (3)

ensp00000269305	a.53	Colorectal cancer 114500 (3),Li-Fraumeni syndrome (3)

ensp00000233146	a.113	Colorectal cancer hereditary nonpolyposis type 1 (3) Ovarian cancer (3),Muir-Torre syndrome 158320 (3)

ensp00000343888	a.21	Colorectal cancer hereditary nonpolyposis type 3 (3)

ensp00000311165	f.19	Colton blood group 110450 (3)

ensp00000251047	a.21	Combined factor V and VIII deficiency 227300 (3)

ensp00000181839	e.32	Congenital hereditary endothelial dystrophy of cornea (2)

ensp00000262464	g.23	Contractural arachnodactyly congenital (3)

ensp00000306306	b.118	Corneal dystrophy Avellino type (3),Corneal dystrophy Groenouw type I 121900 (3),

Corneal dystrophy lattice type I 122200 (3),Reis-Bucklers corneal dystrophy (3)

ensp00000305913	b.22	Corneal dystrophy posterior polymorphous (2)

ensp00000231509	a.123	Cortisol resistance (3)

ensp00000301141	a.104*	Coumarin resistance 122700 (3),{Nicotine addiction protection from} (3)

ensp00000263045	d.111*	Craniosynostosis Adelaide type (2)

ensp00000339996	d.6	Creutzfeldt-Jakob disease 123400 (3),Gerstmann-Straussler disease 137440 (3),

Insomnia fatal familial (3),5-oxoprolinuria 266130 (3),

Hemolytic anemia due to glutathione synthetase deficiency 231900 (3)

ensp00000222399	a.114	Cutis laxa marfanoid neonatal type (1) (?)

ensp00000231004	a.80	Cutis laxa recessive type I 219100 (1)

ensp00000256216	d.106	D-bifunctional protein deficiency (3)

ensp00000324892	f.33,d.220	Darier disease (keratosis follicularis) (2)

ensp00000329773	f.14	Deafness autosomal dominant 2 (2)

ensp00000216361	d.209	Deafness autosomal dominant 9 (2)

ensp00000291532	g.12	Deafness autosomal recessive 8 (2)

ensp00000248928	a.104*	Debrisoquine sensitivity (3),{?Parkinsonism susceptibility to} (1)

ensp00000223955	g.62	Dementia familial nonspecific (2)

ensp00000304257	f.20	Dent disease 300009 (3),Hypophosphatemia type III (3),Nephrolithiasis type I 310468 (3),

Proteinuria low molecular weight with hypercalciuric nephrocalcinosis (3)

ensp00000199280	f.19*	Diabetes insipidus nephrogenic autosomal recessive 222000 (3)

ensp00000217172	b.9*	Diabetes insipidus neurohypophyseal 125700 (3)

ensp00000250971	g.1*	Diabetes mellitus rare form (1),Hyperproinsulinemia familial (3),MODY one form (3)

ensp00000263196	g.12	DiGeorge syndrome/velocardiofacial syndrome complex-2 (2)

ensp00000266063	f.37	Dubin-Johnson syndrome 237500 (3)

ensp00000306099	d.171	Dysfibrinogenemia beta type (3)

ensp00000336829	d.171	Dysfibrinogenemia gamma type (3),Hypofibrinogenemia gamma type (3)

ensp00000308541	g.32	Dysprothrombinemia (3),Hypoprothrombinemia (3)

ensp00000325527	g.23	Ectopia lentis ?isolated (3),Marfan syndrome 154700 (3),

Shprintzen-Goldberg syndrome 182212 (3)

ensp00000323534	g.27	Ehlers-Danlos syndrome type X (1) (?)

ensp00000298844	e.1*	Emphysema (3),Emphysema-cirrhosis (3),

Hemorrhagic diathesis due to `antithrombin' Pittsburgh (3)

ensp00000265081	a.113	Endometrial carcinoma (3)

ensp00000234590	d.54	Enolase deficiency (1)

ensp00000284885	g.12	Enterokinase deficiency (1)

ensp00000342552	f.14	Epilepsy benign neonatal type 1 121200 (3),

Epilepsy benign neonatal type 1 121200 (3),Epilepsy nocturnal frontal lobe 600513 (3)

ensp00000217176	b.96,f.36	Epilepsy benign neonatal type 1 121200 (3),Epilepsy nocturnal frontal lobe 600513 (3),	Epilepsy benign neonatal type 1 121200 (3)

ensp00000330043	f.14	Epilepsy benign neonatal type 2 121201 (3)

ensp00000228858	f.14	Episodic ataxia/myokymia syndrome 160120 (3)

ensp00000001567	g.18,	Factor H deficiency (1),Hemolytic-uremic syndrome 235400 (3),

Membroproliferative glomerulonephritis (1)

ensp00000261967	g.32	Factor VII deficiency (3)

ensp00000246496	g.32	Factor X deficiency (3)

ensp00000271509	g.18,	Factor XIIIB deficiency (3)

ensp00000272146	a.41	Fanconi anemia (1) (?),Xeroderma pigmentosum (1) (?)

ensp00000263879	a.117,a.87	Fibromatosis gingival (2)

ensp00000345039	d.51	Fragile X syndrome (3)

ensp00000262017	d.200	Glanzmann thrombasthenia type B (3)

ensp00000260630	a.104*	Glaucoma 3A primary infantile 231300 (3)

ensp00000282439	c.89	Glycogen storage disease VII (3)

ensp00000297137	b.95*	GM2-gangliosidosis AB variant (3)

ensp00000220616	g.28,	Goiter adolescent multinodular (1),Goiter nonendemic simple (3),

Hypothyroidism hereditary congenital (3)

ensp00000329869	g.18	Goiter congenital (3),Hypothyroidism congenital (3),

Thrombocythemia essential 187950 (3),Thyroid iodine peroxidase deficiency (1)

ensp00000329362	a.21*	Gonadal dysgenesis XY type (3)

ensp00000302665	g.1	Growth retardation with deafness and mental retardation (3)

ensp00000260433	a.104	Gynecomastia familial due to increased aromatase activity (1),

Virilization maternal and fetal from placental aromatase deficiency (3)

ensp00000311698	d.19*	Hemochromatosis (3)

ensp00000328511	f.14	Hemolytic anemia due to hexokinase deficiency (3)

ensp00000218265	c.86*	Hemolytic anemia due to PGK deficiency (3),

Myoglobinuria/hemolysis due to PGK deficiency (3)

ensp00000309438	c.89,c.89	Hemolytic anemia due to phosphofructokinase deficiency (1)

ensp00000218099	g.32	Hemophilia B (3)

ensp00000223095	e.1*	Hemorrhagic diathesis due to PAI1 deficiency (1),

Thrombophilia due to excessive plasminogen activator inhibitor (1)

ensp00000343606	b.64	Hepatocellular carcinoma (3)

ensp00000344460	c.79	Homocystinuria B6-responsive and nonresponsive types (3)

ensp00000252444	g.12,	Hypercholesterolemia familial (3)

ensp00000274576	b.96	Hyperekplexia and spastic paraparesis (3),Startle disease autosomal recessive (3),

Startle disease/hyperekplexia autosomal dominant 149400 (3)

ensp00000261192	e.17	Hyperleucinemia-isoleucinemia or hypervalinemia (1) (?)

ensp00000280867	d.130	Hypermethioninemia persistent autosomal dominant due to

methionine adenosyltransferase I/III deficiency (3)

ensp00000297494	d.174	Hypertension pregnancy-induced (2)

ensp00000265074	c.93	Hypertension salt-resistant (1) (?)

ensp00000322991	e.17*	Hypervalinemia or hyperleucine-isoleucinemia (1) (?)

ensp00000340482	c.93	Hypocalcemia autosomal dominant 601198 (3),

Hypocalciuric hypercalcemia type I 145980 (3),Neonatal hyperparathyroidism 239200 (3)

ensp00000319814	c.109	Hypoglycemia due to PCK1 deficiency (1) (?)

ensp00000218368	b.22	Immunodeficiency X-linked with hyper-IgM (3)

ensp00000257555	a.34	Insulin-dependent diabetes mellitus (3),

MODY type 3 600496 (3),Non-insulin-dependent diabetes mellitus-2 601407 (2)

ensp00000251819	b.66,g.64	Jacobs syndrome (2)

ensp00000155840	f.14	Jervell and Lange-Nielsen syndrome 220400 (3),Long QT syndrome-1 (3)

ensp00000227868	a.9	Lacticacidemia due to PDX1 deficiency (3)

ensp00000264932	d.168	Leigh syndrome (3)

ensp00000262965	a.38	Leukemia acute lymphoblastic (1)

ensp00000282111	a.21	Leukemia acute lymphoblastic (1)

ensp00000254066	a.123	Leukemia acute promyelocytic (1)

ensp00000303507	a.87,a.147	Leukemia chronic myeloid (3)

ensp00000298467	d.93	Leukemia chronic myeloid (3)

ensp00000306443	a.154	Leukemia myeloid/lymphoid or mixed-lineage (2)

ensp00000277541	g.65	Leukemia T-cell acute lymphoblastic (2)

ensp00000264824	a.38	Leukemia T-cell acute lymphoblastoid (2)

ensp00000294339	a.38	Leukemia-1 T-cell acute lymphoblastic (3)

ensp00000216612	b.63*	Leukemia/lymphoma T-cell (2)

ensp00000331181	d.93*	Lymphoproliferative syndrome X-linked (2),{?Amyloidosis secondary susceptibility to} (1)

ensp00000343136	a.9	Maple syrup urine disease type II (3)

ensp00000261978	g.23	Marfan syndrome atypical (3)

ensp00000301873	g.23	Marfan syndrome atypical (3)

ensp00000302237	a.66	McCune-Albright polyostotic fibrous dysplasia 174800 (3),

Pituitary ACTH secreting adenoma (3),

Pseudohypoparathyroidism type Ia 103580 (3),Somatotrophinoma (3)

ensp00000285981	a.104*	Mephenytoin poor metabolizer (3)

ensp00000285979	a.104*	Mephenytoin poor metabolizer (3)

ensp00000243222	b.22	Metaphyseal chondrodysplasia Schmid type (3)

ensp00000341625	d.120*	Methemoglobinemia due to cytochrome b5 deficiency (3)

ensp00000343807	a.123	MODY type 1 125850 (3),Non-insulin-dependent diabetes mellitus 125853 (3)

ensp00000225893	a.34	MODY type 5 (3)

ensp00000258391	f.36,b.96	Myasthenia gravis neonatal transient (2)

ensp00000304290	f.36,b.96	Myasthenic syndrome slow-channel congenital 601462 (3)

ensp00000261007	f.36,b.96	Myasthenic syndrome slow-channel congenital 601462 (3)

ensp00000293780	f.36	Myasthenic syndrome slow-channel congenital 601462 (3)

ensp00000290858	b.54*	Myeloid leukemia acute M4Eo subtype (2)

ensp00000303844	g.40,g.40	Myotonic dystrophy 2 (2)

ensp00000314415	g.67	N syndrome 310465 (1) (?)

ensp00000255622	a.125	Night blindness congenital stationary type 3 163500 (3),Retinitis pigmentosa autosomal recessive (3)

ensp00000232461	a.66	Night blindness congenital stationary (3)

ensp00000334876	a.91*	Oguchi disease-2 258100 (3)

ensp00000229022	a.123	Osteoporosis involutional (1) (?),Rickets vitamin D-resistant 277440 (3)

ensp00000294304	g.12	Osteoporosis-pseudoglioma syndrome (2),[High bone mass] (2)

ensp00000338276	d.111*	Pancreatic cancer (2)

ensp00000341551	d.164	Pancreatic cancer (3)

ensp00000303960	f.37	Persistent hyperinsulinemic hypoglycemia of infancy 256450 (3)

ensp00000303500	d.178	Phenylketonuria (3),[Hyperphenylalaninemia mild] (3)

ensp00000312999	a.66	Pituitary ACTH-secreting adenoma (3)

ensp00000321853	e.1	Plasmin inhibitor deficiency (3)

ensp00000220809	g.27	Plasminogen activator deficiency (1)

ensp00000263686	g.18	Platelet alpha/delta storage pool deficiency (1)

ensp00000268053	a.104	Polycystic ovary syndrome with hyperandrogenemia (2)

ensp00000339185	c.113*	Porphyria congenital erythropoietic (3)

ensp00000263817	f.37	Progressive intrahepatic cholestasis-2 (2)

ensp00000244137	d.127	Prolidase deficiency (3)

ensp00000247153	g.60	Properdin deficiency X-linked (3)

ensp00000331152	a.38	Prostate cancer 176807 (3)

ensp00000298842	e.1*	Protein C inhibitor deficiency (2)

ensp00000330021	g.32	Protein S deficiency (3)

ensp00000228606	a.104	Pseudo-vitamin D dependency rickets 1 (2)

ensp00000281330	a.123	Pseudohypoaldosteronism type I autosomal dominant 177735 (3)

ensp00000234071	g.32	Purpura fulminans neonatal (1),Thrombophilia due to protein C deficiency (3)

ensp00000285379	b.74*	Renal tubular acidosis-osteopetrosis syndrome (3)

ensp00000255266	a.125	Retinitis pigmentosa autosomal recessive (3)

ensp00000324122	a.183	Retinitis pigmentosa-11 (2)

ensp00000229771	d.23	Retinitis pigmentosa-14 600132 (3)

ensp00000235382	a.91	Rieger syndrome type 2 (2)

ensp00000262367	g.53,a.12,a.153	Rubenstein-Taybi syndrome 180849 (3),Colorblindness protan (3),

Rett syndrome (2) (?),Rothmund-Thomson syndrome (2) (?)

ensp00000320324	b.98	Salivary gland pleomorphic adenoma (2)

ensp00000303727	f.36,b.96	Schizophrenia neurophysiologic defect in (2)

ensp00000325831	d.178	Segawa syndrome recessive (3)

ensp00000264972	d.93	Selective T-cell defect (3)

ensp00000286628	f.14	Smith-Lemli-Opitz syndrome (2)

ensp00000337994	a.114	Speech-language disorder-1 (2)

ensp00000305769	d.164	Spinal muscular atrophy distal with upper limb predominance (2)

ensp00000317862	a.68	Thrombocytopenia X-linked 313900 (3),Wiskott-Aldrich syndrome (3)

ensp00000215727	e.1	Thrombophilia due to heparin cofactor II deficiency (3)

ensp00000338087	a.104*	Thromboxane synthase deficiency (2)

ensp00000280696	a.123	Thyroid hormone resistance 274300 188570 (3)

ensp00000295600	a.38	Tietz syndrome 103500 (3),Waardenburg syndrome type IIA 193510 (3),

Waardenburg syndrome/ocular albinism digenic 103470 (3)

ensp00000260682	a.104*	Tolbutamide poor metabolizer (3)

ensp00000258070	d.93	Tylosis with esophageal cancer (2)

ensp00000261755	d.177	Tyrosinemia type I (3)

ensp00000215964	a.21	Waardenburg-Shah syndrome 277580 (3)

ensp00000289041	d.133,a.56	Xanthinuria type I (3)

**Protocol S3: List of Common Disease Proteins**

Common disease proteins with detectable SCOP folds are listed here. An * in the fold column indicates that the SCOP definition highly covers the protein (see Methods).

ENSEMBL Protein	Folds	Associated OMIM Disease Annotation

ensp00000217961	c.76	Ichthyosis X-linked (3),Placental steroid sulfatase deficiency (3)

ensp00000218195	f.13*	Colorblindness blue/protan (3),Rubenstein-Taybi syndrome 180849 (3)

ensp00000229264	b.69*	{Hypertension essential susceptibility to} 145500 (3)

ensp00000233242	a.118,f.7	Abetalipoproteinemia (3),Apolipoprotein B-100 ligand-defective (3),Hyperbetalipoproteinemia	(3),Hypobetalipoproteinemia (3)

ensp00000236260	e.1*	Antithrombin III deficiency (3)

ensp00000246337	c.1*	Porphyria cutanea tarda (3),Porphyria hepatoerythropoietic (3)

ensp00000249389	f.13*	Colorblindness tritan (3)

ensp00000252444	b.68,g.3,g.12	Hypercholesterolemia familial(3)

ensp00000256993	b.1	Cardiomyopathy familial hypertrophic 4 115197 (3),Hypercholesterolemia familial (3)

ensp00000260682	a.104*	Tolbutamide poor metabolizer (3)

ensp00000261733	c.82*	Alcohol intolerance acute (3),{?Fetal alcohol syndrome} (1)

ensp00000262367	g.53,a.12,a.29,a.153	Rubenstein-Taybi syndrome 180849 (3),Colorblindness protan (3),Rett syndrome	(2) (?),Rothmund-Thomson syndrome (2) (?)

ensp00000264381	c.69	Apnea postanesthetic (3)

ensp00000264691	b.47	Factor XI deficiency (3)

ensp00000267071	a.170,b.40,a.171,b.40	Breastcancer 2 early onset (3),Pancreatic cancer (3),

Fanconi anemia type D (2)

ensp00000276343	f.13*	Colorblindness blue monochromatic (3),Colorblindness deutan (3)

ensp00000276348	f.13*	Colorblindness blue monochromatic (3),Colorblindness deutan (3)

ensp00000289928	b.1	Charcot-Marie-Tooth neuropathy-1B 118200 (3),

Dejerine-Sottas disease myelin P(0)-related 145900 (3),Hypomyelination congenital (3)

ensp00000292301	f.13*	{HIV infection susceptibility/resistence to} (3)

ensp00000292303	f.13*	{HIV infection susceptibility/resistance to} (3)

ensp00000292888	b.1	Nephrosis-1 congenital Finnish type 256300 (3)

ensp00000298844	e.1*	Emphysema (3),Emphysema-cirrhosis (3),

Hemorrhagic diathesis due to `antithrombin' Pittsburgh (3)

ensp00000299367	b.47,g.18	C2 deficiency (3)

ensp00000301141	a.104*	Coumarin resistance 122700 (3),{Nicotine addiction protection from} (3)

ensp00000306780	f.13.1,c.10	Ovarian dysgenesis hypergonadotropic with normal karyotype 233300 (3)

ensp00000314508	c.1,b.71	Gaucher disease (3),Gaucher disease with cardiovascular calcification (3)

ensp00000331327	g.37	Denys-Drash syndrome (3),Frasier syndrome 136680 (3),Wilms tumor type 1 (3)

ensp00000334146	g.37	Factor XI deficiency (3)

ensp00000340258	b.1	{Atopy susceptibility to} (3)

ensp00000342362	d.81,c.2	Favism (3),G6PD deficiency (3),Hemolytic anemia due to G6PD deficiency (3)

ensp00000343985	f.13*	{HIV infection susceptibility/resistance to} (3)

ensp00000344419	a.93	Myeloperoxidase deficiency (3)

ensp00000344867	f.13	{HIV infection susceptibility/resistence to} (3)

**Protocol S4: List of Rare Disease Proteins**

Rare disease proteins with detectable SCOP folds are listed here. An * in the fold column indicates that the SCOP definition highly covers the protein (see Methods).

ENSEMBL Protein	Folds	Associated OMIM Disease Annotation

ensp00000006951	b.69*	Lissencephaly-1 (3),Miller-Dieker lissencephaly syndrome (2)

ensp00000008553	c.3	[Fish-odor syndrome] 602079 (3)

ensp00000008588	c.76	Mucopolysaccharidosis II (3)

ensp00000039007	c.78*	Ornithine transcarbamylase deficiency (3)

ensp00000053469	a.39*	Cone dystrophy-3 602093 (3)

ensp00000064324	a.4	Iridogoniodysgenesis 601631 (3)

ensp00000164139	c.87*	McArdle disease (3)

ensp00000172196	a.4	Trichodontoosseous syndrome 190320 (3)

ensp00000176643	c.82*	Sjogren-Larsson syndrome (3)

ensp00000196371	c.63	Ketoacidosis due to SCOT deficiency (3)

ensp00000205832	a.127*	Fumarase deficiency (3)

ensp00000206249	a.123,g.39 Breast cancer (1),Estrogen resistance (3)

ensp00000206765	d.3,b.1	Ichthyosiform erythroderma congenital-242100 (3),Ichthyosis lamellar AR 242300 (3)

ensp00000215754	d.80*	Persistent Mullerian duct syndrome type I 261550 (3),

Anemia pernicious congenital due to deficiency of intrinsic factor (1)

ensp00000215964	a.21	Waardenburg-Shah syndrome 277580 (3)

ensp00000215995	c.1,b.71*	Kanzaki disease (3),NAGA deficiency mild (3),Schindler disease (3)

ensp00000216124	c.76 *	Metachromatic leukodystrophy (3)

ensp00000216392	c.87*	Glycogen storage disease VI (3)

ensp00000216727	d.58	Oculopharyngeal muscular dystorphy 164300 (3),

Oculopharyngeal muscular dystrophy autosomal recessive 257950 (3)

ensp00000216951	d.142,c.30 5-oxoprolinuria 266130 (3),

Hemolytic anemia due to glutathione synthetase deficiency 231900 (3)

ensp00000217172	b.9*	Diabetes insipidus neurohypophyseal 125700 (3)

ensp00000218099	b.47,g.3,g.32;g.3	Hemophilia B (3)

ensp00000218265	c.86*	Hemolytic anemia due to PGK deficiency (3),

Myoglobinuria/hemolysis due to PGK deficiency (3)

ensp00000218368	b.22	Immunodeficiency X-linked with hyper-IgM (3)

ensp00000218425	a.87,b.55,g.50	Aarskog-Scott syndrome (3)

ensp00000218516	c.1	Fabry disease (3)

ensp00000220616	c.69,g.28	Goiter adolescent multinodular (1),Goiter nonendemic simple (3),

Hypothyroidism hereditary congenital (3)

ensp00000221496	g.17	Persistent Mullerian duct syndrome type I 261550 (3)

ensp00000221996	a.4	Cone-rod retinal dystrophy-2 120970 (3),Leber congenital amaurosis type III (3)

ensp00000222139	b.1	[Erythrocytosis familial] 133100 (3)

ensp00000222214	e.6,a.29*	Glutaricaciduria type I (3)

ensp00000222246	d.144	SCID autosomal recessive T-negative/B-positive type (3)

ensp00000224242	c.67*	Gyrate atrophy of choroid and retina with ornithinemia B6 responsive or unresponsive (3)

ensp00000225893	a.35,a.4,a.34	MODY type 5 (3)

ensp00000226413	f.13	Hypogonadotropic hypogonadism (3)

ensp00000227157	d.162,c.2	Exertional myoglobinuria due to deficiency of LDH-A (3)

ensp00000227868	c.43,b.84,a.9	Lacticacidemia due to PDX1 deficiency (3)

ensp00000228606	a.104	Pseudo-vitamin D dependency rickets 1 (2)

ensp00000228841	a.39*	Cardiomyopathy hypertrophic mid-left ventricular chamber type (3)

ensp00000229022	a.123,g.39	Osteoporosis involutional (1) (?),Rickets vitamin D-resistant 277440 (3)

ensp00000229270	c.1*	Hemolytic anemia due to triosephosphate isomerase deficiency (3)

ensp00000229725	b.68*	Sialidosis type I (3),Sialidosis type II (3)

ensp00000230732	a.35,a.4	Deafness autosomal dominant 15 602459 (3)

ensp00000230882	b.1	Laron dwarfism 262500 (3),

Short stature autosomal dominant with normal serum growth hormone binding protein (3),

Short stature idiopathic (3)

ensp00000231509	a.123,g.39	Cortisol resistance (3)

ensp00000232461	c.37,a.66	Night blindness congenital stationary (3)

ensp00000233072	d.142,c.23,a.92,c.8,c.30	Carbamoylphosphate synthetase I deficiency (3)

ensp00000233710	e.6,a.29*	Acyl-CoA dehydrogenase long chain deficiency of (3)

ensp00000234071	b.47,g.3,g.32	Purpura fulminans neonatal (1),Thrombophilia due to protein C deficiency (3)

ensp00000234736	b.6.1,b.18	Hemorrhagic diathesis due to factor V deficiency (1),

{Thromboembolism susceptibility due to factor V Leiden} (3)

ensp00000235547	c.2	3-beta-hydroxysteroid dehydrogenase type II deficiency (3)

ensp00000235701	e.6,a.29*	Acyl-CoA dehydrogenase medium chain deficiency of (3)

ensp00000235958	c.1	HMG-CoA lyase deficiency (3)

ensp00000237310	c.42*	Argininemia (3)

ensp00000239243	a.4	Craniosynostosis type 2 (3),Parietal foramina (2)

ensp00000239259	d.144,b.1	Venous malformations multiple cutaneous and mucosal 600195 (3)

ensp00000241052	e.5*	Acatalasemia (3)

ensp00000242261	a.38	Blepharophimosis epicanthus inversus and ptosis type 2 (2),

Saethre-Chotzen syndrome 101400 (3)

ensp00000242480	g.37	Neuropathy congenital hypomyelinating 1 (3)

ensp00000243929	g.3	Hereditary hemorrhagic telangiectasia-2 600376 (3),{Atherosclerosis susceptibility to} (2)

ensp00000244137	d.127	Prolidase deficiency (3)

ensp00000248419	f.13	Bleeding disorder due to defective thromboxane A2 receptor (3)

ensp00000248594	c.45	Colon cancer (3)

ensp00000248633	c.37	Adrenoleukodystrophy neonatal 202370 (3),

Refsum disease infantile 266510 (3),Zellweger syndrome-1 214100 (3)

ensp00000249505	a.4	Synpolydactyly type II 186000 (3)

ensp00000249760	e.6,a.29*	Isovalericacidemia (3)

ensp00000251020	b.121,g.37	Townes-Brocks syndrome 107480 (3)

ensp00000251654	c.14*	Propionicacidemia type II or pccB type (3)

ensp00000252857	b.1	Oguchi disease-1 258100 (3)

ensp00000253127	d.58	Dyskeratosis congenita-1 305000 (3)

ensp00000253260	c.2*	Pseudohermaphroditism male with gynecomastia (3)

ensp00000253496	b.47,g.14	Factor XII deficiency (3)

ensp00000253546	c.69*	Ceroid lipofuscinosis neuronal variant juvenile type with granular osmiophilic deposits (3),	Ceroid lipofuscinosis neuronal-1 infantile 256730 (3)

ensp00000254142	c.36*	Pyruvate dehydrogenase deficiency (3)

ensp00000254299	d.96*	Dystonia DOPA-responsive 128230 (3),

Phenylketonuria atypical due to GCH1 deficiency 233910 (1)

ensp00000254663	c.67*	Leukemia-1 T-cell acute lymphoblastic (3)

ensp00000254854	d.144,d.58 Cone dystrophy progressive (2),Leber congenital amaurosis type I 204000 (3)

ensp00000255266	a.125,d.110	Retinitis pigmentosa autosomal recessive (3)

ensp00000256216	c.2,d.106,d.38	D-bifunctional protein deficiency (3)

ensp00000256592	g.17*	Hypothyroidism nongoitrous (3)

ensp00000256635	c.45	Lymphoproliferative syndrome X-linked (2)

ensp00000256996	b.69	Xeroderma pigmentosum group E DDB-negative subtype 278740 (3)

ensp00000257340	c.120	Xeroderma pigmentosum group G 278780 (3)

ensp00000257555	a.35,a.4,a.34	Insulin-dependent diabetes mellitus (3),

MODY type 3 600496 (3),Non-insulin-dependent diabetes mellitus-2 601407 (2)

ensp00000257566	b.2	Ulnar-mammary syndrome 181450 (3)

ensp00000257904	d.144*	Melanoma (3)

ensp00000258224	e.1	{Hypertension essential susceptibility to} (3),{Preeclampsia susceptibility to} (3)

ensp00000259398	c.1*	Fructose intolerance (3)

ensp00000259463	a.6,g.39	Xeroderma pigmentosum group A (3)

ensp00000260630	a.104*	Glaucoma 3A primary infantile 231300 (3)

ensp00000261007	f.36,b.96	Myasthenic syndrome slow-channel congenital 601462 (3)

ensp00000261170	d.58,d.144	Lipoid adrenal hyperplasia 201710 (3)

ensp00000261267	d.2*	Amyloidosis renal 105200 (3)

ensp00000261416	c.1,d.92	Sandhoff disease infantile juvenile and adult forms (3),

Spinal muscular atrophy HEXB-related (3)

ensp00000261967	b.47,g.3,g.32,g.3.1	Factor VII deficiency (3)

ensp00000261978	g.23,g.3;g.3.1	Marfan syndrome atypical (3)

ensp00000261981	e.1*	Alpha-1-antichymotrypsin deficiency (3),Cerebrovascular disease occlusive (3)

ensp00000262602	c.1*	Hemolytic anemia due to ADA excess (1),

Severe combined immunodeficiency due to ADA deficiency (3)

ensp00000263374	b.42	Central core disease 117000 (3),{Malignant hyperthermia susceptibility 1} 145600 (3)

ensp00000264870	d.3,b.1	Factor XIIIA deficiency (3)

ensp00000264914	c.76*	Maroteaux-Lamy syndrome several forms (3)

ensp00000264932	c.3,d.168,a.7,c.3	Leigh syndrome (3)

ensp00000265023	d.17	[Kininogen deficiency] (3)

ensp00000265172	b.47,g.12	C3b inactivator deficiency (3)

ensp00000265573	a.118	Sorsby fundus dystrophy 136900 (3)

ensp00000265833	d.16,c.3	Brunner syndrome (3)

ensp00000265838	c.95*	3-ketothiolase deficiency (3)

ensp00000265849	d.122,d.14	Colorectal cancer hereditary nonpolyposis type 4 (3),

Turcot syndrome with glioblastoma 276300 (3)

ensp00000266085	b.40*	Sorsby fundus dystrophy 136900 (3)

ensp00000266950	a.4	Pancreatic agenesis 260370 (3),Lacticacidemia due to PDX1 deficiency (3)

ensp00000267008	d.144	Hereditary hemorrhagic telangiectasia-2 600376 (3)

ensp00000267163	a.74	Bladder cancer 109800 (3),Osteosarcoma 259500 (2),

Pinealoma with bilateral retinoblastoma(2),Retinoblastoma (3)

ensp00000268058	g.44	Leukemia acute promyelocytic PML/RARA type (3)

ensp00000268097	c.1,d.92	GM2-gangliosidosis juvenile adult (3),Tay-Sachs disease (3),[Hex A pseudodeficiency] (1)

ensp00000268476	c.67	Tyrosinemia type II (3)

ensp00000268695	c.76	Mucopolysaccharidosis IVA (3)

ensp00000268704	c.37	Spastic paraplegia-7 (3)

ensp00000269305	b.2,a.53	Colorectal cancer 114500 (3),Li-Fraumeni syndrome (3)

ensp00000269385	b.34	Obestiy with impaired prohormone processing 600955 (3)

ensp00000269571	d.144,c.10,g.3	Sialidosis type I (3),Sialidosis type II (3)

ensp00000269980	c.36	Maple syrup urine disease type Ia (3)

ensp00000270142	b.1*	Amytrophic lateral sclerosis due to SOD1 deficiency 105400 (3)

ensp00000271064	d.3	Argininemia (3)

ensp00000271448	c.3	Porphyria variegata 176200 (3)

ensp00000271509	g.18	Factor XIIIB deficiency (3)

ensp00000271651	d.3	Pycnodysostosis 265800 (3)

ensp00000272425	d.108	Fabry disease (3)

ensp00000272852	c.56*	Coproporphyria (3),Harderoporphyrinuria (3)

ensp00000273480	g.44*	Oguchi disease-1 258100 (3)

ensp00000274376	a.116,d.93,b.55	Basal cell carcinoma (3)

ensp00000274576	b.96	Hyperekplexia and spastic paraparesis (3),Startle disease autosomal recessive (3),

Startle disease/hyperekplexia autosomal dominant 149400 (3)

ensp00000274813	c.1,c.23*	Methylmalonicaciduria mutase deficiency type (3)

ensp00000276449	d.129*	Lipoid adrenal hyperplasia 201710 (3)

ensp00000277268	d.109	Amyloidosis Finnish type 105120 (3)

ensp00000277315	c.1*	Porphyria acute hepatic (3),{Lead poisoning susceptibility to} (3)

ensp00000277865	c.58,c.2	Hyperinsulinism-hyperammonemia syndrome (3)

ensp00000277940	a.64	Gaucher disease variant form (3),

Metachromatic leukodystrophy due to deficiency of SAP-1 (3)

ensp00000278017	a.104*	Adrenal hyperplasia congenital due to 17-alpha-hydroxylase deficiency (3)

ensp00000278715	c.94,d.50*	Porphyria acute intermittent (3)

ensp00000280362	d.96*	Phenylketonuria due to PTS deficiency (3)

ensp00000280496	a.4	Rhabdomyosarcoma alveolar 268220 (3)

ensp00000280696	a.123,g.39	Thyroid hormone resistance 274300 188570 (3)

ensp00000280867	d.130	Hypermethioninemia persistent autosomal dominant due to methionine

adenosyltransferase I/III deficiency (3)

ensp00000281243	c.2*	Phenylketonuria due to dihydropteridine reductase deficiency (3)

ensp00000281330	a.123,g.39 Pseudohypoaldosteronism type I autosomal dominant 177735 (3)

ensp00000283871	b.82*	Alkaptonuria (3)

ensp00000285379	b.74*	Renal tubular acidosis-osteopetrosis syndrome (3)

ensp00000289041	d.133,d.145,d.41,d.87,a.56,d.15	Xanthinuria type I (3)

ensp00000289473	d.189,b.34	Chronic granulomatous disease due to deficiency of NCF-1 (3)

ensp00000290524	a.4	Bare lymphocyte syndrome complementation group C (1)

ensp00000292144	b.1	Immunodeficiency due to defect in CD3-gamma (3)

ensp00000292327	a.39*	Cardiomopathy hypertrophic mid-ventricular chamber type (3)

ensp00000292427	a.104	Adrenal hyperplasia congenital due to 11-beta-hydroxylase deficiency (3),

Aldosteronism glucocorticoid-remediable(3)

ensp00000293288	f.1*	Colorectal cancer (3)

ensp00000293780	f.36	Myasthenic syndrome slow-channel congenital 601462 (3)

ensp00000294066	d.144	Hyperinsulinism familial 602485 (3),MODY type 2 125851 (3)

ensp00000294339	a.38	Leukemia-1 T-cell acute lymphoblastic (3)

ensp00000294782	a.77*	Albinism ocular autosomal recessive (3),Albinism oculocutaneous type II (3)

ensp00000294954	f.13,c.10	Leydig cell hypoplasia (3),Precocious puberty male 176410 (3)

ensp00000295382	a.39*	Persistent Mullerian duct syndrome type I 261550 (3)

ensp00000295600	a.38	Tietz syndrome 103500 (3),

Waardenburg syndrome type IIA 193510 (3),

Waardenburg syndrome/ocular albinism digenic 103470 (3)

ensp00000295754	d.144,g.7	Colon cancer (3),Colorectal cancer familial nonpolyposis type 6 (3)

ensp00000296271	f.13*	Night blindness congenital stationery rhodopsin-related (3),

Retinitis pigmentosa autosomal recessive (3),Retinitis pigmentosa-4 autosomal dominant (3)

ensp00000297137	b.95*	GM2-gangliosidosis AB variant (3)

ensp00000297283	c.60*	Myopathy due to phosphoglycerate mutase deficiency (3)

ensp00000298467	d.144,d.93,b.34	Leukemia chronic myeloid (3)

ensp00000298556	c.61*	HPRT-related gout (3),Lesch-Nyhan syndrome (3)

ensp00000299299	d.74*	Hyperphenylalaninemia due to pterin-4a-carbinolamine dehydratase deficiency 264070 (3)

ensp00000301873	g.23,g.3	Marfan syndrome atypical (3)

ensp00000302036	c.55*	Glycerol kinase deficiency (3)

ensp00000302237	c.37,a.66	McCune-Albright polyostotic fibrous dysplasia 174800 (3),

PituitaryACTH secreting adenoma (3),

Pseudohypoparathyroidism type Ia 103580 (3),Somatotrophinoma (3)

ensp00000302257	c.55	Hemolytic anemia due to hexokinase deficiency (3)

ensp00000302390	a.123	Adrenal hypoplasia congenital with hypogonadotropic hypogonadism (3)

ensp00000302728	b.18,c.1,b.1	Mucopolysaccharidosis VII (3)

ensp00000302777	c.32,d.79	[Red hair/fair skin] (3),{UV-induced skin damage vulnerability to} (3)

ensp00000302942	a.4	Schizencephaly (3)

ensp00000303500	d.178,d.58	Phenylketonuria (3),[Hyperphenylalaninemia mild] (3)

ensp00000303532	g.9*	Gaucher disease variant form (3),

Metachromatic leukodystrophy due to deficiency of SAP-1 (3)

ensp00000303875	a.104*	Adrenal hyperplasia congenital due to 21-hydroxylase deficiency (3),

Carbamoylphosphate synthetase I deficiency (3)

ensp00000304290	f.36,b.96	Myasthenic syndrome slow-channel congenital 601462 (3)

ensp00000304973	c.45,b.7*	Bannayan-Zonana syndrome 153480 (3),Cowden disease 158350 (3),

Endometrial carcinoma (3),Lhermitte-Duclos syndrome (3)

ensp00000306099	d.171	Dysfibrinogenemia beta type (3)

ensp00000306361	d.171	Amyloidosis hereditary renal 105200 (3),

Dysfibrinogenemia alpha type causing bleeding diathesis (3),

Dysfibrinogenemia alpha type causing recurrent thrombosis (3)

ensp00000306477	d.160	Biotinidase deficiency (3)

ensp00000307786	a.3*	Multiple carboxylase deficiency biotin-responsive (3)

ensp00000308024	c.41,b.18,d.58	Obestiy with impaired prohormone processing 600955 (3)

ensp00000308554	d.142,c.30,b.84	Propionicacidemia type I or pccA type (1)

ensp00000308576	c.37*	Night blindness congenital stationery rhodopsin-related (3),

Retinitis pigmentosa autosomal recessive (3),Retinitis pigmentosa-4 autosomal dominant (3)

ensp00000308619	g.50	Alpha-thalassemia/mental retardation syndrome type 2 301040 (3),

Juberg-Marsidi syndrome 309590 (3)

ensp00000309757	c.69	Chylomicronemia syndrome familial (3),

Combined hyperlipemia familial (3),Hyperlipoproteinemia I (1),

Lipoprotein lipase deficiency (3)

ensp00000309845	c.37*	Bladder cancer 109800 (3)

ensp00000311280	a.4	Familial Mediterranean fever (3)

ensp00000311290	a.4	Pituitary hormone deficiency combined (3)

ensp00000312673	a.26*	Isolated growth hormone deficiency Illig type with absent GH

and Kowarski type with bioinactive GH (3)

ensp00000312999	c.37,a.66	Pituitary ACTH-secreting adenoma (3)

ensp00000313026	c.2*	Galactose epimerase deficiency (3)

ensp00000313547	a.118,b.34	Chronic granulomatous disease due to deficiency of NCF-2 (1)

ensp00000313925	a.123,g.39	Androgen insensitivity several forms (3),

Breast cancer male with Reifenstein syndrome (3),Perineal hypospadias (3),

Prostate cancer (3),Spinal and bulbar muscular atrophy of Kennedy 313200 (3)

ensp00000314606	c.76*	Sanfilippo syndrome type A (3)

ensp00000315029	d.169,g.3	Thrombophilia due to thrombomodulin defect (3)

ensp00000315680	b.69*	Rhizomelic chondrodysplasia punctata type 1 215100 (3)

ensp00000316786	c.2	Apparent mineralocorticoid excess hypertension due to (3)

ensp00000317862	b.55,a.68	Thrombocytopenia X-linked 313900 (3),Wiskott-Aldrich syndrome (3)

ensp00000318318	b.82*	Carbohydrate-deficient glycoprotein syndrome type Ib 602579 (3)

ensp00000318351	c.36,c.48	Maple syrup urine disease type Ib (3)

ensp00000318568	b.47*	Pancreatitis hereditary 167800 (3),Trypsinogen deficiency (1)

ensp00000319034	b.1	Kostmann neutropenia 202700 (3)

ensp00000319075	b.1	Immunodeficiency T-cell receptor/CD3 complex (3)

ensp00000319899	d.13;d.13*	Galactosemia (3)

ensp00000320187	c.1*	Aldolase A deficiency (3)

ensp00000320568	c.55	Glycerol kinase deficiency (3)

ensp00000321853	e.1	Plasmin inhibitor deficiency (3)

ensp00000321935	c.37	Griscelli disease 214450 (3)

ensp00000324856	d.144	Peutz-Jeghers syndrome 175200 (3)

ensp00000324895	g.9*	Gaucher disease variant form (3),

Metachromatic leukodystrophy due to deficiency of SAP-1 (3)

ensp00000325136	c.95*	Trifunctional protein deficiency type II (3)

ensp00000325831	d.178	Segawa syndrome recessive (3)

ensp00000327074	c.80*	Hemolytic anemia due to glucosephosphate isomerase deficiency (3),

Hydrops fetalis one form (1)

ensp00000327758	a.4	Atrial septal defect with atrioventricular conduction defects 108900 (3)

ensp00000328107	d.15	Parkinson disease juvenile type 2 600116 (3)

ensp00000328511	d.42,f.14	Hemolytic anemia due to hexokinase deficiency (3)

ensp00000328662	c.68	Chondrosarcoma 215300 (3),Exostoses multiple type 1 (3)

ensp00000329380	c.10	Bernard-Soulier syndrome (3)

ensp00000329869	a.93,g.18,g.3	Goiter congenital (3),Hypothyroidism congenital (3),

Thrombocythemia essential 187950 (3),Thyroid iodine peroxidase deficiency (1)

ensp00000329968	d.144	Glycogenosis hepatic autosomal (3)

ensp00000330382	g.17	Dermatofibrosarcoma protuberans (3),Giant-cell fibroblastoma (3),Meningioma SIS-related (3)

ensp00000331181	d.93*	Lymphoproliferative syndrome X-linked (2),{?Amyloidosis secondary susceptibility to} (1)

ensp00000332369	c.67	Anemia sideroblastic/hypochromic (3)

ensp00000334876	a.91,d.144*	Oguchi disease-2 258100 (3)

ensp00000335505	a.4	Langer mesomelic dysplasia 249700 (3),Leri-Weill dyschondrosteosis 127300 (3),

Short stature idiopathic familial (3)

ensp00000336575	c.35*	Hemolytic anemia due to glucosephosphate isomerase deficiency (3),

Hydrops fetalis one form (1)

ensp00000336709	d.142,b.84,c.30,b.84	Pyruvate carboxylase deficiency (3)

ensp00000336829	d.171	Dysfibrinogenemia gamma type (3),Hypofibrinogenemia gamma type (3)

ensp00000337247	d.144,b.1	Pfeiffer syndrome 101600 (3),Ichthyosis vulgaris 146700 (1) (?)

ensp00000338009	c.55	Hyperinsulinism familial 602485 (3),MODY type 2 125851 (3)

ensp00000338072	f.13	Diabetes insipidus nephrogenic (3)

ensp00000338387	d.104	Multiple carboxylase deficiency biotin-responsive (3)

ensp00000339824	d.144,b.1	Achondroplasia 100800 (3),Craniosynostosis nonsyndromic (3),

Crouzon syndrome with acanthosis nigricans (3),Hypochondroplasia 146000 (3),

Thanatophoric dysplasia types I and II 187600 (3)

ensp00000339996	d.6	Creutzfeldt-Jakob disease 123400 (3),Gerstmann-Straussler disease 137440 (3),

Insomnia fatal familial (3),5-oxoprolinuria 266130 (3),

Hemolytic anemia due to glutathione synthetase deficiency 231900 (3)

ensp00000340482	c.93	Hypocalcemia autosomal dominant 601198 (3),

Hypocalciuric hypercalcemia type I 145980 (3),Neonatal hyperparathyroidism 239200 (3)

ensp00000340690	b.2	Leukemia acute myeloid (3)

ensp00000341625	d.120*	Methemoglobinemia due to cytochrome b5 deficiency (3)

ensp00000342092	a.4	Craniofacial-deafness-hand syndrome 122880 (3),Rhabdomyosarcoma alveolar268220 (3),	Waardenburg syndrome type I (3),Waardenburg syndrome type III 148820 (3)

ensp00000342172	b.2.5	Cleidocranial dysplasia 119600 (3)

ensp00000342269	c.1*	Androgen insensitivity several forms (3),Breast cancer male with Reifenstein syndrome (3),	Perineal hypospadias (3),Prostate cancer (3),

Spinal and bulbar muscular atrophy of Kennedy 313200 (3)

ensp00000342297	a.4	Gaucher disease variant form (3),

Metachromatic leukodystrophy due to deficiency of SAP-1 (3)

ensp00000342568	g.17*	Hypogonadism hypergonadotropic (3),

Male pseudohermaphroditism due to defective LH (1) (?)

ensp00000343136	c.43,b.84,a.9	Maple syrup urine disease type II (3)

ensp00000343420	c.3.1,d.16	Choroideremia (3)

ensp00000343671	b.69	Cone dystrophy progressive X-linked 1 (2),Cone-rod retinal dystrophy-2 120970 (3),

Leber congenital amaurosis type III (3),Retinitis pigmentosa-15 (2),Retinitis pigmentosa-3 (3)

ensp00000343711	a.4.1,g.39	Nail-patella syndrome with open-angle glaucoma 137750 (3),

Nail-patella syndrome 161200 (3)

ensp00000343807	a.123,g.39	MODY type 1 125850 (3),Non-insulin-dependent diabetes mellitus 125853 (3)

ensp00000343888	d.122,d.14,a.21	Colorectal cancer hereditary nonpolyposis type 3 (3)

ensp00000343922	f.13	Hirschsprung disease-2 600155 (3)

ensp00000343937	c.76	Hypophosphatasia adult 146300 (1) (?),Hypophosphatasia infantile 241500 (3)

ensp00000344281	a.39*	Melanoma 155601 (3)

ensp00000344313	d.14,d.58*	Mevalonicaciduria (3)

ensp00000344379	b.82*	Alkaptonuria (3)

ensp00000344460	c.79	Homocystinuria B6-responsive and nonresponsive types (3)

ensp00000344708	c.68	Exostoses multiple type 2 (3)

ensp00000344751	b.69,b.1	Myopathy congenital (3)

ensp00000345166	a.39*	Melanoma 155601 (3)

ensp00000345169	a.77,g.24	{Autoimmune lymphoproliferative syndrome} (3)

ensp00000345489	a.127*	Argininosuccinicaciduria (3)

ensp00000345997	d.144	Myotonic dystrophy (3)

**Protocol S5: Trend Exceptions**

Although we've shown that folds predicted to be of greater designability tend to be more widespread within proteomes certain exceptions are known. These exceptions can be more readily spotted if one subdivides the set of folds into classes or if one examines individual folds or families (Supplementary Figure S23). For example, even with our strict method of SCOP fold detection (see Methods), the C2H2 zinc finger fold was found in over 500 human proteins, a much higher than expected quantity given that less than 5 families belonging to the fold are currently known (Supplementary Figure S12). Although it seems that the zinc finger fold is somewhat constrained in its ability to sequence divergence (probably due to its functional involvement in molecular interactions), the need for functions carried out by such fold in eukaryotic genomes has maintained its high abundance.

We also profiled SCOP folds across genomes. SCOP folds with greater numbers of families appeared more often in a set of arbitrarily selected proteomes (Supplementary Figure S24), consistent with the idea that more designable folds should be more widespread. In contrast, we found that families belonging to folds with greater numbers of families were not present more often across the same proteomes. These results suggest that individual selective pressures for functions carried out by certain SCOP families also influence the abundance and appearance of these families in organisms.
